# Mapping Local Environments in Single-Atom Catalysts

**DOI:** 10.1021/acs.chemrev.6c00373

**Published:** 2026-10-16

**Authors:** Andrea Ruiz-Ferrando, Sharon Mitchell, Javier Pérez-Ramírez

**Affiliations:** † Institute for Chemical and Bioengineering, Department of Chemistry and Applied Biosciences, 27219 ETH Zurich, Vladimir Prelog Weg 1, 8093 Zurich, Switzerland; ‡ NCCR Catalysis, 8093 Zurich, Switzerland

## Abstract

Single-atom catalysts (SACs) offer exceptional structural resolution for investigating how local environments govern catalytic behavior and, ultimately, for controlling these effects through catalyst design. Yet, atomic dispersion does not imply a single or fully defined catalytic environment. The behavior of atomically dispersed active centers is shaped by a hierarchical local environment comprising persistent and transient contributions, while also being influenced by an extended reaction environment that intersects with the local environment in controlling reactivity and can become integrated into it. Establishing these boundaries and understanding how these contributions evolve and interact are central challenges in the interpretation and design of SACs. Here, we critically examine how local environments are generated and controlled, experimentally and theoretically resolved across spatial and temporal scales, transformed from synthesis through operation and deactivation, and linked to catalytic performance through structural, energetic, and electronic descriptors. By integrating aspects that are often considered separately and highlighting those that remain insufficiently addressed, we provide a roadmap toward the predictive, environment-driven design of SACs and a deeper understanding of catalytic phenomena.

## Graphical abstract

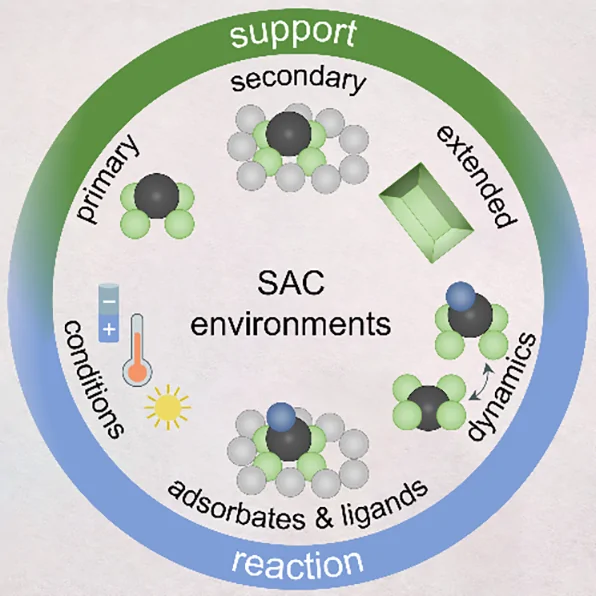

## 1 Introduction

### 1.1 Local Environments and Active Sites in Heterogeneous Catalysis

Since Taylor’s early articulation of the active site concept,^1^ it has been widely recognized that catalytic function in heterogeneous materials is governed by local atomic environments rather than by the bulk catalyst alone.^2,3^ Activity, selectivity, and stability are determined by the immediate coordination of active centers, their interaction with the surrounding support or matrix, and the local chemical landscape shaped by defects, ligands, and adsorbates. The ability to control such environments with atomic precision is therefore not only of fundamental interest but of direct practical relevance for the rational design of more active, selective, and stable catalysts.^4^

Despite this long-standing recognition, establishing clear and predictive structure–performance relationships in heterogeneous catalysis has remained persistently challenging.^5^ The central difficulty lies in the intrinsic complexity of the conventional catalyst materials. Active sites are typically embedded within structurally and chemically heterogeneous solids, where multiple coordination motifs coexist across different facets, edges, and defect sites, often even within a single nanoparticle (Figure 1a).^6^ This heterogeneity is further compounded by dynamic restructuring under reaction conditions, which continuously alters the local environment of active centers.^7–9^

**Figure 1:**
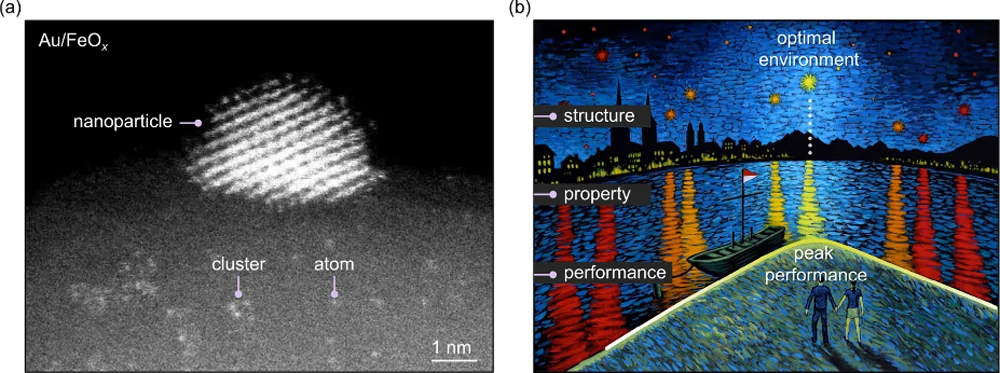
(a) High-angle annular dark-field (HAADF-STEM) image illustrating the heterogeneous speciation of a traditional supported metal catalyst, with the coexistence of nanoparticles, clusters, and isolated atoms. (b) Conceptual representation of catalyst development as navigation toward an optimal local environment guided by indirect structural and physicochemical descriptors. Part (a) adapted with permission from ref 17 (Copyright 2009, Oxford University Press).

At the same time, the synthetic control required to define specific site motifs has historically been limited, and the analytical sensitivity necessary to quantitatively resolve the distribution and individual contributions of these motifs to catalytic performance remains insufficient.^10,11^ Consequently, the local environments responsible for reactivity remain largely inaccessible to direct experimental observation, hindering their rational optimization. Catalyst design has therefore relied on inferring these environments from experimentally accessible structural, spectroscopic, and catalytic observations. These complementary signatures provide distinct reflections of the underlying catalytic environments, allowing experiments and theory to guide the search toward the optimal environment (Figure 1b). Although such approaches have been invaluable in advancing the field, they have also inevitably sacrificed the diversity and adaptability of real catalytic environments, thereby limiting the development of robust and predictive design principles.

Classical descriptions of heterogeneous catalysis have consequently approached this complexity from different directions. Early adsorption models established how molecules populate sites on solid surfaces, while subsequent kinetic and mechanistic treatments described reactions through the populations and transformations of adsorbed species.^12–15^ Reaction engineering approaches later established how mass and heat transport can modify the conditions experienced by catalytic sites and the resulting observable kinetics.^16^ These complementary perspectives have been foundational to catalysis, but they typically resolve only part of the environment governing an active center. Connecting the structure of the solid, its adsorbed species, dynamic changes in coordination and electronic state, and the surrounding reaction environment within a common description remains considerably more difficult.

Recent advances in catalyst synthesis and characterization have begun to partially address these limitations, enabling increasingly precise control over local coordination environments and improved resolution of their structure and distribution at the atomic scale.^18–21^ Of the various approaches that have been developed, the isolation of catalytically active elements as individual atoms on solid supports has gained particular interest, owing to the prospect of minimizing ensemble complexity and enhancing the impact of the surrounding environment.^22^ In this regard, single-atom catalysts (SACs) provide a particularly tractable design platform for connecting these different descriptions around atomically dispersed active centers, enabling increasingly detailed examination of how local environments emerge, evolve, and govern catalytic function.^23^

### 1.2 Emergence and Consolidation of Single-Atom Catalysts

SACs are materials in which catalytically active metal centers are present as isolated atoms stabilized on a solid support (Figure 2a). While isolated metal atoms had been proposed as active sites earlier, most notably by Maria Flytzani-Stephanopoulos and co-workers in studies of ionic gold and platinum species on oxide supports,^3^ the field lacked the tools to establish SACs as a distinct class. With the growing accessibility of transmission electron microscopy (TEM), particularly in its aberration-corrected scanning transmission mode (AC-STEM), the reliable visualization of isolated atoms has become possible.^24^ In this context, a seminal report by Jingyue Liu, Jun Li, Tao Zhang, and co-workers^25^ introduced the term *single*-*atom catalyst* and demonstrated that isolated platinum atoms (0.1 wt %) stabilized on iron oxide exhibited high carbon monoxide oxidation activity. This study provided a clear structure–performance correlation for isolated atoms and helped catalyze the rapid growth in SAC research.^22,26–29^

**Figure 2:**
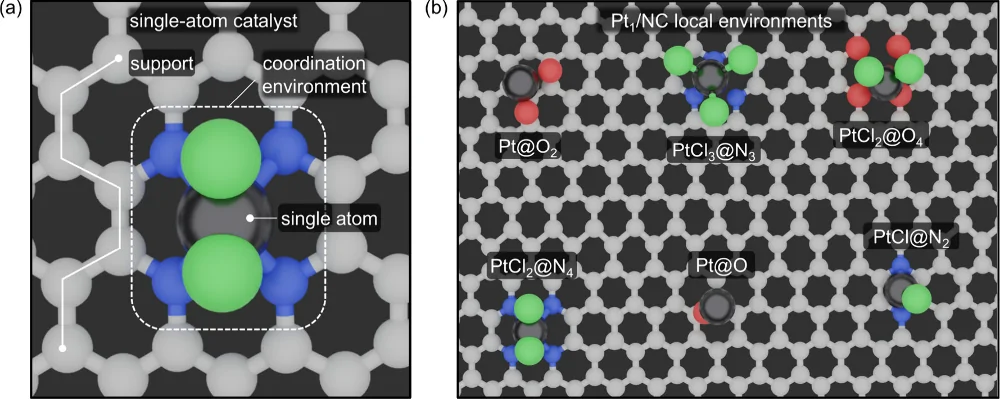
(a) Idealized depiction of the local environment of a platinum single atom on nitrogen-doped carbon (Pt_1_/NC), illustrating the fundamental components of an isolated catalytic site. (b) Schematic representation of a population of possible local environments simulated for Pt_1_/NC, showing different coordination motifs within a single SAC (e.g., Pt@O_2_, PtCl_3_@N_3_).

Early studies asked whether individual atoms could remain stable under realistic chemical and thermal conditions and act as catalytically active sites. They showed that single metal atoms can be anchored at specific surface sites, such as defects or heteroatom-rich domains, without sintering, leaching, or volatilizing.^30^ Recent advances in synthetic strategies, including wet-chemical routes, high-temperature treatments, vapor-phase deposition, and template-assisted approaches, have made it possible to prepare SACs of many different elements on diverse supports, such as oxides, functionalized carbons, nitrides, sulfides, and hybrid materials.^31,32^

At the same time, progress in characterization methods has been important for establishing SACs as a distinct catalyst class.^33^ By combining AC-STEM, X-ray absorption spectroscopy (XAS), electron paramagnetic resonance spectroscopy (EPR), and infrared spectroscopy (IR) as complementary probes, compelling evidence for atomic dispersion and local coordination has been obtained. The increasing use of operando and in situ techniques to study SACs under working conditions has also reinforced their relevance beyond idealized structures, helping further support the link between site architecture and performance.^34^

As research on SAC has grown, the range of catalytic applications has also expanded. SACs have been reported for thermal, electrochemical, and photocatalytic reactions, covering oxidation and hydrogenation, the activation and formation of C–X bonds (X = C, N, O, S, H), electrocatalytic reactions for energy conversion, and other processes relevant for sustainable chemicals manufacture.^35^ Notably, SACs have been explored in industrially relevant reactions, including acetylene hydrochlorination^36^ and carbon dioxide hydrogenation to methanol,^37^ with competitive performance. At the same time, the chance to utilize scarce elements like precious metals more efficiently has made SACs particularly appealing from a sustainability perspective.^37–40^

By 2020, the accumulated body of work had built up to justify critical assessment.^26^ At that point, reviews helped to formalize definitions,^28^ classify synthetic routes and supports,^41^ and summarize early structure–performance relationships across different reaction classes.^42^ Today, research on SACs has moved past asking whether isolated atoms can be stabilized or whether they can catalyze useful reactions, which has been well-established by evidence.^43^ Instead, they have become a widely adopted platform for exploring catalytic phenomena at the atomic scale, because they provide access to mononuclear.^44^ Although often represented by an idealized coordination environment (Figure 2a), SACs exhibit intrinsic coordination heterogeneity. For example, platinum single atoms on a nitrogen-doped carbon (NC) support (denoted as Pt_1_/NC) adopt a range of local coordination environments, such as Pt@O_2_ or PtCl@N_2_ (Figure 2b). This inherent diversity enables the systematic exploration of how variations in the local environment govern the catalytic behavior.

### 1.3 From Defining Local Environments to Mapping their Dynamics and Reactivity

Although SACs avoid the complexity of metal ensembles by isolating active centers as individual atoms, they still operate within chemically rich environments that strongly influence catalytic behavior. An isolated atom therefore never acts independently but is continuously modulated by structurally and chemically diverse surroundings.^45^ These environments may vary across conditions and stages of catalyst use, linking synthesis, operation, and deactivation within a broader catalyst lifecycle.^46–48^ Against this backdrop of intrinsic complexity, a fundamental challenge remains: how to rigorously define and characterize local environments and relate them to catalytic performance.^49^

In practice, this complexity is often simplified by treating local environments as static and uniform coordination environments, for example, through formal oxidation states, coordination numbers, or schematic M@N_*x*_ and M@O_*x*_ motifs.^50,51^ These descriptors, frequently derived from techniques such as XAS using single fits or structural models, have become the dominant language for rationalizing activity and selectivity trends. Although they enable us to propose plausible atomic structures and mechanisms, such representations often implicitly assume structural uniformity, temporal persistence, and a one-to-one correspondence between an assigned motif and the catalytic function.

This mismatch between the dynamic evolution of local environments and their predominantly static description has important consequences.^49^ Mechanistic interpretations may mix up spectator and active structures on the surface, deriving structure–performance correlations from averaged or metastable states rather than those actually taking part in the reaction. This can result in issues with the reproducibility of catalytic results, with differences between studies arising from the presence of distinct types of sites that are not captured through analytical methods. In this context, even with increasing operando and in situ data becoming available, the main issue remains. The field still lacks a consistent approach for considering structural changes over time, the presence of diverse local environments, and how these factors relate to the catalytic function.

Addressing this challenge requires re-evaluating the assumptions that underpin current analyses. SACs should not be treated as monodisperse collections of identical active sites. Instead, the relevant descriptor is the local environment of each atom, including its coordination geometry, electronic structure, and nearby (e.g., secondary-sphere) interactions, as well as how these features evolve over time. Developing approaches that explicitly account for the diversity and evolution of local environments is essential to improving data interpretation, enabling predictive catalyst design, and fully exploiting the potential of SAC across diverse reaction environments.

### 1.4 Scope and Approach

This Review addresses the challenge of defining, resolving, and ultimately controlling local environments in single-atom catalysts. Rather than surveying SAC materials, methods, or applications as separate topics, we use the local environment as an organizing framework to connect synthesis, characterization, theory, and catalytic function. Our aim is to establish what constitutes this environment, where its boundaries should be drawn, how it relates to the extended reaction environment, and how persistent and transient states together determine the reactivity of the atomically dispersed active center.

Many of the phenomena that shape SAC behavior are shared across heterogeneous catalysis, including adsorbates, solvation, support interactions, mass and charge transport, photon delivery, and dynamic restructuring. SACs nevertheless provide an unusually tractable platform in which these effects can be structurally resolved, modeled, and connected to the catalytic function.

We consider local environments to be heterogeneous and evolving distributions rather than as unique, static coordination motifs. We first establish a framework for describing their structural, electronic, and spatial characteristics and distinguish the local environment from the extended environment that interacts with and can, under certain conditions, become incorporated into it. We then examine how experimental and theoretical tools can resolve these environments across relevant spatial and temporal scales, emphasizing complementary operando and time-resolved approaches. Synthetic strategies are considered in terms of how they bias or constrain environment distributions rather than as isolated preparation routes.

A central focus is on how local environments evolve throughout the catalyst lifecycle (Figure 3). We distinguish persistent structural features from transient contributions that emerge during activation and reaction and consider how their interplay shapes catalytic mechanisms, stability, deactivation, and regeneration. We then ask how this evolving structural picture can be translated into measurable descriptors and, ultimately, design principles. Representative case studies across thermal, electrochemical, and photocatalytic systems illustrate these concepts, while parallels to homogeneous and enzymatic catalysis provide additional context.

**Figure 3:**
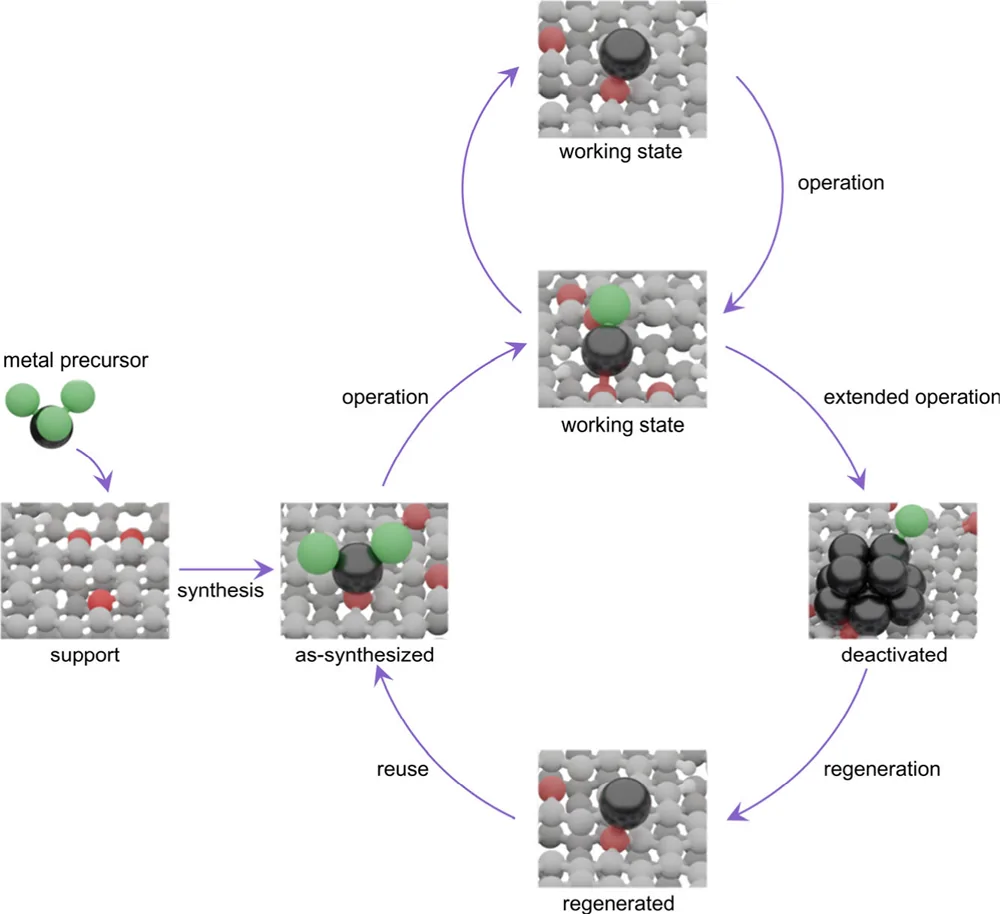
Evolution of SAC local environments throughout the catalyst lifecycle, highlighting how synthesis and activation shape the catalytic structure expressed under operating conditions and its subsequent evolution during deactivation and regeneration.

By following the local environment from its formation through its characterization, evolution, catalytic function, and eventual redesign, this Review provides a framework for integrating observations that are often considered separately. The broader objective is to move beyond the assignment of idealized single-site structures toward a predictive description of catalytic environments and, ultimately, their deliberate engineering.

## 2 Defining Local Environments in Single-Atom Catalysis

SACs are often described in terms of isolated metal centers, yet their behavior is governed by the surrounding environment in which these atoms are embedded. This section establishes the local environment as the catalytically relevant entity (Section 2.1) and frames it as a multiscale construct, spanning primary, secondary, and extended environments whose features may be catalyst-derived or condition-dependent (Section 2.2). Within this view, structural and electronic descriptors provide a means to represent and analyze these environments in relation to catalytic performance (Section 2.3). Local environments are then shown to be inherently dynamic and often metastable under reaction conditions (Section 2.4), evolving throughout the catalyst lifetime as sites form, transform, and deactivate (Section 2.5). These concepts are contextualized through parallels with homogeneous and enzymatic catalysis (Section 2.6), highlighting shared principles in how defined environments control reactivity, while emphasizing the additional complexity introduced by heterogeneity and adaptability in supported systems.

### 2.1 The Local Environment as the Catalytically Relevant Entity

Active sites in heterogeneous catalysis have traditionally been assigned to specific atomic sites or recurring structural features on catalytic surfaces.^6^ In SACs, this idea is often narrowed down to the specific metal atom present and how it is coordinated to the host material. While this atom-centered representation provides a useful starting point for establishing structure–property–performance relationships, it does not fully capture the nature of the catalytic entity.

The catalytically relevant unit is better described as the local environment surrounding the metal center.^27,45,52,53^ Its description cannot be restricted a priori to the metal and its immediate coordination sphere as more distant and condition-dependent contributions can also determine its catalytic state. In practice, however, the local environment is often represented primarily by the metal atom and its first coordination sphere, even when it is embedded in a periodic or extended support model for spectral interpretation and atomistic simulations. This reduction has been essential for making SAC experimentally and computationally tractable but also imposes two important limitations. First, it can obscure secondary and longer-range contributions from defects, heteroatoms, confinement, support electronics, adsorbates, or interfacial species that may strongly influence the reactivity. Second, it often treats the catalyst as if it contains a single representative structure, although multiple closely related environments may coexist. This is particularly important because even when one coordination motif dominates ensemble-averaged characterization, it cannot be assigned as the catalytically operative site.

Spectroscopic measurements often preferentially detect abundant or stable species, including resting states or spectators, while more active minority environments may remain difficult to resolve. Well-defined SAC systems may therefore comprise multiple related first-sphere configurations beyond a single reported structure (Figure 4).^54,55^ SACs are therefore more accurately described as ensembles of related environments, with the measured catalytic performance reflecting the weighted contributions of these environments rather than the behavior of a single structural motif.^56^ This Perspective helps rationalize why catalysts with apparently similar nominal structures may exhibit markedly different activity or selectivity and underscores that recognizing this distribution of environments provides the conceptual foundation for interpreting structure–performance relationships in SAC systems.

**Figure 4:**
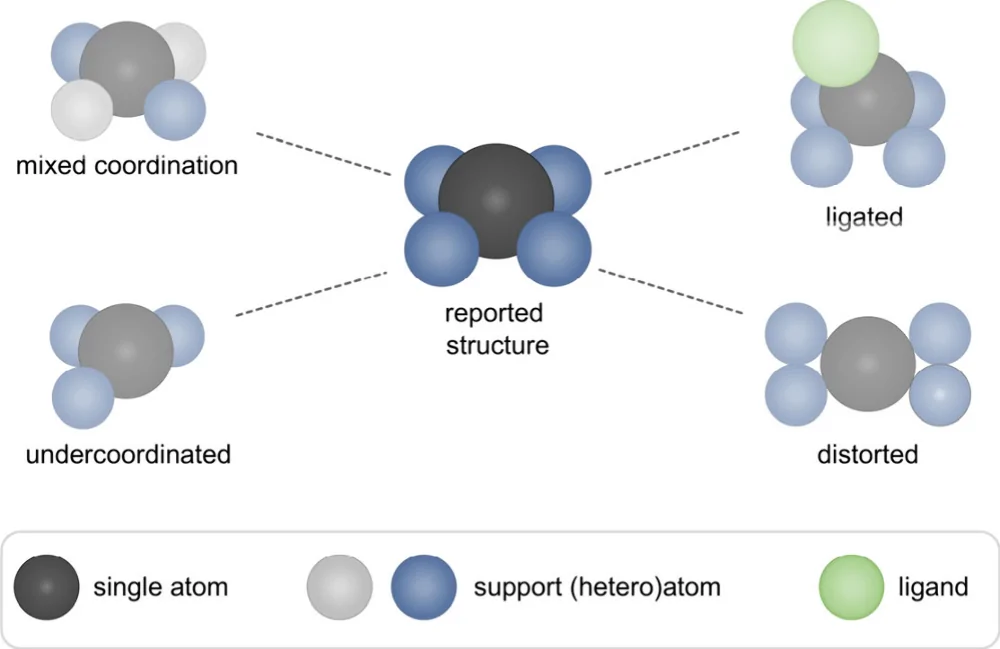
Schematic representation of a typically reported first-sphere coordination structure for a SAC and related environments not captured by a single structural assignment.

### 2.2 Hierarchies of Locality: Near, Secondary, and Extended Environments

After identifying the local environment as the catalytically relevant unit, two fundamental questions arise: which surrounding features should be considered part of that environment, and how should their influence be organized? These questions cannot be answered by the physical distance alone. In extended solids, the catalytic influence depends on bonding, support topology, electronic coupling, steric organization, defect structure, confinement, and the persistence of interactions under operating conditions. The same physical separation may therefore have different chemical significance in an oxide lattice, a doped carbon, a porous framework, or an electrochemical interface.^27^ Primary, secondary, and extended environments are therefore distinguished according to their relationship with the isolated metal center without imposing fixed spatial boundaries.

The immediate coordination sphere nevertheless provides the natural starting point. In coordination chemistry, as formalized by Werner’s theory,^57^ the arrangement of ligands around a metal center defines its geometric and electronic character. In SAC, this concept translates to the primary coordination sphere, which comprises the atoms directly bonded to the isolated atom. This sphere defines the local coordination geometry, ligand field, and bonding interactions that largely determine adsorption modes and intrinsic activation barriers. Typical structural motifs for metal (M) SACs include M@N_*x*_, M@O_*x*_, M@C_*x*_, mixed M@N/O, or M@O/C coordination. The same primary motifs may arise in distinct structural contexts, for example at vacancies, edges, defect sites, pore environments, or oxide and framework lattice positions.^27,45,50,51,58–61^

Beyond this primary coordination sphere lies the secondary environment, which includes nearby features that influence the properties of the single atom without being directly bonded to it. These features are site-specific and are locally organized around it. Representative motifs include heteroatom arrangements adjacent to the primary coordination sphere, B-, S-, P-, or O-modified M@N_*x*_ sites, vacancy-metal or hydroxyl-metal ensembles, acid–base pairs near the single atom, pore-confined pockets, neighboring single-atom or dual-atom arrangements, and adsorbate, solvent, or electrolyte structures forming around the site.^27,52,62–66^ Both the primary and secondary environments may be inherited from synthesis or generated during activation and operation, for example, through hydroxylation, defect formation, adsorbate organization, or electrolyte restructuring. The primary and secondary environments together define the most directly assignable parts of the local environment.

At larger length scales, the extended environment encompasses broader features and properties of the support or interface that influence the metal center beyond its site-specific surroundings.^27,45,52^ Relevant contributions include support morphology, crystallinity, porosity, defect distributions, and broader interfacial organization as well as structural and electronic properties such as lattice strain, confinement, conductivity, reducibility, and band structure.^60,66^

The distinction among the primary, secondary, and extended environments is based on the nature and local specificity of their relationship with the metal center rather than on fixed spatial boundaries. Atoms directly coordinated to the metal inherently constitute the primary environment, because their bonding interactions define its structural and electronic state. Beyond this first coordination sphere, spatial proximity alone does not establish membership in the local environment: a feature must meaningfully influence the state or behavior of the metal center. Secondary environment features are discrete or locally organized around a particular metal center and influence it despite lacking direct coordination. Extended-environment contributions are distinguished by the absence of such site-specific local organization; their influence on the metal instead reflects broader or distributed properties of the support or interface.

This distinction is necessary because properties such as lattice strain, reducibility, band structure, confinement, or collective interfacial organization emerge over larger regions of the catalyst or interface and cannot be meaningfully classified by their distance from the metal surface. Experimental detectability provides evidence for assigning a feature rather than defining its level, while involvement during the reaction establishes catalytic relevance without necessarily determining its position within the hierarchy. Thus, an adsorbate, hydroxyl group, or electrolyte species may belong to the primary environment if it coordinates directly to the metal, to the secondary environment if it is specifically organized around and influences the site, or to the extended environment if its influence arises through the broader interface or support. Conversely, operating variables such as temperature, pressure, applied potential, illumination, or bulk reaction-medium composition are not themselves components of the local environment but can generate or modify its features.

Primary, secondary, and extended define how features of the local environment relate to the metal center, independently of whether those features derive from the catalyst support, develop under activation or operating conditions, or reflect contributions from both (Figure 5).^27,52,62^ Catalyst-derived features may originate from the support or matrix, promoters, ligands, additives, or other components of the catalyst formulation, whereas condition-dependent features emerge or evolve during activation and operation. These contributions are not mutually exclusive: operating conditions can restructure the support, and the support determines how adsorbates, solvents, electrolyte species, or other reaction-derived components organize around the metal center. As a result, the local environment can evolve throughout activation, operation, deactivation, and regeneration through changes in coordination, hydroxylation, defect structure, adsorbate coverage, or interfacial organization.

**Figure 5:**
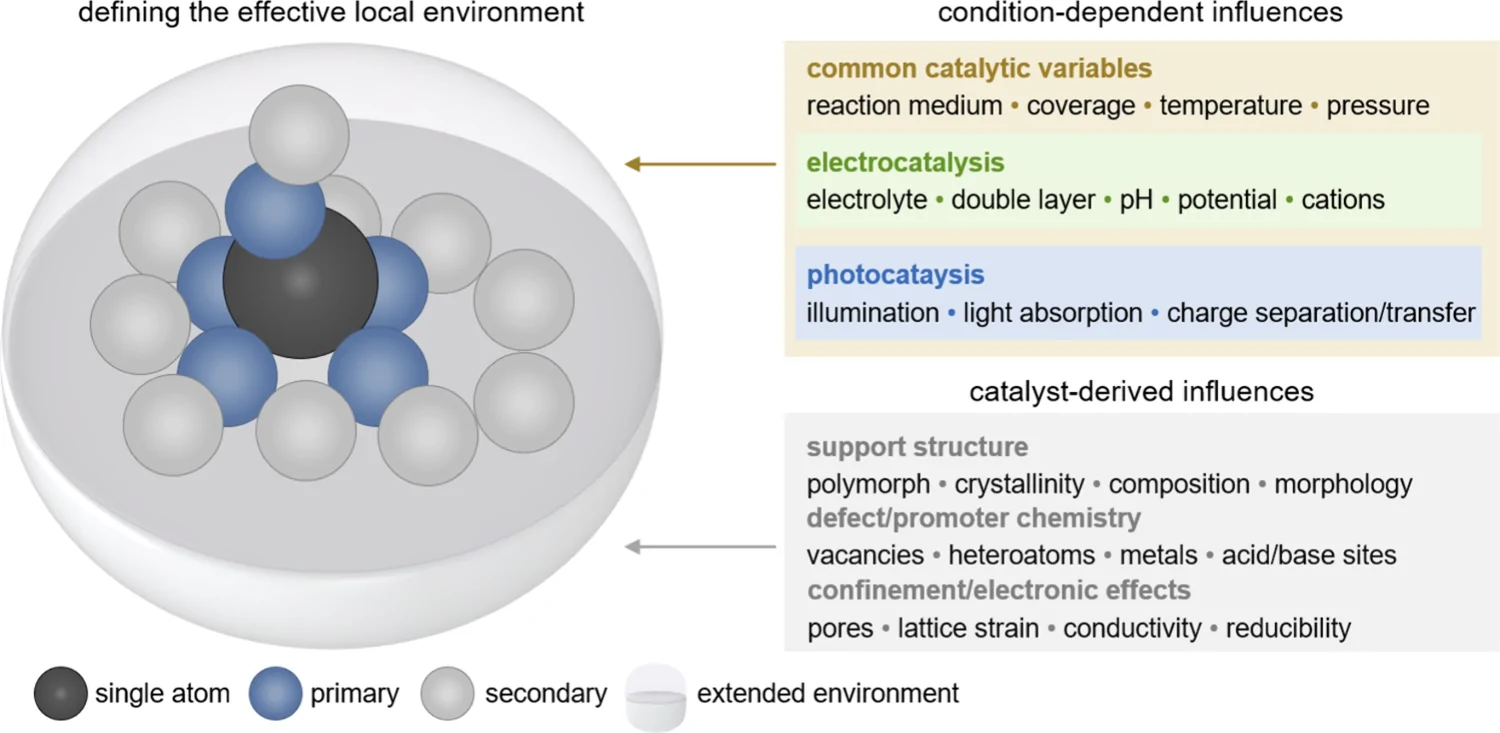
Primary, secondary, and extended environments describe increasing degrees of indirect influence on the isolated metal center. Catalyst-derived and condition-dependent influences can contribute at different levels depending on bonding, local organization, persistence, and catalytic relevance.

The same operational hierarchy can therefore be applied across thermal, electrochemical, and photocatalytic regimes, while the physical contributions that shape the local environment differ depending on the reaction context. In thermal catalysis, the reaction atmosphere, temperature, and pressure can modify adsorbate coverage, surface composition, defect states, and reconstruction pathways. In electrocatalysis, the electrolyte composition, ionic strength, local pH, cations, and applied potential can additionally shape site-associated solvation, ionic organization, interfacial electric fields, and adsorbate states.^67^ In photocatalysis, illumination can reshape the local environment through photogenerated carrier generation, separation, and transfer and the associated changes in electronic and interfacial states.^68^ Across these regimes, such features can contribute to the primary, secondary, or extended environment depending on how they relate to the metal center, irrespective of whether they originate from the catalyst structure or develop under reaction conditions. In practice, such distinctions remain challenging, because these influences are often interdependent, dynamic, and difficult to resolve experimentally.

The hierarchical description of local environments also offers a useful parallel to the electric double-layer picture of the electrochemical interface.^52,69^ Just as primary, secondary, and extended environments distinguish different levels of interaction around the metal center, the electric double-layer picture distinguishes regions of interfacial organization involving solvent, ions, hydrogen-bonding networks, charge, and electric fields. Although the respective levels are not directly equivalent, both descriptions recognize that spatial proximity alone does not determine catalytic influence and that organization on different scales can affect adsorption, charge transfer, proton transport, and reaction kinetics. More generally, the relative importance of primary, secondary, and extended contributions depends on the catalyst, reaction, and operating conditions and may change as the local environment evolves during catalysis. Catalytic behavior therefore emerges from the interplay between site-specific local structure and broader catalyst- or condition-dependent influences across multiple levels of catalytic organization.

### 2.3 Structural and Electronic Descriptors of Local Environments

Having defined the local environment as a hierarchically organized system spanning primary, secondary, and extended influences, a corresponding set of descriptors is required to capture how the geometric organization and electronic character vary across these different levels. These descriptors provide the language through which local environments can be described, compared, and ultimately related to the catalytic behavior.

Structural descriptors capture the geometric and chemical organizations of the environment. Within the primary coordination sphere, these include the coordination number, geometry, bond lengths, and ligand identity.^70–75^ Even within this immediate coordination shell, significant heterogeneity may arise through mixed ligation, distorted geometries, or dynamic bond formation or cleavage, resulting in multiple closely related primary-sphere configurations. Moving outward, secondary environment descriptors capture how proximal modifiers are chemically identified, spatially arranged, and electronically or sterically coupled to the primary coordination sphere. Descriptors of the extended environment capture broader support, medium, and interfacial properties that shape the effective local environment, including features of the host matrix, the chemical environment encountered during synthesis, storage, activation, or reaction, and the surrounding gas, liquid, and electrochemical interfaces.

Electronic descriptors describe how this structural landscape translates into catalytic properties.^76,77^ These include oxidation state, partial charge, spin state, local density of states, charge transfer, and polarization fields in the surrounding matrix or interface.^19^ Crucially, these quantities should not be interpreted as intrinsic properties of an isolated metal atom but as emergent characteristics of the metal center and its surrounding environment. Structural and electronic descriptors are, therefore, deeply coupled. Even subtle variations in ligand identity, bond distances, or coordination geometry can strongly alter the electronic structure, while changes in oxidation or spin state may, in turn, induce structural rearrangements.

Catalytic environments, therefore, cannot be uniquely defined by a single descriptor. Instead, they are better represented by multidimensional descriptor sets that collectively capture geometric structure, electronic response, and environmental interactions across the primary, secondary, and extended levels (Figure 6). Some descriptors can be probed experimentally through spectroscopic or microscopic techniques, often in an ensemble-averaged manner, whereas others must be inferred from theoretical calculations or data-driven models, highlighting the need for integrated experimental and computational approaches (see below). Conventionally, in SACs, descriptors are often introduced as fixed properties of a site. In the local environment context, they are better viewed as context-dependent attributes that reflect the interaction among the single atom, the support, and condition-dependent species or fields. Their interpretation therefore depends on the environment in which they are defined, providing a basis for connecting the atomistic structure to catalytic behavior.

**Figure 6:**
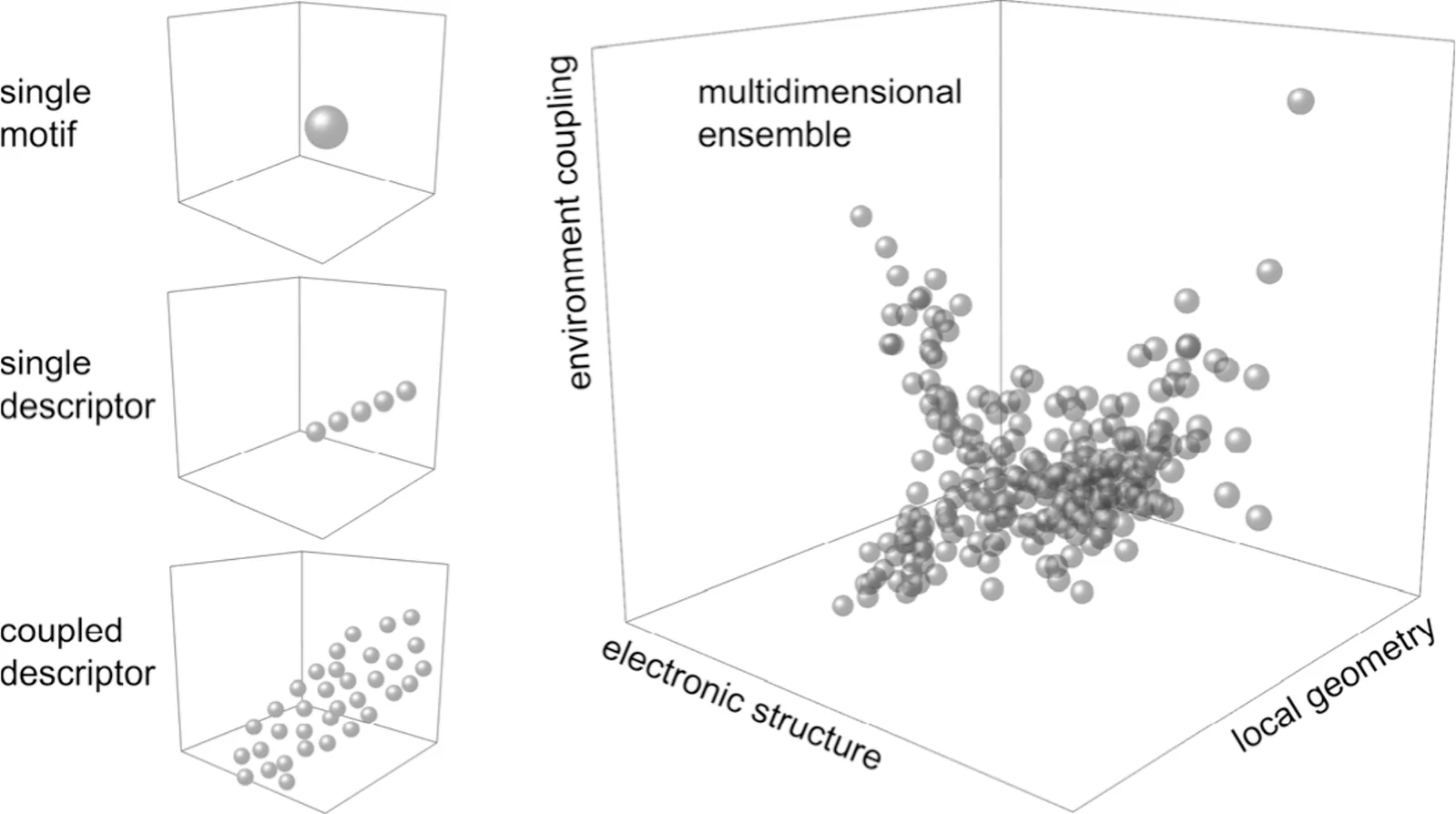
Representations of SAC local environments with increasing dimensionality, from a single structural motif to single descriptors, coupled descriptors, and multidimensional spaces capturing local geometry, electronic structure, and environmental coupling.

### 2.4 Dynamic and Metastable Local Environments

Local environments in SACs are inherently dynamic, evolving through coupled structural and electronic degrees of freedom.^46,78–80^ As introduced in Section 2.2, this behavior reflects the interplay between site-specific coordination structures, the surrounding support, and condition-dependent influences that emerge during activation and operation. Unlike molecular complexes with discrete ligands, supported single atoms are embedded in extended solids that can stabilize isolated metal centers while accommodating structural and electronic rearrangements without immediate metal dissociation.^81–83^ At the same time, reactants, intermediates, solvents, electrolytes, hydroxyl groups, or other interfacial species can act as transient ligands; while support atoms can reorganize, change oxidation state, or migrate. Subtle charge redistribution, lattice relaxation, or interfacial reorganization therefore expands the range of accessible local environments. Consequently, catalytic local environments are more appropriately viewed as dynamic ensembles rather than as single, static structures. Their evolution spans coupled time and length scales, each revealing complementary aspects of local environment dynamics (Figure 7).

**Figure 7:**
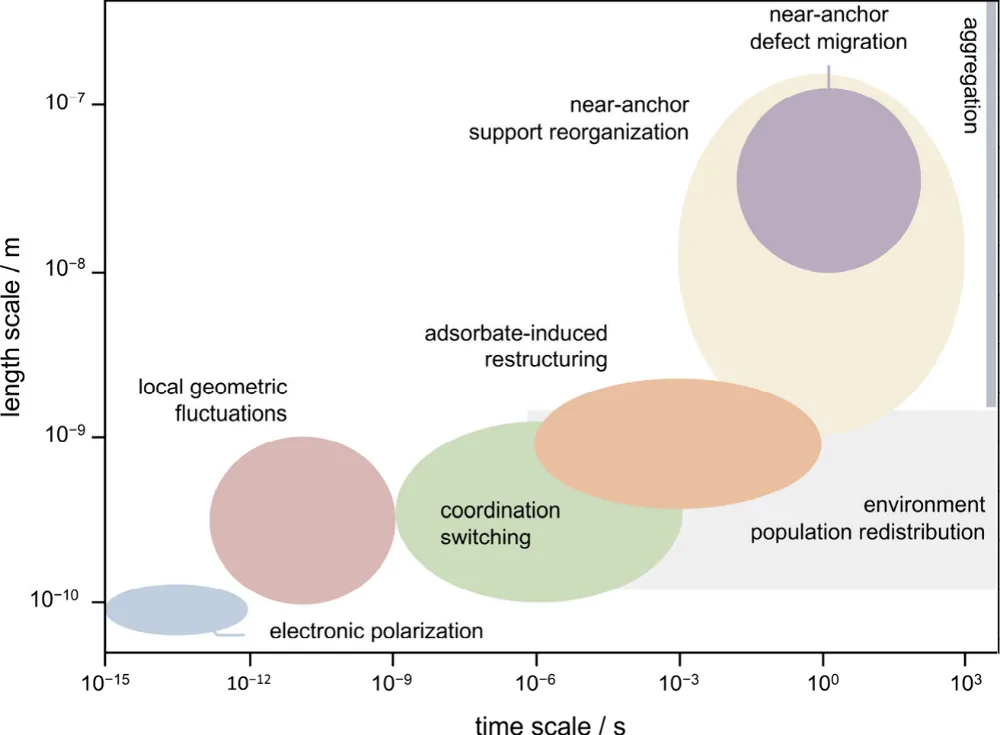
Time and length scales of dynamic processes governing local environments in SACs, spanning electronic and geometric fluctuations, coordination changes, and structural reorganization, and extending to ensemble redistribution and aggregation.

The dominant drivers of this evolution depend on the catalytic regimes. Thermal, electrochemical, and photochemical systems differ in whether changes in coverage, redox state, interfacial fields, electrolyte organization, illumination, or charge separation most strongly perturb the local environment. In all cases, these influences are coupled: operating conditions can modify the support, while the support determines how adsorbates, ions, charges, and interfacial species are stabilized and transmitted to the isolated metal center.

At the shortest time scales (femtoseconds to picoseconds), evolution is confined to continuous fluctuations of an existing local environment. Electronic polarization and thermal vibrations continuously perturb the electronic structure and interatomic distances while preserving the overall identity of the local environment. These intrinsic fluctuations constitute the dynamic background upon which all of the slower processes occur. Because these processes occur at time scales that are generally inaccessible to conventional operando techniques, they are primarily inferred from atomistic simulations and, where possible, complemented by ultrafast spectroscopic measurements in model systems.^84,85^

At intermediate time scales, typically spanning nanoseconds to milliseconds, although strongly system-dependent, local environments may undergo reversible structural adaptation. These changes can involve rearrangement of neighboring heteroatoms, adsorbates, hydroxyl groups, solvent molecules, electrolyte species, or support atoms, altering bond lengths, coordination numbers, ligation patterns, oxidation states, adsorption geometries, or the nearby support structure. Charge redistribution may also extend through the surrounding support or interface, coupling local coordination changes to broader electronic responses.^86^ When these processes remain reversible, the isolated metal site can retain its identity while it samples related configurations. Such adaptations can be probed by operando spectroscopies and complementary theoretical modeling, although their assignment often remains ensemble averaged and model dependent.^87^

Over longer time scales or larger observation windows, repeated sampling of accessible local environments can reshape their statistical populations, shifting the relevant description from individual configurations to the ensembles they form. Because multiple coordination configurations may coexist and interconvert, catalytically relevant structures often correspond to metastable states that are stabilized under specific reaction conditions rather than to a single global minimum structure.^88,89^ Changes in temperature, pressure, chemical potential, illumination, or electrochemical potential further redistribute the populations of these accessible environments, leading to variations in activity or selectivity, even when the nominal catalyst composition remains unchanged.^45,52,90^

Under prolonged operation, this redistribution may progressively lose reversibility as certain local environments become kinetically stabilized, whereas others are depleted. Accumulation of these changes can reshape the accessible landscape through support reconstruction, defect migration, atom migration, aggregation, metal atom loss, poisoning, and permanent changes in anchoring motifs. These longer-term transformations are typically resolved through long-duration operando experiments, time-resolved microscopy, and postreaction characterization.^91^ These temporal regimes provide a general framework for interpreting how SAC local environments evolve across experimentally accessible time scales. Although the specific time scales, structural adaptations, and dominant processes depend on the catalyst, reaction, and operating conditions, this temporal perspective provides a common basis for comparing local-environment dynamics across diverse SAC systems.

### 2.5 Local Environments Across the Lifecycle

Within this continuous evolution, synthesis, activation, operation, and eventual deactivation or regeneration represent successive stages through which the distribution of local environments is progressively established, modified, and, in some cases, restored (Figure 3). At any stage, the observed catalytic behavior reflects the cumulative outcome of this history rather than that of a single predefined structure.

The initial site populations are established during synthesis, where the choice of metal precursors, supports (or support precursors), and preparation conditions determines how metal atoms are anchored and which coordination motifs are initially established. Synthetic parameters such as temperature, atmosphere, and precursor chemistry therefore strongly influence the coordination environment landscape in the catalyst.^27^ Subsequent activation treatments, including thermal, reductive, or oxidative processes, often modify these initial environments. Ligands may be removed, coordination with the support may be reorganized, and the oxidation state of the metal center may change. These transformations frequently generate environments that become catalytically relevant under reaction conditions.

During operation, the population of environments is further redistributed through interactions with reactants, intermediates, solvents, and electrolyte species.^34^ These interactions can selectively stabilize certain coordination motifs while destabilizing others, shifting the distribution of environments present on the active surface. As a result, environmental populations may either converge toward a dominant configuration or diverge into multiple related structures. For example, a distribution of environments formed during synthesis or activation may preferentially reorganize into a common structure under reaction conditions,^92^ whereas interactions with adsorbates or poisons may lead to the emergence of additional configurations (Figure 8).^93^ Importantly, many of these structural adjustments remain reversible within the stability domain of isolated atoms.

**Figure 8:**
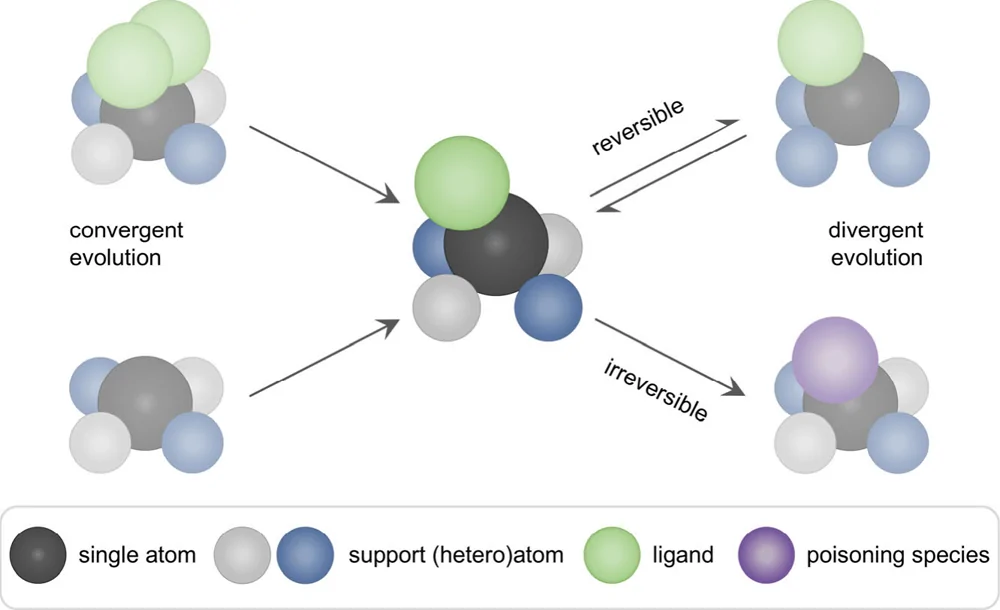
Evolution pathways of local environments during the lifecycle of SACs, showing transitions between distinct coordination environments, including reversible restructuring and irreversible transformations.

Over longer time scales, however, structural transformations may occur that exceed the stability limits of single-atom environments.^94,95^ Processes such as sintering, leaching, support degradation, or irreversible poisoning can alter the distribution of environments and ultimately lead to the loss of atomic dispersion. In this sense, deactivation can be understood as the point at which structural evolution drives the system beyond the regime in which isolated atoms remain stable. Regeneration treatments aim to reverse some of these transformations by restoring catalytically favorable environments.^96^ Although regeneration may not fully recover the initial structure, it can often reestablish coordination motifs that sustain the catalytic activity. This lifecycle perspective emphasizes that catalytic performance reflects not only the structure present after synthesis but also the sequence of environments formed, selected, and transformed during activation and operation.

### 2.6 Parallels to Homogeneous and Enzymatic Catalysis

Many of the ideas introduced in this section, environmental ensembles, secondary-sphere effects, adaptive coordination, and dynamic restructuring, are well-established in homogeneous and enzymatic catalysis.^97^ In these fields, reactivity has long been understood as an emergent property of a molecular environment rather than of a single metal center. Ligand fields, solvent shells, hydrogen bonding networks, and cooperative residues are recognized as integral components of the active site, shaping reaction pathways through subtle yet powerful interactions.

SACs extend this logic into a heterogeneous setting. Like molecular catalysts, SACs rely on coordination chemistry to define the electronic structure of the active center. The atoms forming the primary coordination sphere determine the immediate ligand field of the metal center, while nearby support atoms, functional groups, or locally organized interfacial species may act analogously to secondary-sphere elements in molecular or enzymatic systems. In both cases, catalysis emerges from the active center and surrounding structural or interfacial features that can adapt to the reaction coordinate.

At the same time, SAC environments differ fundamentally from those of molecular and biological catalysts.^98^ Their coordination environments are embedded within extended solids that impose mechanical constraints, electronic coupling, and collective responses with no direct analogue in solution-phase catalysis. This solid-state embedding confers intrinsic advantages, including enhanced mechanical robustness, higher thermal stability, and the ability to recover and reuse catalysts by using established heterogeneous catalysis protocols.

At longer length scales, the surrounding support introduces additional degrees of freedom that influence the catalytic behavior. Long-range charge redistribution, lattice strain, confinement effects, and support reducibility can modify the energetics of single-atom sites and couple the local coordination environments to the properties of the surrounding material. Conversely, the solid nature of SACs also introduces constraints.^28^ Mass and charge transport, site accessibility, and confinement within porous structures may influence catalytic performance independently of local coordination.

These parallels and distinctions are summarized in Table 1, which compares the roles of active centers, coordination spheres, and surrounding environments across homogeneous, enzymatic, and single-atom catalysis. The comparison should not be read as a set of direct equivalences but as a guide to which concepts have begun to translate into SAC design and which remain mainly heuristic. Secondary-sphere regulation, adaptive coordination, and confinement have already informed selected SAC studies, for example through heteroatom- and defect-defined environments that can tune charge density and spin state, activated carbon (AC) environments that regulate substrate availability in acetylene hydrochlorination, and metal-oxide interfaces that cooperate in activating hydrogen and transfer hydrogenation in carbon dioxide hydrogenation (see Sections 4 and 5 for further details). By contrast, enzyme-like allosteric regulation, long-range conformational control, and fully defined catalytic pockets have clear heuristic value but remain less established with comparable precision or predictability in SACs. Viewed through this lens, SACs occupy a unique conceptual position at the interface between molecular and heterogeneous catalysis: they provide access to coordination-level design concepts, but within extended, heterogeneous, and often distributional environments. This comparison can therefore help build a common language across catalytic fields, provided that transferable design principles are distinguished from concepts that currently serve mainly as an inspiration.

**Table 1:** Conceptual Parallels Across Homogeneous, Enzymatic, and Single-Atom Catalysis

concept	homogeneous	enzymatic	SAC	status in SAC design
active center	metal center	catalytic residue/cofactor	isolated metal atom	established starting point
primary coordination sphere	coordinating ligands	first-shell residues	anchoring motif	well established
secondary coordination sphere	ligand substituents	second-shell residues	proximal support atoms	increasingly demonstrated
extended environment	solvent/counterions	protein pocket	support/interfacial environment	partly demonstrated, system dependent
adaptive coordination	ligand exchange	conformational dynamics	coordination switching	demonstrated in selected systems
dynamic states	catalyst speciation	conformational ensemble	environment ensemble	important, difficult to characterize
long-range electronic coupling	limited	protein scaffold effects	lattice coupling	established starting point
transport environment	diffusion in solution	substrate channeling	pore diffusion/site accessibility	well established

## 3 Characterization of Local Environments

Understanding the catalytic behavior of SACs requires the ability to observe and quantify the local environments surrounding isolated metal centers. As established in Section 2, these environments span primary, secondary, and extended levels and may include both catalyst-derived and condition-dependent contributions. Their characterization therefore requires complementary approaches capable of probing both atomic structure and electronic properties across multiple length and time scales.

Over the past decade, advances in microscopy, spectroscopy, and computational modeling have greatly expanded our ability to characterize atomically dispersed catalysts. Numerous reviews have discussed these techniques in detail and established the methodological foundations of single-atom catalysis.^33,34,99,100^ Rather than surveying this extensive literature, this section focuses on the information these approaches provide about different levels of local environments, the extent to which they resolve their distributions, and the limitations that remain in linking experimental observables to specific catalytic structures. Accordingly, the discussion progresses through the complementary dimensions required to describe catalytic local environments, moving from structural characterization (Section 3.1) to electronic properties (Section 3.2), dynamic behavior under reaction conditions (Section 3.3), and finally, the integration of experimental observations with theoretical models (Section 3.4).

### 3.1 Local Atomic Structure and Site Geometry

Structural characterization of SACs relies on techniques that probe three complementary aspects: where isolated metal atoms are located, how their primary coordination spheres are structured, and which surrounding structural features can be associated with their secondary and extended environments. Resolving local environment distributions therefore requires combining local and ensemble-averaged structural information. Early structural studies focused primarily on confirming atomic dispersion, an advance made possible by the development of AC-STEM.^101,102^ High-angle annular dark-field scanning transmission electron microscopy (HAADF-STEM) has become a cornerstone technique because the strong *Z*-contrast allows isolated heavy atoms to be directly visualized against lighter supports such as carbon-based materials.^103–105^ As a result, imaging methods now routinely establish the presence and spatial distribution of isolated metal atoms on oxides, carbons, and other host materials. Beyond establishing dispersion and nuclearity, atomic-resolution imaging can place individual metal atoms within their structural context, relating their position, where identifiable, to surface or subsurface sites, crystallographic features, defects, and interfaces that may form part of the secondary environment. At a broader scale, simultaneous high-resolution imaging in high-angle annular dark-field and bright-field modes can map single atoms onto crystalline support lattices and distinguish their occurrence across locally ordered and disordered regions, thereby relating the metal site to the broader structural organization of the host.^106^ The depth of local environment information accessible from such images, however, is inherently contrast-dependent. While the metal center itself can often be readily localized, the light atoms defining its primary environment are generally much less accessible. Secondary and extended structural features can be resolved when they provide sufficient structural or chemical contrast, and their spatial relationship with the metal site can be established; however, surface/subsurface location, chemically similar heavy species, and projection effects remain important sources of ambiguity.^107^

Atomic-resolution imaging provides a highly local view of a catalyst, whose local environments are distributed across the material. Microscopy therefore captures individual realizations of this distribution within selected regions rather than directly representing the full catalyst population.^108^ In supported powders, metal-site context may vary with crystallographic orientation, facet exposure, defect structure, and support morphology. Imaging strategies must therefore use broader and more systematic sampling to capture the diversity and relative populations of the structurally distinct local environments.

Recent developments are beginning to extend the capabilities of electron microscopy beyond the qualitative identification of single atoms toward quantitative descriptions of SAC structures. Automated image-analysis workflows and machine learning (ML)-assisted atom detection have enabled the statistical analysis of large TEM data sets, tens of thousands of atomic positions,^109^ allowing metrics such as site density, intersite distances, clustering tendencies, and dispersion uniformity to be quantified (Figure 9a).^110^ Such a population-level analysis can also reveal relationships with the extended environment that are difficult to capture from individual sites. For example, spatial statistics can relate metal site distributions to the broader organization of anchoring sites or interactions imposed by the support, thereby constraining how the extended environment influences metal site organization beyond local coordination. As image analysis becomes increasingly quantitative, larger data sets improve statistical sampling but do not by themselves ensure a representative description of local-environment distributions. The challenge therefore shifts toward the reliability of the analytical workflow, its transferability across imaging conditions and catalyst structures, and its sensitivity to rare or structurally distinct environments. Image-derived descriptors must also account for the projection of three-dimensional materials into two dimensions: morphology, facet orientation, pore architecture, sample thickness, and through-thickness heterogeneity can alter the apparent relationships between the metal site and its surroundings.^111,112^

**Figure 9:**
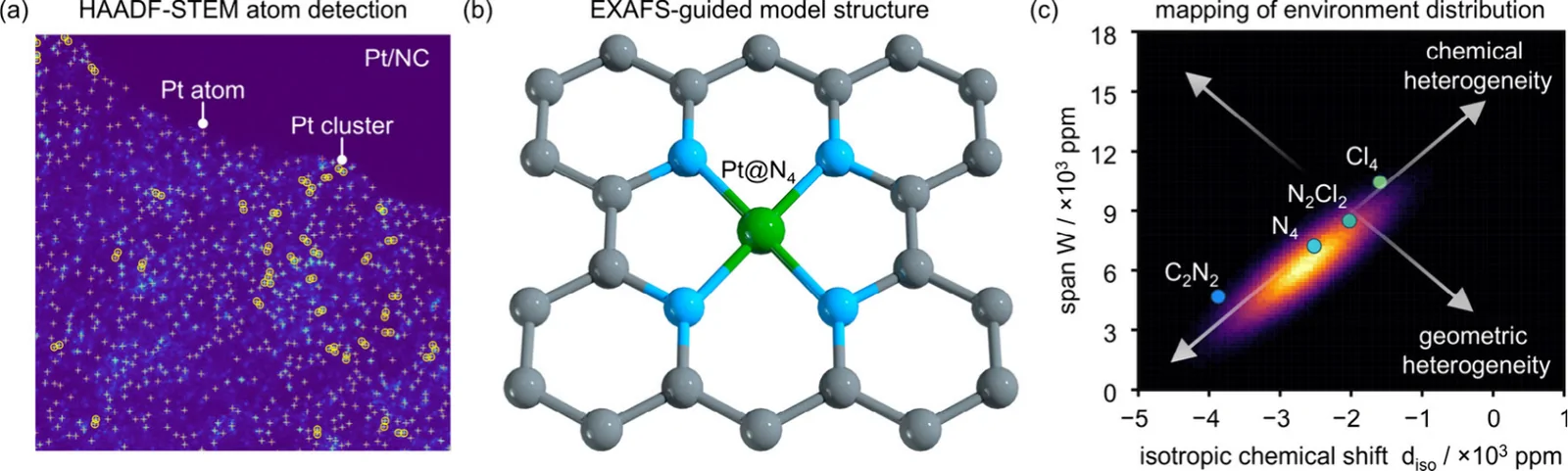
(a) HAADF-STEM imaging enabling direct identification of isolated Ptplatinum atoms and small clusters on Pt/NC. (b) Representative Pt@N_4_ coordination structure commonly used as a model for interpreting local environments. (c) ^195^Pt NMR mapping of local environment distributions on Pt_1_/NC. Part (a) adapted from ref 109 and part (c) adapted from ref 55, licensed under CC BY.

While HAADF-STEM directly confirms atomic dispersion and spatial distributions, it provides limited information on the identity and bonding of coordinating atoms, which often requires complementary spectroscopic probes.^113^ To gain further insights, it is regularly combined with spectroscopic capabilities such as scanning transmission electron microscopy energy-dispersive X-ray spectroscopy (STEM-EDX)^114^ or electron energy loss spectroscopy (EELS).^115^ STEM-EDX enables elemental identification and mapping around individual metal sites, whereas EELS can additionally provide, under favorable conditions, information on local oxidation state, electronic structure, and bonding. This combination can therefore move beyond locating the metal to provide chemical and spatial information on features associated with its primary and secondary environments while mapping compositional heterogeneity across the surrounding host relevant to the extended environment. Together with advances in sample preparation and imaging conditions, these developments have significantly improved the reliability with which isolated atoms can be identified and distinguished from small clusters or nanoparticles.

Complementing this projected structural and chemical information, scanning probe techniques provide direct atomic-scale information about catalyst surfaces, enabling simultaneous imaging of structural and electronic features. In particular, the combination of scanning tunneling microscopy (STM) and atomic force microscopy (AFM) probes individual metal atoms together with their surrounding surface structure, allowing specific site occupations and spatial relationships with lattice atoms, defects, vacancies, or adsorbates to be established without the depth superposition inherent to projected STEM images; STM additionally provides a local electronic contrast. This surface-specific view can therefore resolve structural relationships within accessible primary and secondary environments that are difficult to assign from projected images alone. For example, oxygen vacancies on oxide supports can be identified by STM and AFM with distinct signatures, and single metal atoms have been directly observed to occupy these defect sites.^116^

At the same time, imaging alone rarely resolves the full coordination environment that defines the catalytic function. The detailed structure of the primary coordination sphere is therefore commonly determined by using spectroscopic probes sensitive to short-range order. XAS, particularly extended X-ray absorption fine structure (EXAFS) analysis, has played a central role in determining coordination numbers, bond distances, and ligand identities in SACs.^117^ Beyond the primary coordination sphere, second-shell scattering can provide evidence for metal–support connectivity or neighboring metal atoms, thereby constraining structural features that may form part of the secondary environment, while detectable longer-range metal–metal coordination can indicate clustering or nanoparticle formation. Conversely, the absence of such contributions is consistent with atomic dispersion, although these assignments depend on the signal quality, scattering contrast, and structural models considered. Where overlapping scattering contributions limit conventional Fourier-transformed EXAFS, wavelet-transform EXAFS (WT-EXAFS) and multiple-scattering analysis can improve discrimination among candidate local environments.^103,118,119^

More generally, EXAFS provides an ensemble-averaged description of the local coordination around the absorbing atom, with structural parameters obtained by fitting the experimental signal to candidate models (Figure 9b). The resulting coordination numbers, bond distances, and disorder parameters therefore represent average structural features across the probed absorbing centers rather than the structure of an individual site.^120^ EXAFS can thus provide structural guidance on the primary and, when sufficiently resolved, secondary environment but does not by itself establish a unique local structure. Complementary techniques such as pair distribution function analysis,^121^ neutron scattering,^122^ or magnetic resonance spectroscopies^123^ further contribute to resolving structural motifs and defect environments in the host matrix, providing complementary information about broader structural features of the secondary and extended environments.

Increasingly, these structural probes are combined with controlled model systems to disentangle the closely related environments. For example, studies using shape-controlled oxide supports have demonstrated that nominally similar SACs may contain distinct surface and subsurface metal environments depending on the crystallographic facet or synthesis conditions.^124,125^ Likewise, recent solid-state nuclear magnetic resonance (NMR) approaches have begun to provide direct spectroscopic fingerprints of metal primary coordination environments in supported catalysts, enabling the identification of specific metal–support geometries and the quantification of site distributions within complex materials (Figure 9c). In particular, advanced ^195^Pt solid-state NMR has demonstrated the ability to map the local environments of isolated platinum centers by resolving both electronic and geometric contributions to the chemical shift tensor.^55^ This approach allows different coordination motifs, such as Pt@N_4_, Pt@N_2_Cl_2_, or mixed-ligand environments, to be distinguished spectroscopically. Such measurements provide one of the first direct experimental mappings of the distribution of local environments in SACs, offering insights that go beyond the ensemble-averaged structural information obtained from techniques such as EXAFS.

Despite these advances, structural characterization of SACs remains inherently challenging. In particular, structural assignment of the secondary environment may require complementary chemical or electronic information to establish which surrounding features are specifically associated with the metal center. For example, EXAFS can establish the average primary coordination environment, while XPS can identify relevant chemical species in the support. Combined with XANES and DFT, these constraints can narrow the range of structures consistent with the experimental observations, supporting assignment of secondary environments such as M@N_4_–C_*x*_O_*y*_.^126^ Extended structural features pose a different challenge, as their influence is often established by comparing catalysts with variations in broader host properties, such as morphology, crystallinity, porosity, strain, or structural disorder, rather than by direct site-specific assignment. Overall, structural characterization increasingly aims not only to assign a representative motif, but also to assess the distribution of environments that may contribute to catalytic behavior.

### 3.2 Electronic and Chemical State

The electronic structure of SACs emerges from the coupled interaction between the metal center and its surrounding environment. Direct metal–ligand coordination within the primary environment defines the ligand field experienced by the metal center, while defects, dopants, or heteroatoms within the secondary or extended environment can influence local electrostatics and charge polarization (Figure 10). Broader electronic properties of the support, including charge-transfer and hybridization effects, can further influence the metal center through the extended environment. Because no single experimental observable captures these interactions in full, characterization of electronic local environments relies on complementary spectroscopic techniques that probe different aspects of the electronic structure.

**Figure 10:**
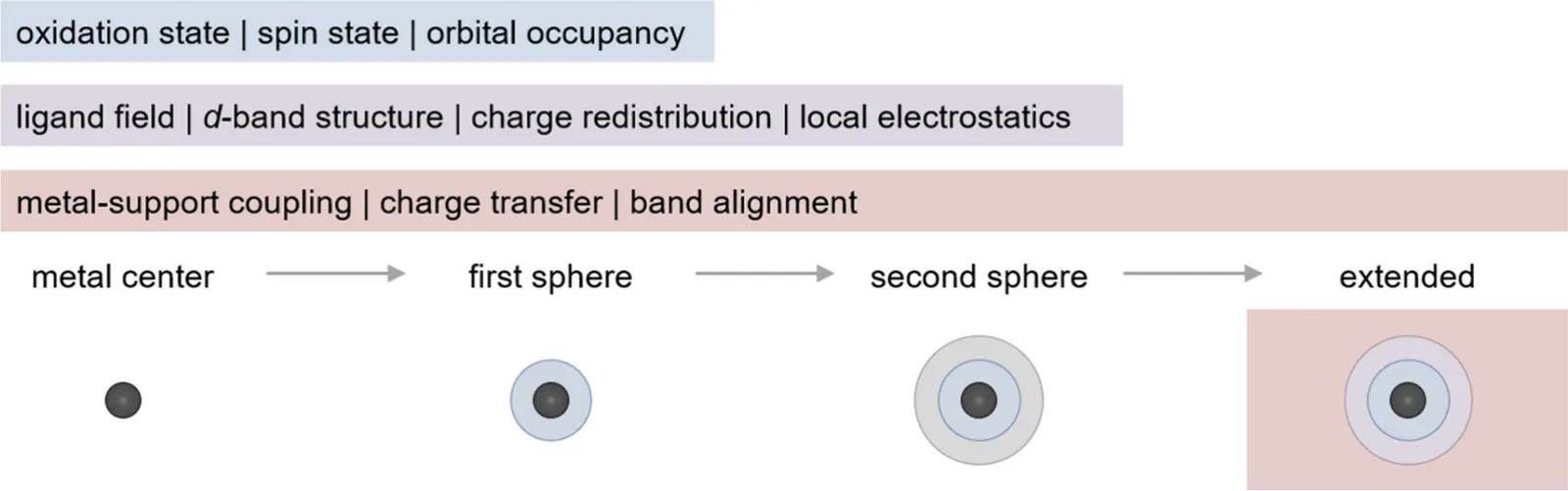
Evolution of electronic descriptors across coordination spheres, from the metal center through the near environment to the extended support, reflecting the progressive influence of the surrounding environment.

Electronic characterization commonly begins by determining how the local environment redistributes the electron density around the metal center. X-ray photoelectron spectroscopy (XPS) binding-energy shifts probe changes in the electrostatic potential surrounding the absorbing atom, X-ray absorption near-edge structure (XANES) positions reflect changes in the population and energy of unoccupied electronic states, and spatially resolved EELS maps local variations in electronic structure with near-atomic spatial resolution in AC-STEM.^103,127^ These measurements show that reducible oxides, doped carbons, or vacancy-rich materials can act as electron reservoirs, modulating the electronic structure of the metal center and influencing reactivity. The resulting redistribution of electron density can be further quantified computationally through partial-charge descriptors, whose values depend on the adopted charge partitioning scheme.^85^ Although charge-sensitive techniques reveal how electron density is redistributed, they provide only limited information about how the electronic states themselves are organized. Characterizing the electronic configuration therefore requires probes that are sensitive to orbital energies, orbital occupancies, and spin states.^128^ This information is particularly important for first-row transition metals, where ligand-field effects can generate multiple electronic configurations.^77^

Mössbauer spectroscopy has been widely used to distinguish among different iron coordination motifs and spin states in SACs with Fe@N_*x*_ environments,^129,130^ while EPR provides complementary information on paramagnetic centers, local symmetry, and interactions with defects or adsorbates.^131^ As an illustrative example, in Mn_1_/CeO_2_, HAADF-STEM imaging reveals highly ordered nanocrystals and elemental mapping confirms a high dispersion of manganese (1 wt %), yet it cannot be directly visualized due to the limited contrast with the support (Figure 11a).^132^ In this case, EPR plays a decisive role by identifying magnetically isolated Mn^2+^ species through their characteristic hyperfine splitting (Figure 11b). The presence of these low valent species reflects strong metal–support interactions, where manganese promotes the formation of oxygen vacancies and modifies the redox state of the ceria lattice, as supported by Raman spectroscopy of the used catalyst (Figure 11c). As such, the observed electronic configuration cannot be attributed solely to the primary coordination geometry but instead emerges from the coupled interaction between the metal center and the surrounding support across different levels of the local environment. Advanced X-ray spectroscopies such as X-ray emission spectroscopy (XES) and resonant inelastic X-ray scattering (RIXS) can provide complementary access to *d*-orbital occupancy, ligand-field splitting, spin state, and metal–ligand covalency by resolving features such as *d*-*d* excitations and multiplet structure.^133^

**Figure 11:**
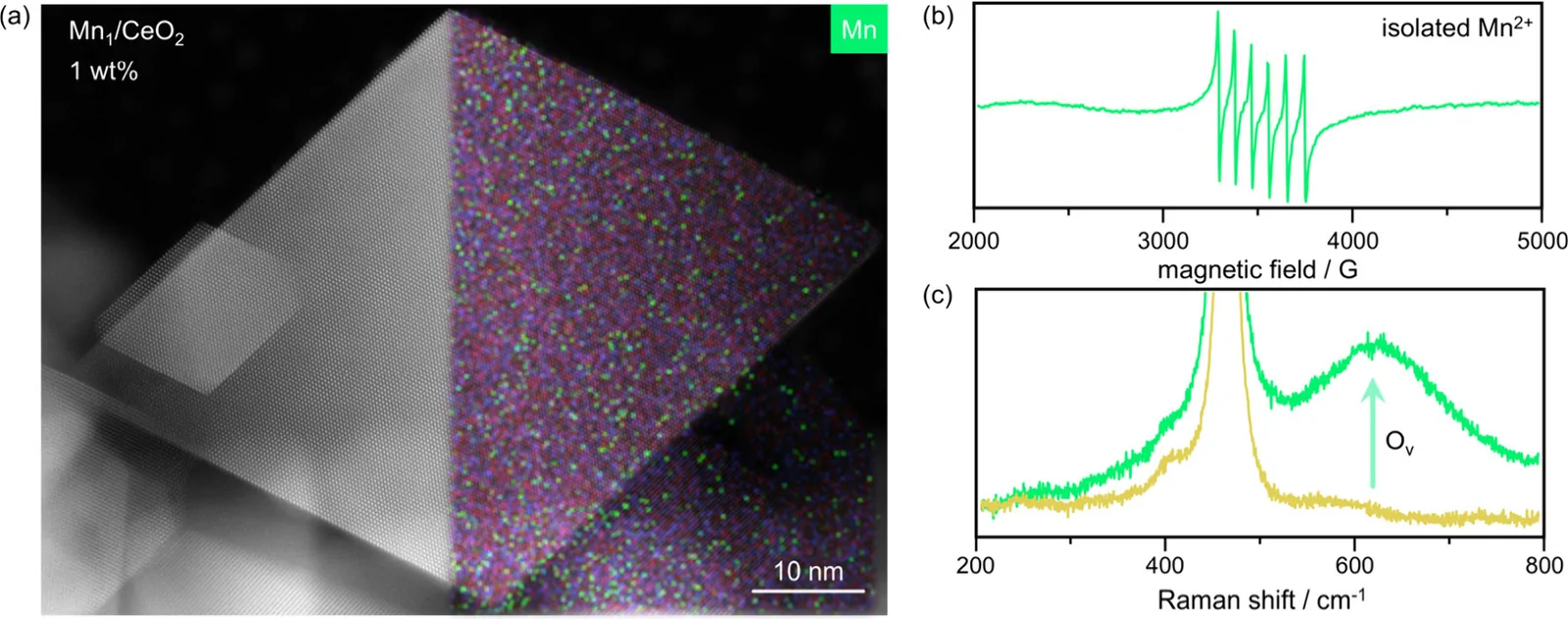
(a) HAADF-STEM image of Mn_1_/CeO_2_ with EDX maps partially overlaid. (b) EPR spectra displaying the characteristic hyperfine splitting of isolated Mn^2+^ centers on CeO_2_. (c) Raman spectra showing the presence of oxygen vacancies in the ceria lattice. Adapted from ref 132, licensed under CC BY-NC.

The reconstructed electronic structure is often distilled into the chemically intuitive descriptor of formal oxidation state, an inferred electron-counting convention whose assignment relies on the combined interpretation of spectroscopic observables such as XANES and XPS.^134–136^ Within this framework, isolated atoms on oxide supports frequently exhibit signatures consistent with cationic metal centers associated with M@O_*x*_ coordination motifs,^137^ whereas coordination to nitrogen or other heteroatoms stabilizes M@N_*x*_ environments with characteristic electronic signatures on carbon supports.^138^ The formal oxidation state nevertheless remains the most widely reported electronic descriptor because it provides an intuitive framework for comparing active sites and describing oxidation–reduction processes.^139,140^ However, as an inferred chemical interpretation, it does not necessarily capture the full complexity of the underlying electronic structures. XPS analysis of support elements can provide complementary chemical state information by distinguishing chemically inequivalent species through their core-level binding energies. For example, changes in N 1*s* can identify nitrogen involved in metal coordination, while analogous signatures from nearby heteroatoms or functional groups can constrain features of the secondary environment when their site-specific relationship with the metal center is independently established.^141^

One of the main challenges in electronic characterization is that single-atom catalysts rarely exhibit a single, well-defined electronic configuration. Variations in the coordination environment, support composition, defect structures, or adsorbate binding can stabilize closely related electronic states within the same material. As a result, spectroscopic measurements frequently reflect ensemble averages over populations of sites with distinct electronic configurations. Resolving these electronic landscapes and linking them to catalytic performance require combining complementary methods to distinguish and assign distinct states and their populations. A further challenge is assigning the origin of these electronic differences to specific levels of the local environment. Whereas structural characterization can directly associate particular atoms or motifs with the primary or secondary environment, the electronic state measured at the metal center reflects the coupled influence of multiple environmental levels and cannot generally be partitioned among them. Secondary and extended electronic influences must therefore be inferred by integrating metal-centered electronic observables with structural and support-sensitive information.^142^

### 3.3 Dynamic and Working Environments

Characterizing dynamic and working environments requires determining both how the catalyst evolves during operation and how operating conditions generate or modify the features of the local environment. Operando spectroscopies have become particularly powerful tools for tracking these changes under the reaction conditions. Among the most widely applied approaches, operando XAS has provided direct evidence for reversible changes in coordination number, bond distances, and oxidation states during the reaction.^34^

An important advantage of operando spectroscopy is the possibility of disentangling how individual environmental factors influence the electronic state of a catalytic site. This is illustrated by a recent in situ XANES study of a Pd_1_/C_3_N_4_ SAC for the Suzuki coupling. Using a microfluidic cell to probe the catalyst under controlled liquid-phase conditions (Figure 12a), the authors observed systematic shifts in the Pd L-edge as the surrounding chemical environment was varied, including solvent, base, ligand, and full reaction mixture.^143^ The spectra remained characteristic of cationic palladium species, indicating that strong metal–support interactions stabilize the palladium center and prevent reduction to metallic Pd^0^ (Figure 12b). Instead, coordination by ligands and reagents modulates the electron density of the palladium site within the cationic state. By isolating the contribution of individual components, the study demonstrates how operando spectroscopy can resolve the environment-dependent electronic states of SACs. Complementary operando EPR enables direct tracking of paramagnetic metal centers under reaction conditions, resolving their reversible redox interconversion (e.g., M^2+^/M^+^) in real-time and quantifying the fraction of sites actively participating in catalytic cycles.^144^

**Figure 12:**
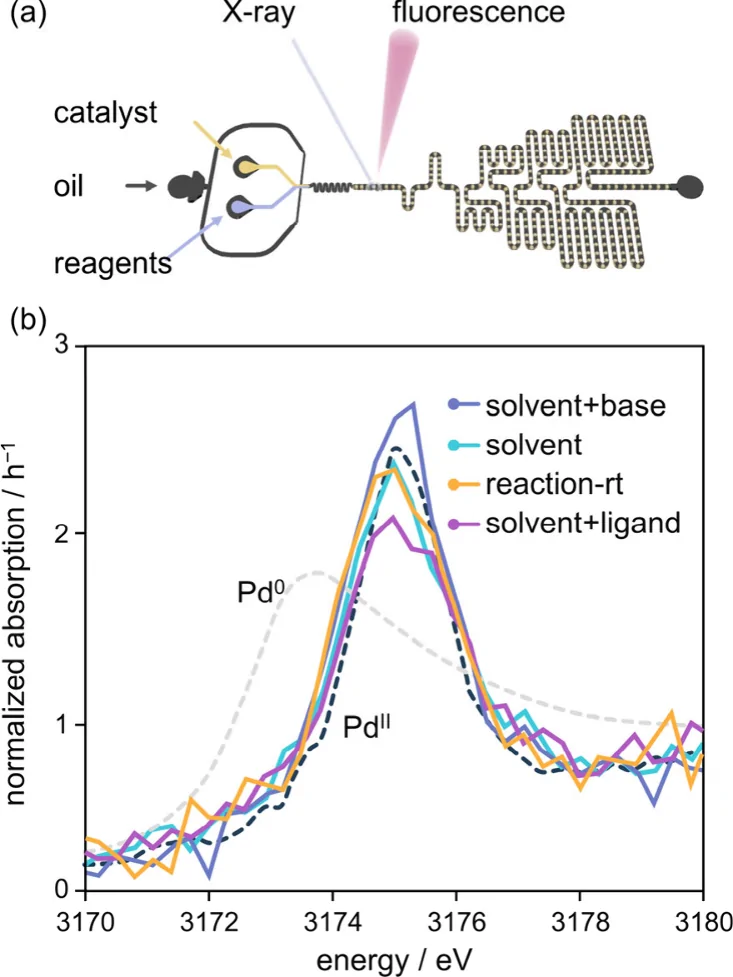
(a) Droplet-based microfluidic cell for operando studies of liquid-phase cross-coupling applications and (b) in situ XANES spectra showing shifts in the Pd L-edge position across different coordination environments while maintaining a cationic state. Adapted from ref 143, licensed under CC BY.

Operando measurements can also determine whether reaction-derived species become transient components of the local environment. For example, potential-resolved XAFS and synchrotron radiation IR spectroscopy showed that an initially Ir@N_*x*_ single-atom site dynamically incorporates an additional O ligand under OER conditions before the appearance of *OOH intermediates.^145^ The disappearance of the coupled O after the reaction demonstrated the reversible, potential-dependent nature of this coordination change. This condition-dependent ligation becomes part of the primary environment only within the relevant operating window, distinguishing the resting structure from the working environment and providing a basis for understanding the structural evolution discussed in Section 5.2.

Surface-sensitive spectroscopies provide complementary insight into electronic and chemical transformations occurring at the catalyst interface under reaction conditions. Near-ambient-pressure X-ray photoelectron spectroscopy (NAP-XPS) has enabled direct observation of oxidation-state changes, surface chemical states, and adsorbate interactions under controlled reactive gas atmospheres and elevated pressures, thereby reducing the pressure gap associated with conventional UHV measurements,^81,90^ while operando IR and Raman spectroscopies have identified adsorbed intermediates and ligand rearrangements associated with specific reaction pathways.^134,146,147^ These techniques track how the metal center, adsorbates, and surrounding interfaces evolve during catalysis.

Beyond the catalyst itself, operando characterization can probe condition-dependent features that develop at catalyst–electrolyte interfaces. Vibrational spectroscopy can provide information on site-associated solvation and interfacial organization by distinguishing water populations and orientations, hydrogen-bonding networks, and cation-associated water structures, while complementary electrochemical measurements can probe changes in ionic organization and interfacial electric fields. For example, potential-dependent measurements during alkaline HER have revealed changes in interfacial water organization and orientation and cation-induced restructuring of the interface. These approaches therefore provide access to condition-dependent secondary- and extended-environment contributions, although their ensemble-averaged nature generally limits direct assignment to individual metal centers.^148^

Another important direction is the emergence of in situ and operando electron microscopy, which enables structural evolution to be monitored under reactive or electrochemical environments.^34^ Although still technically challenging for many catalytic systems, environmental TEM studies have demonstrated the possibility of visualizing atomic-scale transformations and following the behavior of isolated metal atoms during catalytic processes or electrochemical cycling.^149–152^ These observations highlight the dynamic nature of single-atom environments and provide direct evidence that isolated atoms may migrate, reorganize, or interact with adsorbates during operation.

In gas-phase systems, environmental TEM can further monitor atomic-scale evolution under controlled reaction atmospheres, allowing changes in the local environment to be related to operating conditions and, when coupled with outlet gas analysis, to catalytic performance. Such correlations should nevertheless consider that the conditions experienced by the imaged region may differ from the nominal reaction conditions because of the specific measurement environment.^153^

The temporal hierarchy of local-environment dynamics introduced in Figure 7 also defines the experimental window of operando characterization. No single technique captures the full evolution of catalytic local environments; each selectively probes a particular range of temporal, spatial, and chemical sensitivity. Table 2 summarizes these complementary capabilities and their principal limitations for assigning working local environments. Time-resolved measurements further show that catalytic operation often involves redistribution among multiple site environments rather than a single dominant structure. Changes in the temperature, reactant concentration, or electrochemical potential can shift the relative populations of coordination motifs or electronic states within the catalyst. Techniques such as transient spectroscopy, modulation-excitation experiments, and rapid-scan XAS methods have begun to probe these processes, revealing kinetic coupling between catalyst restructuring and reaction turnover.^154,155^

**Table 2:** Representative Operando/In Situ Tools for Probing Dynamic Local Environments in SACs.

dynamic regime	example processes	suitable tools	main limitations
electronic polarization, charge redistribution	fast changes in charge density, oxidation state, metal–support electronic coupling	time-resolved XAS, ultrafast optical or X-ray spectroscopy, transient absorption, electrochemical spectroscopies	rarely accessible under realistic catalytic conditions; often inferred indirectly
local geometric fluctuations, coordination switching	changes in bond distances, ligand exchange, reversible coordination changes	XANES/EXAFS, rapid-scan XAS, EPR for paramagnetic states, Raman/IR for ligands/adsorbates	ensemble averaging; short-lived or minority states may be masked
adsorbate-induced restructuring	adsorbate binding, surface relaxation, vacancy-adjacent coordination changes	XAS, DRIFTS, Raman, AP-/NAP-XPS, EPR	difficult to separate spectator species; metal-site assignment may be indirect
population redistribution among local environments	shifts between site motifs, oxidation states, or coordination populations	XAS with linear-combination/multivariate analysis, EPR, (N)AP-XPS, modulation-excitation spectroscopy, transient kinetics	deconvolution is model-dependent; minority sites may be hidden by dominant spectators
defect migration, near-anchor support reorganization	oxygen vacancy formation/migration, hydroxylation, local lattice rearrangement	EPR, Raman, XAS, isotope experiments, PDF, gas/liquid (S)TEM	spatial heterogeneity, EPR-silent defects, beam/probe effects, limited P range
aggregation, sintering, deactivation, carbon deposition	atom migration, cluster formation, irreversible restructuring, coking	gas/liquid (S)TEM, XAS, AP-/NAP-XPS, Raman, ex situ analyses	sampling statistics; irreversible changes may occur during transfer or quenching

Despite these advances, operando measurements typically probe ensemble-averaged properties, such as coordination numbers, oxidation states, or adsorbate populations. While these measurements reveal how catalysts evolve toward their steady-state configuration, they rarely allow for direct discrimination between distinct coordination motifs within the catalyst. Instead, operando observations generally constrain the range of structures consistent with measured signals. Consequently, the absence of an observable change should not be interpreted as evidence that the active site is intrinsically static but rather that no change was detected within the temporal resolution, spatial averaging, population sensitivity, and chemical contrast of the selected technique. Resolving the distribution of environments that contribute to catalytic activity thus requires combining operando spectroscopy with complementary structural characterization and theoretical modeling.

### 3.4 Integrated Modeling and Data Fusion

Single-atom catalysis has become a central platform for experiment-theory integration, as catalytic performance is highly sensitive to the local environment of individual metal atoms, which can be directly interrogated through electronic structure simulations. Spectroscopic techniques provide information on coordination, oxidation states, and adsorbate interactions, yet the measured signals typically arise from the weighted contribution of multiple coexisting environments rather than from a single well-defined active site. Consequently, determining the underlying distribution of local environments from the experimental observables alone constitutes an inverse problem, as different structural ensembles can produce similar spectroscopic responses. Electronic structure simulations address this challenge by evaluating the properties of individual candidate environments, enabling the ensemble-averaged experimental response to be interpreted at the atomic scale. A more robust strategy is to construct multiple plausible candidate local environments based on chemical intuition, synthesis and operating conditions, and known coordination chemistry. Following geometry optimization, the predicted spectroscopic signatures of these candidates should be compared directly with the corresponding experimental observables, preferably using multiple independent measurements to progressively reject incompatible environments and constrain their plausible populations. The resulting experiment–theory agreement is then used to iteratively refine both the candidate structures and their relative populations until a chemically consistent structural ensemble is obtained that is compatible with the available spectroscopic evidence and, where relevant, the observed catalytic behavior (Figure 13). When several environments remain consistent with these constraints, they should be retained as plausible members of the ensemble rather than reduced to a unique structural assignment solely based on the best fit.

**Figure 13:**
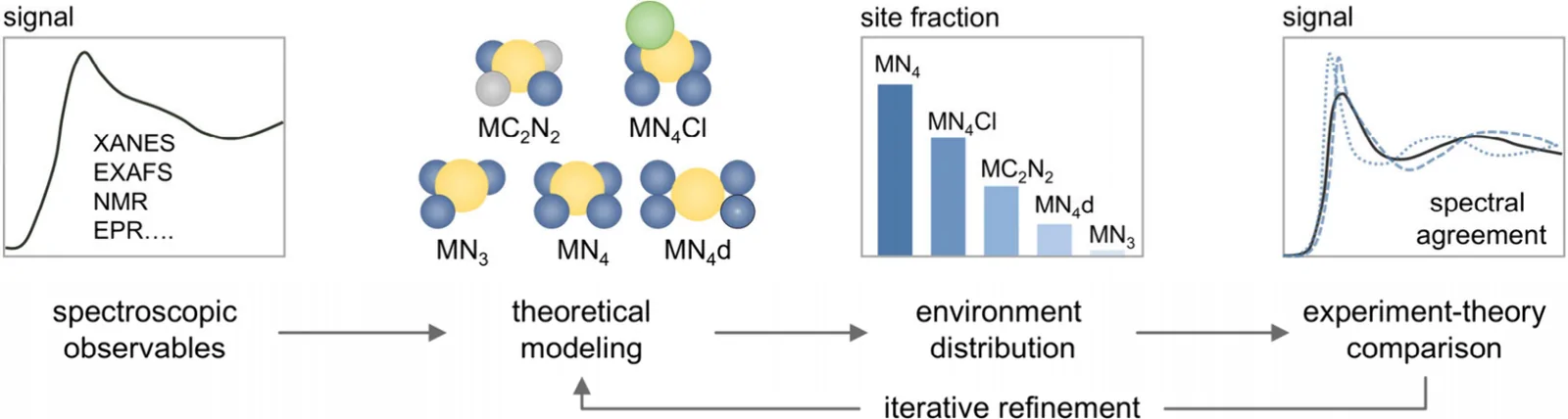
Experiment-theory integration for resolving local environments in SACs, linking spectroscopic observables with simulated coordination environments through iterative comparison.

Density functional theory (DFT) constitutes the central theoretical framework for this approach, offering a practical balance between accuracy and computational cost and enabling systematic exploration of local environments and adsorbate interactions, supporting the construction of structural models consistent with both spectroscopic data and catalytic trends.^156^ This includes the identification of coordination motifs and oxidation states, the evaluation of adsorption energetics, the elucidation of reaction mechanisms through activation barriers, and the assessment of catalyst stability, including metal–support interactions and resistance to aggregation. In addition, electronic structure analyses provide descriptors that rationalize trends in activity, selectivity, and stability.^157^ The relevant simulations should therefore be selected according to the experimental constraint being tested: structural models can be compared through predicted spectroscopic signatures, electronic descriptors through the corresponding electronic observables, and catalytic hypotheses through adsorption energies, reaction energetics, and kinetic predictions.

Constructing structural models that adequately represent the relevant catalytic environments remains one of the central challenges of electronic-structure modeling. In single-atom catalysis, this task is particularly system-dependent because different classes of supports give rise to different regimes of structural complexity and therefore require different modeling strategies.^158^ Carbon-based SACs, for example, typically span broad distributions of local environments shaped by defects, edge sites, surface functionalities, and precursor coordination chemistry, such that a single coordination motif is rarely representative of the material. By contrast, for SACs based on reducible oxides, the key challenge often lies in the interplay between metal coordination, charge transfer, lattice defects, and support redox dynamics, while on more ordered oxide or nitride supports, the relevant structural space may be more strongly governed by crystallographic terminations and well-defined defect sites. Because these environments can display markedly different energetics, the resulting spread in calculated properties can exceed the intrinsic uncertainty of the DFT. As a result, the accuracy and interpretability of modeling frameworks depend heavily on whether the chosen structural representation is appropriate for the system;^158^ candidate generation should therefore reflect the experimentally and chemically plausible heterogeneity of each catalyst rather than applying the same structural search space across different support classes.

To address this, modeling strategies increasingly move beyond single-site descriptions toward ensemble-based representations, in which families of local environments are considered, and their populations are determined by thermodynamic stability.^158^ Where possible, experimentally constrained populations should be considered alongside thermodynamic predictions, since stability alone does not establish the abundance or catalytic participation of a given environment. Crucially, these ensembles should not be treated as static, but as evolving across the catalyst lifecycle, from site formation during synthesis to structural adaptation under reaction conditions and, ultimately, to the pathways governing deactivation and regeneration. Within this framework, catalytic performance emerges from the collective contribution of multiple environments rather than from a single predefined active site, enabling a more direct comparison with ensemble-averaged experimental observables and a more transparent assessment of structural uncertainty. The structural scope of each individual model is equally important. Models centered on the primary coordination sphere can resolve metal–ligand interactions, but secondary features and extended support contributions often require progressively broader representations. Larger cluster or periodic models can incorporate proximal defects, heteroatoms, confinement, lattice relaxation, and longer-range interactions, while periodic descriptions are particularly relevant for extended properties such as band structure and charge delocalization. Primary, secondary, and extended contributions are therefore not always represented simultaneously, making the choice of model size and boundary conditions an important part of defining the local environment computationally.

A more fundamental challenge is that structural assignments are intrinsically nonunique, as multiple local environments may reproduce the available experimental evidence with comparable confidence. This ambiguity is difficult to quantify, because the complete space of chemically accessible environments is itself unknown. Neither the formation of SACs during synthesis nor their structural evolution under reaction conditions can yet be predicted exhaustively from first principles, making it impossible to demonstrate that all plausible structural candidates have been considered.^116^ Consequently, robust structural modeling relies not only on integrating complementary constraints, including independent spectroscopies, thermodynamic stability, catalytic performance, and chemical consistency, to progressively eliminate incompatible models and constrain the space of plausible solutions, but also on recognizing that the best-fitting structural model represents only one of several chemically plausible local environments consistent with the available evidence.

Beyond static DFT, kinetic and multiscale approaches are required to connect atomistic energetics with catalytic performance.^159^ In the context of SACs, microkinetic modeling plays a particularly critical role, as reactivity depends not only on intrinsic energy barriers but also on coverage effects, competing pathways, and the accessibility of intermediate states under operating conditions.^160^ These factors are especially important in SACs, where low coordination and strong adsorbate-site interactions can lead to complex reaction networks and condition-dependent activity. Accordingly, agreement between a candidate environment and structural characterization should be complemented, where possible, by testing whether its predicted behavior is compatible with experimentally observed activity, selectivity, and their dependence on the operating conditions.

Capturing the dynamic nature of the catalytic environments remains a key challenge. While static DFT provides snapshots of minimum-energy configurations, SACs often exhibit significant structural flexibility, including metal displacement, ligand rearrangement, and adsorbate-induced restructuring.^161^ Ab initio molecular dynamics (AIMD) can partially access these effects by capturing configurational fluctuations and entropic contributions.^162^ However, the accessible time and length scales remain limited, restricting the exploration of slower processes, such as diffusion, site interconversion, or support restructuring.

ML approaches provide a route to extend these capabilities.^9^ Machine learning interatomic potentials trained on DFT (MLIPs) data enable molecular dynamics (MD) simulations at near-first-principles accuracy while dramatically increasing accessible length and time scales.^163^ Longer trajectories provide broader sampling of configurational fluctuations, transitions between local states, and slower structural evolution, extending the dynamic description beyond the short-time scale fluctuations accessible to AIMD. Larger simulation cells, in turn, allow secondary interactions and broader support or interfacial contributions to be represented explicitly rather than truncated or treated separately. The combination of these spatial and temporal scales enables processes such as site mobility, restructuring, and adsorbate–support coupling to be examined within environments in which primary, secondary, and extended contributions coexist and evolve, providing a more complete representation of the working local environment. At the same time, these approaches remain dependent on the quality and representativeness of the training data, and their application to chemically complex, disordered supports is still emerging.

Modeling under reaction conditions further requires representation of the condition-dependent features that shape the local environment during operation. This may involve determining how the reaction atmosphere and adsorbate populations stabilize different local structures in gas-phase catalysis; incorporating solvent organization, ions, interfacial fields, and potential-dependent configurations around the metal site in electrocatalysis; or describing photoinduced carrier localization and charge transfer that modify the electronic environment of the site in photocatalysis. Theoretical approaches ranging from thermodynamic and statistical treatments to explicit interfacial and excited-state descriptions provide different levels of access to these effects, progressively bridging idealized structural models with the local environments established under working conditions.^164–166^ Overall, modeling of SACs is evolving toward integrated, multiscale descriptions that explicitly account for structural diversity, coverage effects, and dynamic behavior.^11^

## 4 Synthesis-Directed Local Environments

The formation of local environments in SACs is governed not only by the choice of precursor of the desired element or synthetic protocol but, more fundamentally, by the chemistry and structure of the catalytic components with which the isolated center interacts. In many SACs, the support plays the dominant role, simultaneously serving as a stabilizing matrix that anchors isolated atoms, a ligand scaffold that largely defines their immediate coordination environment, and an electronic partner that tunes the properties of the metal center. Additional components, including promoters, ligands, or other additives, can further modify or be integrated into these environments. Consequently, the coordination motifs that emerge, such as M@O_*x*_ motifs on oxides or M@N_*x*_ configurations in NC supports, are determined by the available anchoring sites of the support, defect structures, and other locally interacting catalysts.

While numerous reviews have summarized synthetic approaches, such as wet-chemistry routes, pyrolysis of molecular precursors, and vapor-phase deposition methods,^167,168^ and have emphasized the role of supports in determining SAC properties,^27,28,169^ these dimensions are often treated independently. From the perspective of catalytic environments, however, the synthetic strategy and catalyst composition must be considered together; the synthesis route determines how the targeted atoms access and are stabilized within defined motifs and how additional catalyst components become spatially and chemically associated with them.

Rather than providing a comprehensive survey, this section draws on representative examples to illustrate how support class and synthetic route codetermine the resulting local environments. We focus on oxide- (Section 4.1) and carbon-based (Section 4.2) SACs because they encompass a large fraction of the literature while representing two complementary regimes of local environment formation. In the former, local environments are predominantly governed by the intrinsic structure and chemistry of the host, whereas in the latter, they are more strongly influenced by synthetic design and chemical functionalization. Other classes are discussed more briefly because they reflect different combinations of these same governing principles rather than constituting distinct paradigms of local environment formation (Section 4.3).

We further explore how synthetic variables influence motif distribution and uniformity and how metal introduction methods govern site accessibility (Section 4.4). While the focus is on isolated metal centers, these principles extend to dual-atom catalysts (DACs)^170–173^ and geminal-atom catalysts (GACs),^174^ where higher metal densities enable new coordination modes and cooperative effects. These emerging systems offer unique opportunities to tailor local environments through controlled spatial arrangements, reinforcing the broader potential to engineer catalytic environments through the coordinated design of the active center, the support, promoters, and other catalyst components.

### 4.1 Oxide-Based Single-Atom Catalysts

Oxide supports were among the first materials on which isolated metal atoms were proposed as catalytically relevant species, most notably in the water–gas shift reaction^3^ and carbon monoxide oxidation.^25^ Their prominence in SACs stems not only from their extensive history as catalyst supports but also from their favorable physicochemical properties. They can be prepared in nanostructured forms with high surface areas, often possess tunable defect and acid–base chemistry, and offer strong metal–support interactions that stabilize isolated metal centers.^175^ In oxide-based SACs, metal atoms are typically anchored through coordination with lattice oxygen, binding at intrinsic or induced defect sites (such as oxygen vacancies or hydroxyl groups). The amount of metal that can be stabilized in single-atom form is governed by the density and nature of these anchoring sites, controlled by surface area and surface terminations, as well as by the thermodynamic balance between stabilization through metal–support interactions and aggregation into bulk metallic or oxidic phases.

On oxide supports, isolated metal atoms can occupy a range of structural positions relative to the lattice (Figure 14), each giving rise to distinct M@O_*x*_ coordination motifs that define their primary coordination sphere.^53,116,132,176^ The least integrated configuration corresponds to surface adatoms, where metal atoms bind to surface oxygen atoms without incorporation into the lattice. These species typically exhibit lower coordination numbers and may display significant mobility depending on the strength of the metal–support interaction and the availability of anchoring sites. Their coordination environment may be further stabilized by ligands such as surface hydroxyls or adsorbed molecules, which supplement coordination and modulate the metal’s electronic structure.

**Figure 14:**
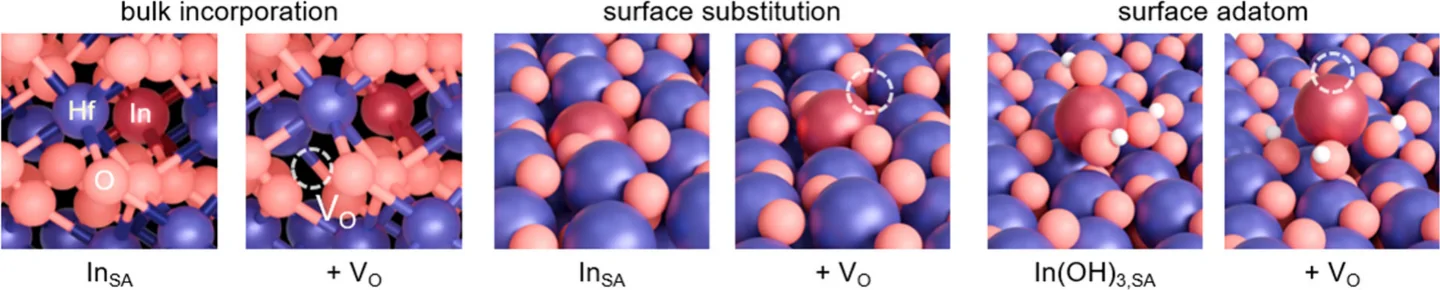
Possible configurations of isolated metal centers on oxide supports, illustrated for In_1_/HfO_2_, including bulk incorporation, surface substitution, and hydroxyl-stabilized adatoms, with site stabilization influenced by the presence of oxygen vacancies (V_O_, dashed circles). adapted from ref 176.

A second configuration involves surface-embedded atoms in which the metal atom is incorporated into the surface lattice. This can occur through partial substitution of a surface cation, trapping at vacancy sites generated during synthesis or reduction treatments, or more complex mechanisms, such as interstitial incorporation. Among these, vacancy-stabilized sites are commonly reported,^177^ where metal atoms anchor at oxygen vacancies or low-coordinated surface sites.^178^ In these positions, they may adopt geometries that resemble distorted fragments of the host lattice, with coordination numbers similar to those of native cations, or relax into structures more similar to their bulk oxides.^179^ While substitutional incorporation into bulk-continuation cationic sites is often assumed in theoretical models, experimentally distinguishing between surface and subsurface positions is challenging.^180^ Other configurations, such as interstitial incorporation or stabilization at step edges, have also been proposed but remain even less well-defined structurally.

Under certain synthesis or activation conditions, isolated atoms may migrate to subsurface positions, for example, during high-temperature treatments or reduction steps that promote lattice diffusion.^181^ These sites typically display higher coordination numbers and greater thermodynamic stability but reduced catalytic accessibility, although some studies have proposed a synergistic effect of subsurface on surface-stabilized metals.^182^ The resulting distribution of adatom, surface-embedded, and subsurface configurations is determined by the synthesis pathway, defect structure, and the thermodynamic competition between metal–support interactions and aggregation into bulk metallic or oxidic phases.

This distribution can be considered controllable but rarely monodisperse. Synthetic variables such as the support polymorph and termination, defect density, metal content, precursor chemistry, thermal history, and quench rate can bias isolated atoms toward surface-accessible, lattice-incorporated, or subsurface positions. For example, flame-based synthesis can favor surface-localized lattice incorporation by combining high-temperature metal–support equilibration with rapid cooling, whereas precipitation routes may promote deeper bulk incorporation or mixed surface–bulk distributions.^176^ Even when one configuration dominates, minority environments are likely to coexist and may be difficult to resolve by ensemble-averaged spectroscopy.

These principles are illustrated by mixed-oxide systems for carbon dioxide hydrogenation, where synthesis determines whether promoter or dopant atoms occupy surface lattice positions, subsurface or bulk sites, or more labile surface-deposited environments. In ZnZrO_*x*_, flame-based synthesis favors surface-localized, atomically dispersed Zn^2+^ sites and vacancy-containing ensembles, whereas coprecipitation promotes deeper incorporation and different surface–bulk distributions.^183^ In In-based oxide catalysts, the choice of support and surface structure controls the stabilization of isolated In environments and the formation of oxygen-deficient or hydroxylated sites.^176^ Similarly, in Co-promoted In_2_O_3_/m-ZrO_2_, lattice-incorporated cobalt preserves isolated Co–O–Zr coordination under reaction conditions, whereas surface-deposited cobalt restructures into Co–In phases.^184^ Together, these examples show that oxide-based SACs often contain synthesis-dependent distributions of environments whose catalytic relevance depends on which configurations remain accessible and stable under operation.

The catalytic consequences of these motifs differ substantially. Adatoms are generally more accessible and can bind reactants or intermediates readily, but they may also be more mobile and more sensitive to adsorbate-induced restructuring. Surface-embedded atoms provide stronger anchoring and more direct coupling to lattice oxygen, vacancies, hydroxyls, and support redox chemistry, enabling cooperative metal–oxide ensembles. Subsurface atoms are often thermodynamically stabilized and can influence nearby surface sites electronically or through defect formation, but their direct participation in bond activation is less straightforward. Reaction conditions can further alter these distributions by changing vacancy concentration, hydroxylation, adsorbate coverage, or the redox state of the oxide. Thus, the relevant active environment under operation may differ from the ex situ structure assigned after synthesis, and the measured performance may reflect an ensemble of accessible configurations rather than a single static site.

Beyond the immediate coordination shell, the secondary environment encompasses structural and chemical features in the vicinity of the metal center that do not directly coordinate it, yet nonetheless influence its electronic structure, stability, and reactivity. On oxide supports, these include nearby defects such as oxygen vacancies, surface hydroxyl groups, neighboring alkali cations, and proximal metal centers (Figure 15). For instance, oxygen vacancies that are proximal but not in the first coordination sphere of the metal center can modulate charge distribution around the isolated atom. Similarly, hydroxyl groups in the surrounding surface network may engage in hydrogen bonding or transient ligand exchange, affecting adsorption energetics and catalytic behavior.^185,186^ Although synthetic regulation of secondary local environments in oxide-based SAC remains in its infancy, recent studies have demonstrated that these environments can be deliberately tailored while preserving a largely unchanged primary coordination sphere. This can be achieved through postsynthetic lattice reconstruction, as exemplified by mild hydrogen reduction of Pt_1_/TiO_2_, which induces hydrogen spillover and generates neighboring oxygen vacancies and Ti^3+^ species, thereby establishing a Pt@O–Ti^3+^ secondary environment that enhances interfacial charge transfer.^187^ Alternatively, secondary local environments can be regulated by controlling the spatial location of isolated atoms within the host lattice; for example, partial incorporation of iridium single atoms into the NiO lattice increases second-shell Ir–Ni coordination while largely preserving the primary Ir@O motif, strengthening metal–support interactions and improving OER performance.^188^

**Figure 15:**
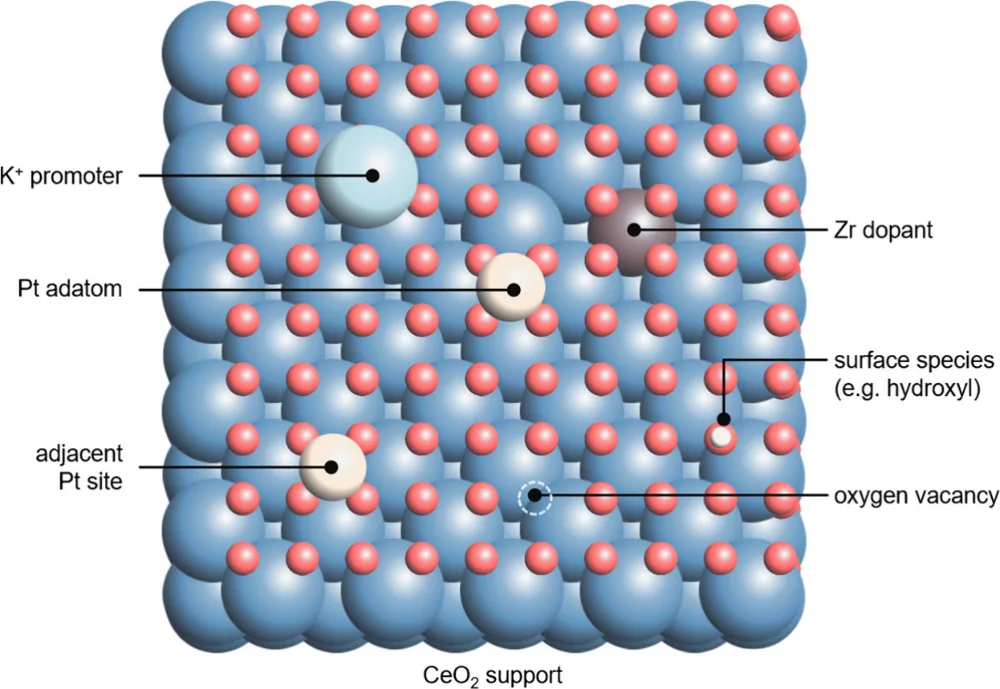
Local chemical features influencing secondary and extended environments of isolated metal atoms on oxide supports, within a broader context of structural variability of the support.

Promoter species such as alkali metals or dopant cations can further alter the local environment of isolated atoms by tuning surface acidity and basicity, modifying defect formation energies, or facilitating charge transfer. However, in contrast to the primary coordination sphere, the spatial relationship between these secondary features and the metal center is often poorly defined, and their role must typically be inferred from indirect spectroscopic signatures or reactivity trends. Synthetic control over these features remains limited, as their distribution depends strongly on several factors and is often nonuniform. As a result, even when the primary coordination motif is well-defined, SACs often exhibit a family of local environments that differ subtly in their surrounding lattice context, giving rise to distributions of active site behavior rather than single, uniform configurations.

The extended environment, defined by the broader crystallographic and morphological structures of the support, may also play an important role in shaping the properties of oxide-supported single atoms. This environment addresses features such as particle morphology and the corresponding distribution of surface facets, pore architecture and related three-dimensional structure, and electronic or ionic conductivity. These parameters influence factors such as the structure and distribution of metal centers, their accessibility, and the extent of electronic coupling to the support lattice. For example, different crystallographic facets can stabilize isolated atoms in distinct geometries.^189,190^ Similarly, facet- or nanostructure-dependent variations in reducibility have been linked to the stability of single atoms toward sintering under reducing conditions.^124^

Recently, the use of high-k dielectric oxides has been proposed as a strategy for enhancing single-atom stabilization. Their high permittivity enables greater charge delocalization and polarization, which was linked to the formation of fully reduced oxygen vacancies near hydroxylated indium atoms stabilized on monoclinic hafnium oxide.^176^ In practice, however, controlling this extended environment remains challenging. Even when specific polymorphs or morphologies are targeted, the surface structure can differ from the ideal bulk termination and may be reconstructed under the reaction conditions. Porous frameworks and hierarchical architectures further shape the coordination environment by imposing geometric confinement, which can restrict atom migration, enhance stabilization through spatial proximity, or alter the distribution of local environments through pore wall curvature and connectivity.^191,192^

As a result of all these factors, oxide-based SACs often exhibit variations in the extended environment that contribute to the diversity of local coordination motifs observed experimentally. Importantly, many features discussed in the context of the secondary and extended environments, such as hydroxyl groups, alkali promoters, or support dopants, do not belong exclusively to one spatial domain. Depending on their proximity and interaction strength, they can straddle the primary coordination sphere, the second coordination layer, and even the broader support structure. Similarly, isolated metal atoms are not passive occupants of preexisting environments. Their presence can induce local restructuring, modifying the defect distributions, oxygen coordination, and even lattice strain. In this way, the atom defines its own immediate environment and feeds back into the properties of the support.

The crystallographic compatibility between the adjacent domains of the support can also play a stabilizing role. Structural mismatch or symmetry incompatibility between dispersed and bulklike phases may inhibit overgrowth or sintering, thus promoting the persistence of isolated or highly dispersed species. Such effects have been proposed, for example, to explain the high stability of dispersed In@O_*x*_ species on monoclinic zirconia, where symmetry mismatch prevents agglomeration into bulk indium oxide.^193^

Although rarely considered in catalyst design, these symmetry-related stabilization mechanisms represent an underexplored complement to traditional defect-engineering and ligand-coordination strategies. These structural principles extend beyond oxides to other crystalline hosts used in SACs, including carbides, nitrides, and mixed-anion systems.^194^ As in oxides, isolated atoms in these materials are stabilized through interactions with lattice anions, vacancies, or substitutional sites. However, the greater structural complexity and more limited synthetic tunability of these supports can constrain the precise control over the resulting local environments.

### 4.2 Carbon-Based Single-Atom Catalysts

Carbon-based materials represent one of the most extensively studied classes of supports for SACs.^26,195^ Their widespread use stems from their structural versatility, high surface area, and the ability to introduce heteroatoms that act as ligands for isolated metal atoms. In contrast to crystalline inorganic supports, where coordination environments are largely dictated by the host lattice, carbon frameworks provide a more flexible platform in which the local coordination of the metal center can be engineered through precursor design and thermal processing. As a result, carbon-based SACs have enabled a wide range of metal–ligand environments and have been particularly prominent in electrocatalytic applications, where metal–nitrogen–carbon (M–N–C) catalysts have been investigated for reactions such as oxygen reduction, carbon dioxide reduction, and nitrogen reduction.^196,197^

Carbon supports are typically obtained through the thermal transformation of precursors in which metal and heteroatom sources are introduced either concurrently or sequentially, including molecular complexes, metal–organic frameworks (MOFs), or polymeric templates. Precursor composition, heteroatom incorporation, and pyrolysis conditions collectively determine the resulting carbon framework, including the distribution of edge and basal-plane sites, vacancy structures, graphitic and amorphous domains, as well as the identity, concentration, and spatial arrangement of heteroatoms.^138^ Consequently, carbon materials present a heterogeneous landscape of chemically distinct anchoring sites that varies both between different materials and within individual supports, defining a broad distribution of accessible local environments. Nitrogen-rich ligands or MOF-derived templates preferentially promote M@N_*x*_ environments, whereas oxygen-containing coordination, mixed heteroatom environments (N, O, S, P), and defect-mediated binding become increasingly prevalent depending on the precursor chemistry and thermal treatment.^198,199^ Although synthetic parameters strongly bias the distribution of local environments, their formation remains fundamentally statistical rather than deterministic.^26,195^

Several anchoring mechanisms have been proposed for stabilizing isolated metal atoms in carbon frameworks, including heteroatom coordination, defect anchoring, adsorption on π-conjugated carbon surfaces, and geometric confinement within specific support structures (Figure 16a). Among these, heteroatom coordination, most notably M@N_*x*_ motifs in doped carbons, and defect-mediated anchoring at vacancies or edge sites are the most widely established, providing strong binding and well-defined coordination environments.^195,200^ In contrast, stabilization via adsorption on pristine π-conjugated surfaces is generally weaker and less frequently observed under catalytic conditions.^201^ Geometric confinement arises from pores, cavities, or interlayer regions that spatially isolate single atoms, while local chemical interactions with the carbon framework, including coordination to heteroatoms and bonding at defect sites further restrict metal mobility.^202^ In practice, geometric and chemical contributions often act synergistically to stabilize isolated atoms.

**Figure 16:**
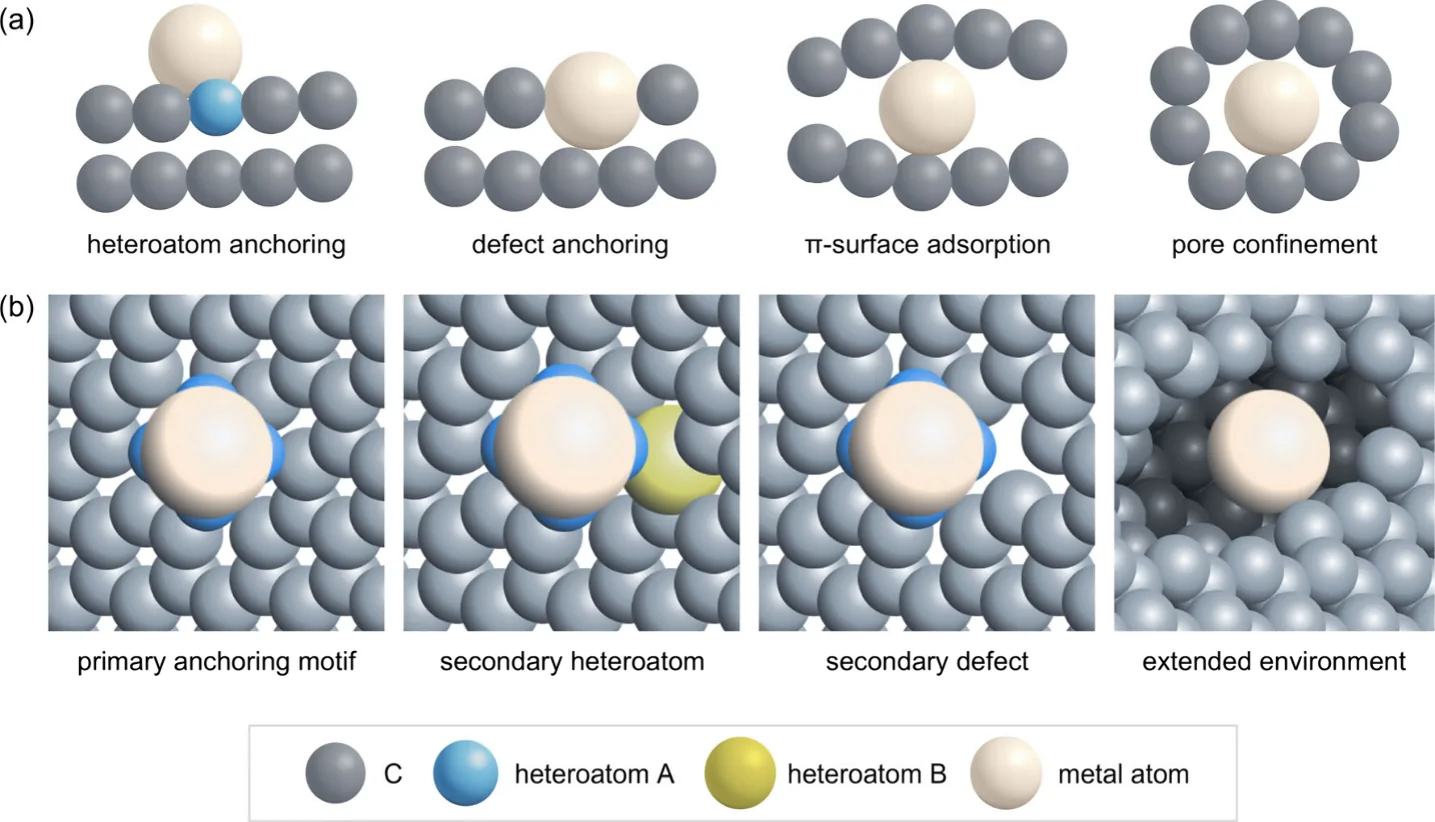
(a) Representative anchoring modes including heteroatom anchoring, defect anchoring, π–surface interaction, and confinement within micropores. (b) Hierarchy of environments surrounding anchored metal atoms, from the primary coordination motif to secondary and extended structural influences of the carbon framework.

Nitrogen-coordinated environments are the most extensively studied and are commonly represented by idealized motifs, such as M@N_4_,^203,204^ M@N_3_C,^205,206^ and related configurations. While these descriptors conveniently classify the nominal primary coordination sphere, they can also encourage an overly discrete description of the active site. First, they reduce the diversity of coordination chemistries present within a material to a limited set of nominal motifs. Second, even a descriptor such as M@N_4_ does not identify a unique local environment, as variations in carbon topology, edge versus basal plane location, vacancy structure, graphitic domain size, local distortions, and the identity and spatial arrangement of neighboring heteroatoms generate a continuum of structurally and electronically distinct environments. Accordingly, descriptors such as M@N_4_ should be regarded as shorthand for the nominal primary coordination sphere rather than complete descriptions of the catalytically active site.

Moving beyond nominal coordination motifs requires experimental and computational approaches capable of resolving distributions of local environments. This remains challenging because variations in coordination chemistry, defect structure, graphitic domains, edge sites, and neighboring heteroatoms generate overlapping spectroscopic signatures that are difficult to disentangle. Conventional characterization techniques, including XAS, Mössbauer spectroscopy, XPS, and EPR, provide complementary but ensemble-averaged information, whereas atomistic models typically examine only a limited number of candidate structures rather than the statistical distributions produced during synthesis. Consequently, local-environment heterogeneity is currently inferred through the combined interpretation of complementary spectroscopic measurements and atomistic modeling rather than directly resolved. Emerging approaches, such as solid-state NMR integrated with statistical modeling, are beginning to overcome this limitation by experimentally resolving distributions of coordination environments instead of assigning a single nominal coordination motif.^55^

In parallel, recent efforts have focused on deliberately breaking the symmetry of idealized coordination motifs, for example, by generating undercoordinated M@N_*x*_ sites, introducing heteroatom asymmetry, or creating bimetallic environments, to tune electronic structure and adsorption properties.^203^ Beyond the immediate coordination sphere, increasing attention has also been devoted to engineering the secondary environment, which in carbon-based SACs is defined by the arrangement of neighboring heteroatoms, defects, and functional groups within the carbon matrix (Figure 16b).^169^ In contrast to the primary coordination sphere, these species do not necessarily bind directly to the metal center but can significantly influence its properties through electronic coupling mediated by a conjugated carbon framework. For example, NC supports typically contain distributions of pyridinic, pyrrolic, and graphitic nitrogen species, whose relative arrangement can influence the charge density and spin state of the metal center.^207^ Building on this concept, recent synthetic strategies have enabled selective regulation of secondary local environments while largely preserving the primary coordination environment through the introduction of heteroatoms into the surrounding carbon lattice. This has been achieved through approaches such as precursor precoordination,^208^ salt-templated pyrolysis,^209^ and postsynthetic heteroatom functionalization,^210^ which direct O, S, or P atoms into the surrounding carbon lattice rather than the first coordination shell, thereby tuning charge redistribution, metal–support electronic coupling, and adsorption energetics through second-shell interactions.

However, disentangling the contribution of specific neighboring sites remains challenging, as these environments are inherently heterogeneous, and theoretical models often rely on simplified, idealized structures that do not fully capture the complexity of the second coordination sphere. Additional heteroatoms, including sulfur, phosphorus, or boron, are often introduced as codopants, providing an additional degree of electronic and chemical modulation.^64,210,211^ A key challenge in these systems is that the spatial relationship between the metal center and surrounding species is rarely well-defined, owing to the disordered and heterogeneous nature of carbon supports.^110^

This knowledge becomes particularly critical for the development of advanced architectures, such as DAC and GAC, where the catalytic behavior depends sensitively on the distance, orientation, and interaction between neighboring anchoring motifs. This makes it difficult to isolate the contribution of individual features and highlights the importance of approaches that can probe or model these environments in a spatially resolved and statistically meaningful manner.

The extended environment of carbon-based SACs is further defined by the broader structural features of the carbon framework, including graphitic domain size, degree of graphitization, porosity, and electronic conductivity.^198^ These properties influence and are often invoked to rationalize both the stability of isolated atoms and the transport of reactants, electrons, and phonons during catalysis. For example, highly graphitized carbon structures are generally associated with improved electronic conductivity but a reduced density of anchoring sites,^212^ whereas more defective carbon matrices are often assumed to enable higher densities of isolated atoms, albeit within more heterogeneous environments. However, direct experimental evidence establishing this trade-off in a decoupled manner remains limited as graphitization, defect density, and local coordination are typically interdependent.

Beyond local defect chemistry, the topology of the carbon framework, including micro- and mesoporosity, has been widely explored to enhance transport properties and active site accessibility.^213,214^ However, direct correlations between these structural parameters and catalytic performance remain limited as variations in pore architecture, graphitization, and local coordination environments are often intrinsically coupled.

### 4.3 Generalization Across Support Families

The previous sections established oxide- and carbon-based supports as two complementary strategies for generating local environments in SACs. In oxide-based SACs, local environments are primarily constrained by the crystallographic lattice through metal–support interactions, whereas in carbon-based materials they emerge predominantly from synthetic processes that create heteroatom coordination motifs and defect structures.

Other support families can be understood as distinct realizations within this same framework rather than fundamentally different paradigms (Figure 17). Nitrides,^215,216^ carbides, and MXenes lie close to the lattice-derived end of the continuum because local environments remain closely linked to the crystalline host, although increased covalency and, in the case of MXenes, chemically tunable surface terminations expand the range of accessible coordination environments.^217^ Sulfides and phosphides occupy an intermediate position, where softer anion chemistries provide greater flexibility, while local environments are still strongly influenced by the underlying lattice.^218^ Zeolites likewise rely on a well-defined framework to generate local environments, with geometric confinement becoming an additional determinant of site accessibility and reactivity.^219^ In contrast, MOFs and COFs increasingly create local environments through the deliberate design of the framework itself, bridging lattice-defined coordination and the precursor- and defect-directed environments characteristic of carbon supports.^220–222^

**Figure 17:**
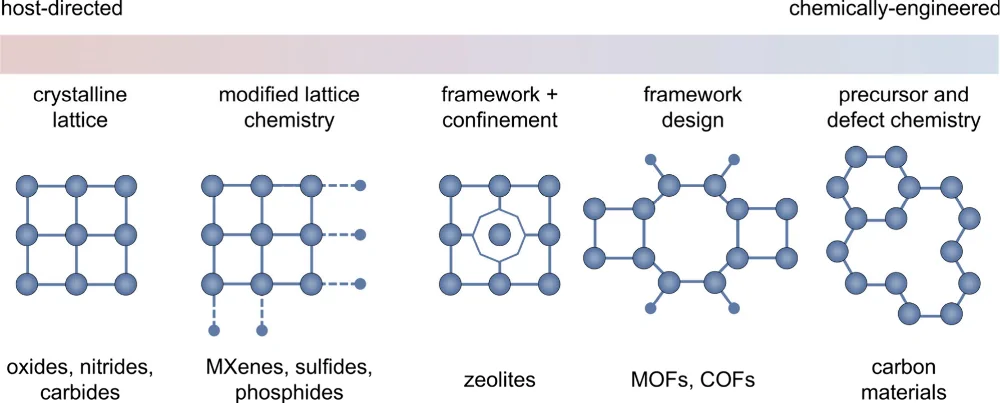
Conceptual continuum of local environment formation across different support families for SACs.

Viewed from this perspective, support families are distinguished not by different principles of local environment formation but by the relative contributions of intrinsic support chemistry and synthetic design in creating the population of accessible local environments. This framework therefore unifies diverse support chemistries according to the strategy by which local environments are generated, providing a common basis for understanding and comparing SACs across support classes.

### 4.4 Metal Introduction Strategies

While the nature of the support largely determines the coordination motif types that stabilize isolated metal atoms, the method of metal species introduction plays a critical role in determining how these environments form and are distributed within the resulting catalyst. This impacts the accessibility of anchoring sites, the probability of aggregation during synthesis, and the final density and spatial distribution of the isolated atoms. Consequently, the formation of SAC environments depends not only on the support chemistry but also on the kinetic and thermodynamic pathways through which metal atoms interact with the support during synthesis and activation.

#### 4.4.1 Postsynthetic Wet-Chemistry Routes

Several general approaches are used to introduce metal atoms into the catalyst supports. In postsynthetic wet-chemistry methods, among the most prevalent and scalable routes for SAC preparation, metal precursors are deposited onto a preformed support through techniques such as impregnation,^39^ ion exchange, or deposition-precipitation. In these processes, the metal precursor interacts with surface functional groups, defects, or anchoring sites present on the support before subsequent activation treatments. Because many supports expose such functionalities, these approaches are broadly applicable across a wide range of materials, including oxides, carbon-based supports, and related inorganic solids.^39^ At this stage, the metal species may already be present as isolated ions or molecular complexes dispersed across the surface. However, these species are often only weakly anchored and typically retain ligands that originate from the precursor. As a result, the coordination environment of the metal center remains largely defined by the precursor chemistry rather than by strong interactions with the support.^92^

Subsequent activation treatments, typically involving thermal processing, play a decisive role in establishing the final coordination environment of isolated metal atoms. During these treatments, ligands associated with the precursor are progressively removed, allowing the metal center to form stronger interactions with the support. For example, weakly bound ligands such as nitrates are often removed at relatively low temperatures, whereas more strongly bound species such as chlorides may require higher temperature treatments.^39^ These processes can significantly alter the primary coordination sphere of the metal center and the chemical state of the support.

At elevated temperatures, activation may also modify the support itself. Carbon-based materials can undergo structural rearrangement or local carbonization, while oxide supports may experience dehydroxylation, defect formation, or changes in surface structure. Consequently, the evolution of the metal environment during activation is closely coupled with changes in the support. Careful control of ligand removal and activation conditions is therefore a key strategy in SAC synthesis (Figure 18).^39^ By enabling the gradual formation of strong metal–support bonds while minimizing metal mobility, these treatments help stabilize isolated atoms at high densities and prevent uncontrolled aggregation into clusters or nanoparticles.

**Figure 18:**
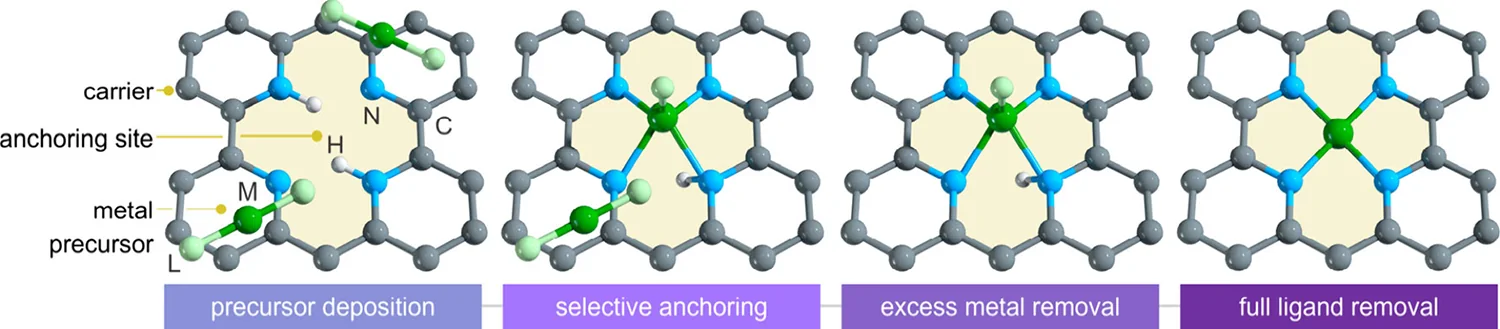
Stepwise site formation illustrating how controlled precursor decomposition promotes isolated metal site formation. Adapted from ref 39.

#### 4.4.2 Direct Synthesis Routes

In contrast, direct synthesis routes generate isolated metal sites during formation of the support itself. In these approaches, the metal precursor is incorporated into the synthesis mixture and the coordination environment of the metal atom develops concurrently with the formation of the host material. Examples include pyrolysis of metal-containing molecular precursors or MOFs, polymerization-based syntheses of carbon materials, coprecipitation and hydrothermal methods used for oxide supports, and high-temperature processes such as flame spray pyrolysis.^223^ Because the metal species is present during support formation, these methods can enable the stabilization of isolated atoms through lattice incorporation, coordination with heteroatoms generated during synthesis, or trapping at defects formed during crystallization.

Direct synthesis routes are particularly common in the preparation of carbon-based SACs, where pyrolysis of MOF, polymers, or other nitrogen-containing precursors generates metal–heteroatom coordination environments simultaneously with the carbon framework. MOF are frequently used as precursors for carbon-based SACs because their metal–ligand coordination and heteroatom-rich frameworks provide a high density of well-distributed anchoring sites that can be partially preserved during pyrolysis, facilitating the formation of M@N_*x*_ coordination motifs. However, the multistep synthesis of many MOF and their limited scalability have motivated the development of simpler precursor systems for practical catalyst preparation. Analogous direct strategies are also widely used for oxide-based SACs, for example, through coprecipitation or flame spray pyrolysis, where isolated metal atoms may become incorporated into the oxide lattice or stabilized at defects formed during particle growth.

The mechanisms governing site formation in direct synthesis vary significantly across these approaches. In some systems, the primary coordination environment is strongly influenced by the chemical structure of the precursor, particularly when metal–ligand complexes template specific coordination motifs. In others, the final environment is determined by the thermodynamic stability of metal incorporation into the host lattice or by kinetic trapping during rapid particle formation. As a result, direct synthesis routes can produce isolated metal environments through a combination of precursor-directed coordination, lattice incorporation, and defect-mediated stabilization, depending on the synthesis conditions and support chemistry.

#### 4.4.3 Vapor-Phase Deposition Methods

A third class of strategies involves vapor-phase deposition methods,^224^ including atomic layer deposition (ALD),^225,226^ chemical vapor deposition (CVD), and physical deposition techniques, such as sputtering or evaporation. In ALD, metal species are introduced through self-limiting, surface-mediated reactions, enabling precise control over the metal content and deposition chemistry at the atomic scale.^227^ In contrast, CVD and physical deposition methods rely on continuous or kinetically controlled processes, which do not inherently limit growth at the atomic level but can, under carefully tuned conditions, yield highly dispersed metal species.^228^ As a result, while ALD offers the most direct route to uniform deposition at low contents, comparable levels of dispersion can, in some cases, be approached with other vapor-phase methods through control of precursor flux, temperature, and support interactions.

Despite the control afforded during deposition, the final coordination environment of isolated metal atoms remains largely determined by the nature of the support and the availability of the anchoring sites. Subsequent thermal treatments are often required to remove precursor ligands and stabilize the deposited species, during which the metal atoms may reorganize into coordination motifs dictated by the support surface. These postdeposition processes can also modify the surrounding support structure, including surface hydroxylation, defect populations, and local lattice structure, thereby influencing the secondary and extended environments of the metal centers, while the primary coordination sphere is ultimately constrained by the anchoring sites provided by the support.

#### 4.4.4 Key Variables and Design Constraints

Beyond the formation of isolated atoms, metal introduction strategies must be evaluated by how reliably they define the resulting local environment. Relevant criteria include the degree of control over the coordination motif, breadth of site distribution, reproducibility across batches, persistence during activation treatments, feasibility of transfer to larger scales, and safety and sustainability of the synthesis and application. These aspects are particularly important because catalysts that appear atomically dispersed by ensemble characterization can still contain distinct surface, embedded, subsurface, or aggregated minority environments. Table 3 summarizes common strategies for introducing single atoms in terms of controllability, reproducibility, and scalability. Two highly illustrative examples include two-step annealing strategies for ultrahigh-density SAC libraries, where controlled precursor decomposition and stepwise ligand removal enable high metal contents while limiting thermally induced aggregation.^39^ Complementary cascade-anchoring approaches for M/N–C catalysts illustrate how chelation, physical isolation of metal complexes, and nitrogen coordination during pyrolysis can be combined to increase isolated-site density while retaining scalable preparation.^229^

**Table 3:** Practical Comparison of SAC Synthesis Strategies for Local-Environment Design

strategy	control variables	controllability	reproducibility concerns	scalability
impregnation/incipient wetness	precursors, content, solvent, drying, activation	moderate; broad site distributions common	support heterogeneity, precursor stability, drying gradients, activation protocol	high, but scale up may alter speciation
deposition-precipitation/adsorption methods	pH, surface charge, precursor hydrolysis, aging	moderate to high when anchoring sites are defined	sensitive to pH, aging, washing, and local precipitation	scalable, but batch control is demanding
atomic layer deposition/molecular grafting	precursor chemistry, surface functionality, pulse/grafting cycles	high at low metal contents	residual ligands and surface pretreatment	precise but less practical at large scale
ion exchange/framework incorporation	exchange sites, framework charge	high in well-defined hosts	incomplete exchange, framework instability, post-treatment	scalable for robust frameworks
defect trapping	defect type and density, activation treatments	potentially high	defect heterogeneity and evolution	scalable if defect generation is robust
flame spray pyrolysis	composition, flame conditions, quench rate	moderate, control of composition and dispersion in oxides	flame parameters, precursor compatibility, phase segregation thresholds	attractive for scale-up
lattice doping/coprecipitation/hydrothermal synthesis	lattice compatibility, dopant solubility, crystallization pathway	high if solubility limits are respected	mixed surface-bulk distributions, or phase changes	scalable, site accessibility may be low

Across these different approaches, several synthesis variables play a central role in determining the formation of isolated metal environments. These include the structure of the single-atom precursor, the density and nature of anchoring sites on the support, the metal content, the presence of promoters or other deliberately introduced catalyst components, and the thermal history of the catalyst during synthesis and activation. Compared with support engineering, however, deliberate promotion of SAC local environments remains relatively underexplored,^230^ particularly in studies that resolve the spatial and chemical relationship between promoter and active center sufficiently well to establish environment-function correlations. Strong metal–support interactions and high anchoring site densities favor the stabilization of isolated atoms, whereas insufficient anchoring capacity promotes metal aggregation and nanoparticle formation. By reshaping the thermodynamic and kinetic landscape governing metal dispersion, these interactions suppress atom migration and stabilize isolated sites. Under appropriate stimuli, they may also induce the strong metal–support interaction (SMSI) phenomenon, where interfacial restructuring, including support decoration, oxide overlayer formation, or partial to complete encapsulation of the active site, can either enhance catalyst stability and performance or reduce active-site accessibility, depending on the catalyst and reaction conditions.^231–234^ Thermal treatments can further influence site distributions by enabling diffusion, ligand removal, lattice incorporation, or support reconstruction. These variables determine not only the fraction of metal present as isolated atoms, but also the diversity of coordination motifs, and the spatial arrangement of metal centers on the support.

One central challenge is the maximum density of isolated metal atoms that can be stabilized on a given support. This limit is governed by the density of available anchoring sites and by the thermodynamic competition between metal–support interactions and metal–metal bonding. When the metal content approaches the stabilization capacity of the support, aggregation into clusters or nanoparticles becomes increasingly likely. Strategies to increase site density often focus on increasing the number of anchoring sites through defect engineering, heteroatom doping, or the use of molecular precursors that template metal coordination environments during synthesis. The importance of anchoring-site density is illustrated by graphene quantum dot-mediated syntheses that achieve high transition-metal contents by increasing the density of stabilizing coordination environments,^235^ and by atom-trapping approaches on ceria, where high-temperature treatment populates the most stable anchoring sites and yields sinter-resistant isolated Pt atoms.^186^ Nevertheless, even in systems that can effectively stabilize isolated atoms, distributions of site densities and coordination environments arise due to heterogeneity in the support structure.

Synthesis methods also influence the accessibility of the metal sites during catalysis. Depending on the preparation route and support structure, metal atoms may occupy surface, subsurface, or embedded positions within the support. In oxide-supported systems, high-temperature treatments can promote the incorporation of metal atoms into subsurface lattice positions, while in carbon-based materials, some metal centers may become partially embedded within graphitic domains. Although such environments can enhance stability, they may also reduce the accessibility of the metal center to the reactants. Achieving an optimal balance between stability and accessibility, therefore, represents an important design consideration for SAC synthesis.

Precursor chemistry can exert a lasting influence on SAC local environments through precursor-directed site formation. Rather than implying literal retention of precursor ligands, this concept describes how ligand identity, metal coordination geometry, charge, nuclearity, hydrolysis behavior, and decomposition pathway bias the initial metal–support interaction during deposition, drying, calcination, or reduction. In some systems, these effects are erased during activation treatments, and the final site is governed mainly by the support and reaction environment. In others, the precursor determines the anchoring geometry, dispersion state, or activation pathway strongly enough that aspects of the synthetic history persist into the working catalyst. Precursor choice should therefore be treated as an explicit local-environment design variable, because different precursor families can generate different site distributions even at identical metal content and support composition.

Finally, practical considerations such as scalability and reproducibility, safety, and sustainability further determine the utility of the SAC synthesis strategies. Wet-chemistry- and pyrolysis-based methods are generally compatible with large-scale catalyst preparation and have therefore been widely adopted in both academic and industrial research. Carbon nitride-based SAC syntheses further show that precursor choice, condensation conditions, support surface area, synthesis yield, and continuous-flow performance must be considered together when moving beyond small-batch preparation.^236^ Safety-driven catalyst replacement provides another important dimension: Pt_1_/AC catalysts for acetylene hydrochlorination illustrate how isolated metal sites can be designed to replace toxic Hg-based systems in vinyl chloride production, provided that the local environment also ensures stability under corrosive reaction conditions.^92^ In contrast, ALD and molecular grafting offer higher control over surface coverage and metal–support bonding but require more stringent precursor and reactor constraints and are less straightforward to translate to large-scale catalyst production.^226^ Thus, the practical value of a metal introduction strategy is not defined only by its ability to suppress nanoparticle formation but by whether it reproducibly delivers a controlled local-environment distribution that survives activation, remains accessible under catalytic conditions, and addresses the practical constraints of the target process.

## 5 Local Environments under Reaction Conditions

The local environments established during synthesis define the initial catalyst landscape, which continues to evolve under reaction conditions through the dynamic processes introduced in Section 2.4. These processes have two distinct catalytic consequences. They govern catalytic mechanisms through adaptive changes of local environments and cooperative interactions with their surroundings while also driving the progressive structural evolution of the catalyst by reshaping the distribution of local environments available during operation. Although these phenomena are commonly grouped under the broad concept of catalyst dynamics, doing so obscures their fundamentally different catalytic roles. Here, they are instead organized according to their catalytic function, separating the processes that govern catalytic mechanisms through adaptive and cooperative local environments (Section 5.1) from those that progressively reshape the catalyst through structural evolution of local environments under reaction conditions (Section 5.2).

### 5.1 Mechanistic Roles of the Local Environment

Mechanisms of SAC-catalyzed reactions were initially interpreted mostly in terms of electronic metal–support interactions, where the surrounding matrix tunes the electronic structure of the isolated atom and thereby its adsorption behavior and reaction rates.^23^ However, under operating conditions, the coordination environment does not simply perturb a fixed active site but instead governs how reactants bind, transform, and desorb, while stabilizing the coordination states through which catalytic transformations proceed.^52,237,238^ This, in turn, determines the reactivity patterns and mechanisms that emerge. As a result, mechanistic descriptions must also account for the broader local environment, including direct contributions from the support and, where relevant, interactions between neighboring metal sites. The following examples show how concepts introduced in Section 2.6, including adaptive coordination, confinement, and secondary-sphere regulation, provide a basis for interpreting and designing catalytic function in SACs.

#### 5.1.1 Adaptive Coordination within the Catalytic Cycle

Catalytic turnover at an isolated metal center emerges from the ability of the local coordination environment to continuously adapt throughout the catalytic cycle. As reactants bind, transform, and desorb, the active site reversibly evolves through a series of closely related coordination states that accompany the successive elementary steps of the catalytic cycle.^90,239^ This requires the accessibility of several coordination configurations of comparable free energy, enabling the metal center to reversibly interconvert between closely related states in response to the changing chemical potential imposed by the reaction environment.

These reversible transitions involve simultaneous adjustments to both the atomic structure and the electronic structure of the local environment. Bond elongation or compression, coordination opening or closure, and transient changes in the oxidation state proceed concurrently with electronic relaxation within the local environment. In many cases, adsorbates temporarily become part of the coordination sphere, inducing reversible coordination switching as intermediates bind and are released during the catalytic cycle.

Structural and electronic responses are therefore intrinsically coupled, and the ability of a catalyst to sustain turnover depends on how effectively the local environment accommodates these concerted adjustments. This interplay is particularly evident in electron-transfer (ET) and proton-coupled electron-transfer (PCET) mechanisms, where charge transfer is intrinsically linked to reversible adaptations of the first coordination sphere. The extent of these structural adjustments governs the associated reorganization energy and can therefore strongly influence charge-transfer kinetics, directly coupling coordination-environment evolution to catalytic turnover.^240–243^ For example, electrochemical polarization of Fe@N_4_ sites reversibly generates Fe–OH species that transiently become part of the first coordination sphere, acting as proton–electron relay centers and establishing an inner-sphere PCET pathway for stepwise uranium reduction.^244^

The adaptive behavior is largely governed by the nature of the support, which defines the accessible adaptive space of the local environment, while the reaction conditions determine the extent to which this space is explored during catalysis. (Figure 19a).^245^ In systems with geometric confinement, the accessible coordination window is largely defined by the topology of the coordination pocket. For example, in graphitic carbon nitride (gC_3_N_4_), the carbon nitride framework constrains how metal–ligand distances evolve during the catalytic cycle by defining the three-dimensional space over which the isolated metal center can reversibly displace while remaining anchored (Figure 19b). In Pd_1_/gC_3_N_4_ the evolution of the Pd–N coordination along the Suzuki coupling pathway illustrates how dynamic metal–support interactions enable access to a quasi-continuous set of coordination states while maintaining anchoring.^246^ In such systems, cavity geometry defines the reversible range of structural distortion available during turnover, showing how adaptive coordination can be translated into SAC design when the support framework controls the accessible coordination window.

**Figure 19:**
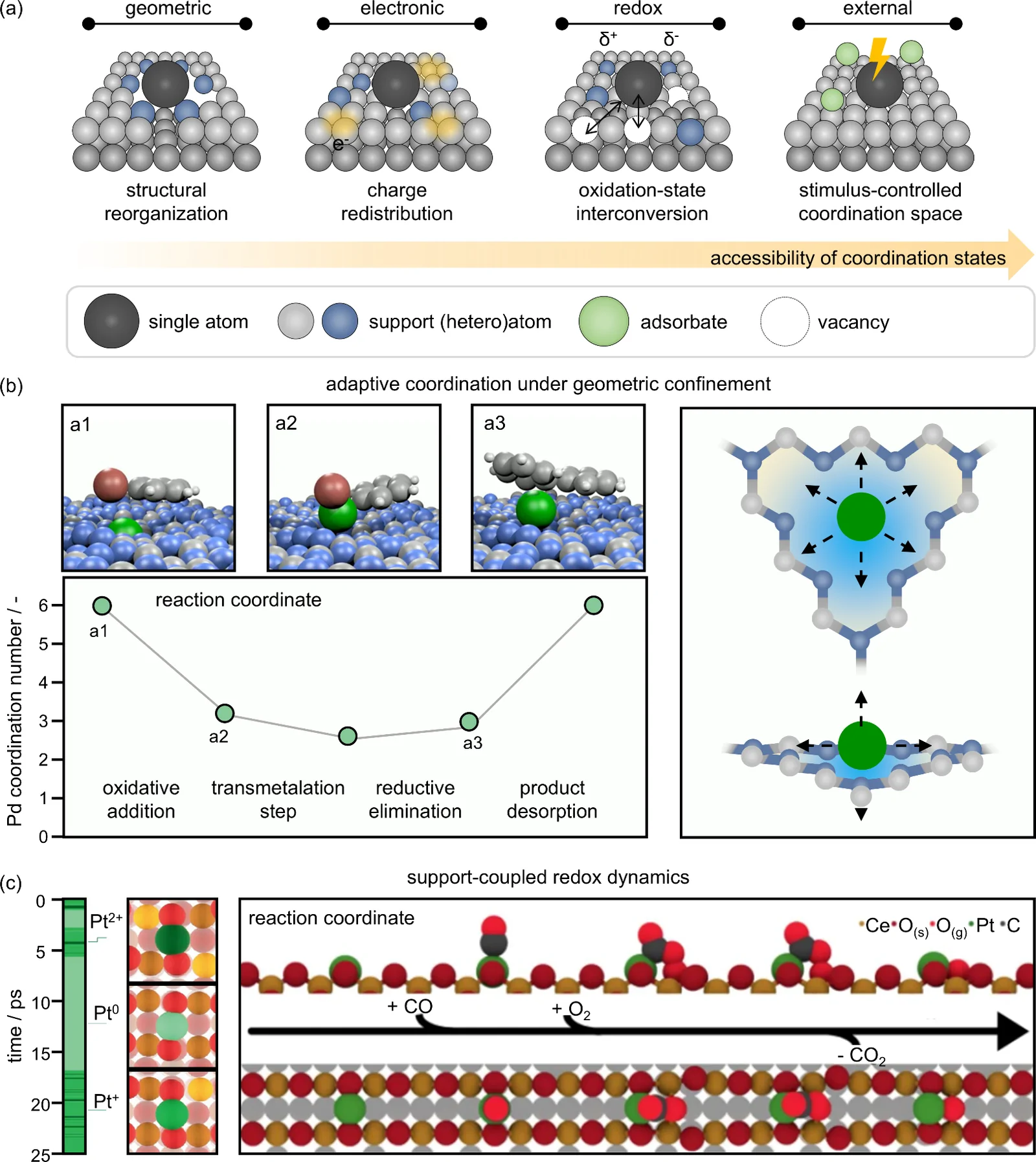
(a) Different mechanisms enabling adaptive coordination of the active site. (b) Evolution of the Pd coordination number along the Suzuki reaction pathway in Pd_1_/ECN, with representative intermediate configurations highlighted and an idealized schematic illustrating the three-dimensional coordination space accessible to the isolated metal center within the anchoring cavity. (c) Time evolution of the Pt oxidation state during MD at 600 K for a Pt_1_/CeO_2_ SAC together with the coordination changes occurring during the carbon monoxide oxidation cycle. Part (b) adapted from ref 246 and part (c) adapted from ref 85 (Copyright 2019, Springer Nature).

In extended carbon frameworks, adaptive coordination is mediated by electronic delocalization across conjugated π networks. During bond formation and cleavage, charge can be redistributed over the extended framework,^247^ with the identity, density, and connectivity of heteroatom dopants determining how effectively these perturbations are dispersed. As a result, structural changes at the metal center are buffered electronically without requiring significant rearrangement of the support. In contrast, oxide supports generally localize electronic perturbations at the metal–support interface.^238^ The extent of this localization, however, depends on the redox flexibility of the lattice.

Reducible oxides introduce an additional dimension through lattice redox participation.^248,249^ In systems such as Cu_1_/CeO_2_, reversible changes in metal–oxygen bonding accompany carbon monoxide activation, transiently modifying coordination while preserving dispersion.^249^ Lattice oxygen and vacancy formation alter interfacial charge transfer and expand the set of coordination states accessible under chemical potential, which are not statically occupied but dynamically interconnected. In Pt_1_/CeO_2_, for example, CO oxidation drives the catalyst from a stable Pt@O_4_ resting configuration to the more open Pt@O_3_ and Pt@O_2_ environments during activation, while reversible electron exchange with the ceria lattice continuously redistributes the population of Pt^0^, Pt^+^, and Pt^2+^ states. The catalytic cycle therefore proceeds through the concerted evolution of coordination environment, lattice redox state, and metal oxidation state (Figure 19c).^85^ Such behavior is characteristic of reducible oxides, whose lattice can accommodate redox and defect chemistry, whereas more inert oxides generally impose a more rigid coordination environment around isolated metal atoms. In these materials, the redox flexibility of the support determines how far geometric and electronic adaptation can proceed within a reversible regime.

The adaptive nature of the local environment can also manifest through the transient coordination of adsorbates or ligands, enabling catalytic configurations that are stabilized only while these species remain bound.^29^ For example, in Au_1_/CeO_2_, carbon monoxide adsorption induces an Au^+^–CO species at the interface that becomes the active site and later reintegrates into the nanoparticle.^162^ Similarly, by temporarily weakening metal–support interactions or stabilizing alternative coordination motifs, adsorbates can transiently promote multinuclear active sites. A representative example can be found in CO oxidation over Pt_1_/Fe_3_O_4_, where CO induces the reversible formation of catalytically active (PtCO)_2_ dimers, which dissociate after product desorption to regenerate isolated platinum sites.^250^ Similar adsorbate- and potential-driven reversible changes in nuclearity have also been reported for Cu_1_/NC catalysts during electrochemical carbon dioxide reduction^251^ and for Cu_1_/CeO_2_ during electrocatalytic urea synthesis.^252^

External perturbations further delimit the adaptive space.^90^ Electrochemical bias can alter oxidation state, spin configuration,^253–255^ or coordination geometry,^256–258^ while photoexcitation may transiently weaken metal–support interactions or promote charge transfer between the metal center and the surrounding matrix.^259^ Such effects are particularly relevant in photocatalytic SAC systems, where photogenerated carriers modify the electronic structure of the metal–support interface during turnover. These stimuli illustrate a central design constraint: the coordination scaffold must remain structurally coherent across the range of states accessed during operation.

The adaptive space is ultimately governed by a range of structural parameters that shape the accessible configurations of the local environment (Figure 20), serving as a common basis throughout this section. Within this space, the governing descriptors capture the ability of the support to accommodate different coordination states (through support morphology, defect engineering, redox flexibility, and heteroatom doping) and how these are accessed under reaction (through external conditions). The multidimensional and dynamic nature of this adaptive landscape makes its atomistic characterization particularly challenging, requiring theoretical approaches that are capable of resolving subtle structural and electronic rearrangements. DFT enables the quantitative evaluation of elementary steps through adsorption energies and activation barriers, while MD simulations describe the time-dependent evolution of the coordination environment and reveal transient configurations that are not captured by static simulations.^246^ Together, these approaches provide a time-resolved and mechanistically consistent description of how local environments function under reaction conditions.

**Figure 20:**
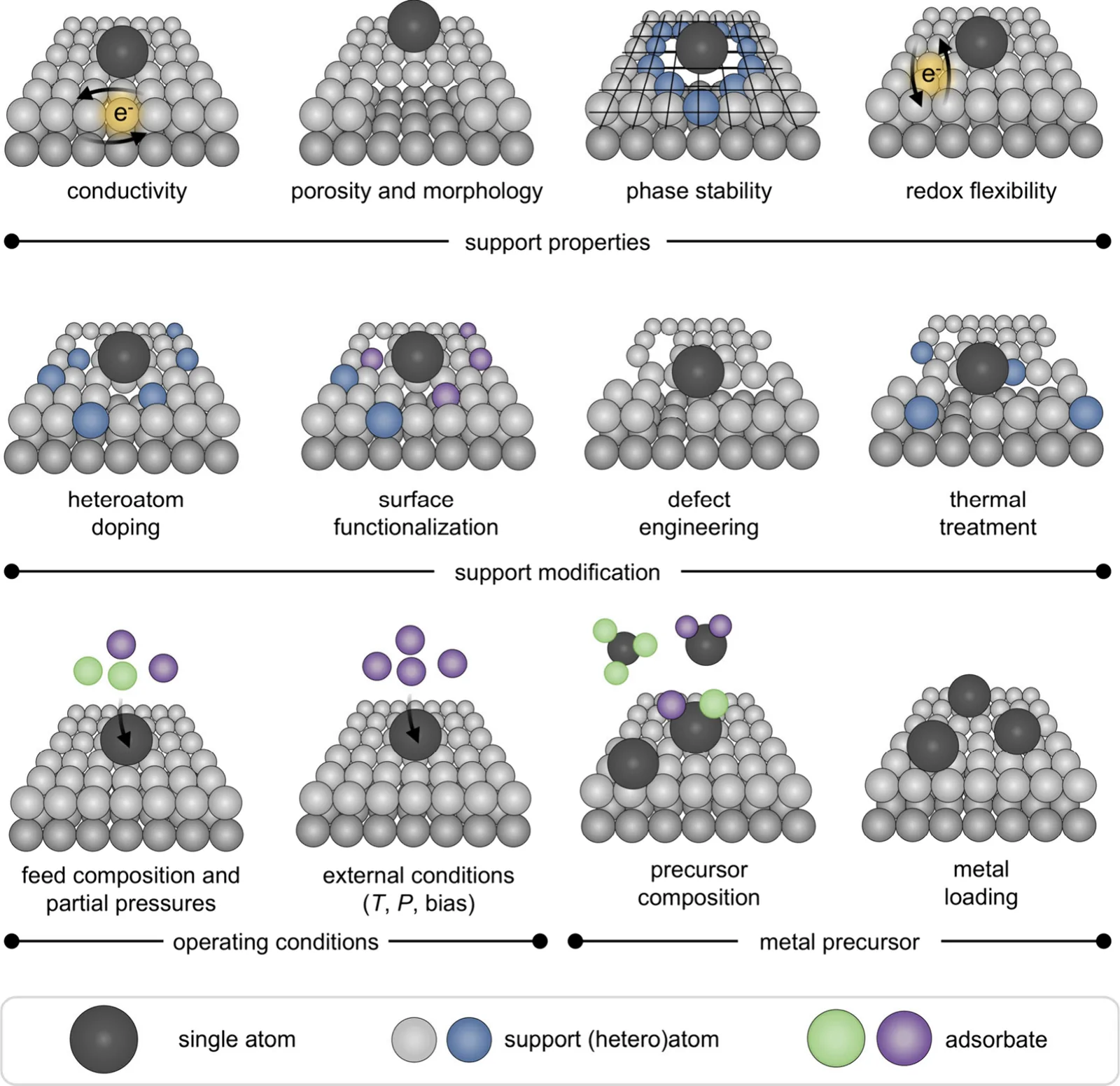
Overview of the key experimental design parameters governing the accessible mechanistic space, including cooperative reactivity at metal–support and metal–metal interfaces, as well as the structural evolution of active sites.

Importantly, not all structural and electronic dynamics observed under reaction conditions correspond to the adaptive coordination discussed above. Transformations with similar atomistic signatures can have fundamentally different implications depending on their reversibility and time scale. When the reversible coordination changes associated with catalytic turnover are no longer fully recovered, the local environment progressively evolves toward a different coordination landscape. This may occur because reaction conditions drive the catalyst toward a new structural equilibrium or because kinetically slow processes persist beyond the time scale of catalytic turnover.^239^ These persistent transformations underpin the structural evolution processes discussed in Section 5.2.

#### 5.1.2 Cooperative Reactivity at the Metal–Support Interface

In many SACs, catalytic roles extend beyond the metal center itself. The surrounding environment shapes catalysis across multiple hierarchical levels, from defining the framework within which elementary steps occur to participating directly in the reaction pathway and ultimately to redefining the active site itself through the emergence of specific interfacial motifs (Figure 21a).^73,260,261^ In these cases, the support acts in concert with the metal center to shape catalytic turnover enabled by adaptive coordination at the active site.

**Figure 21:**
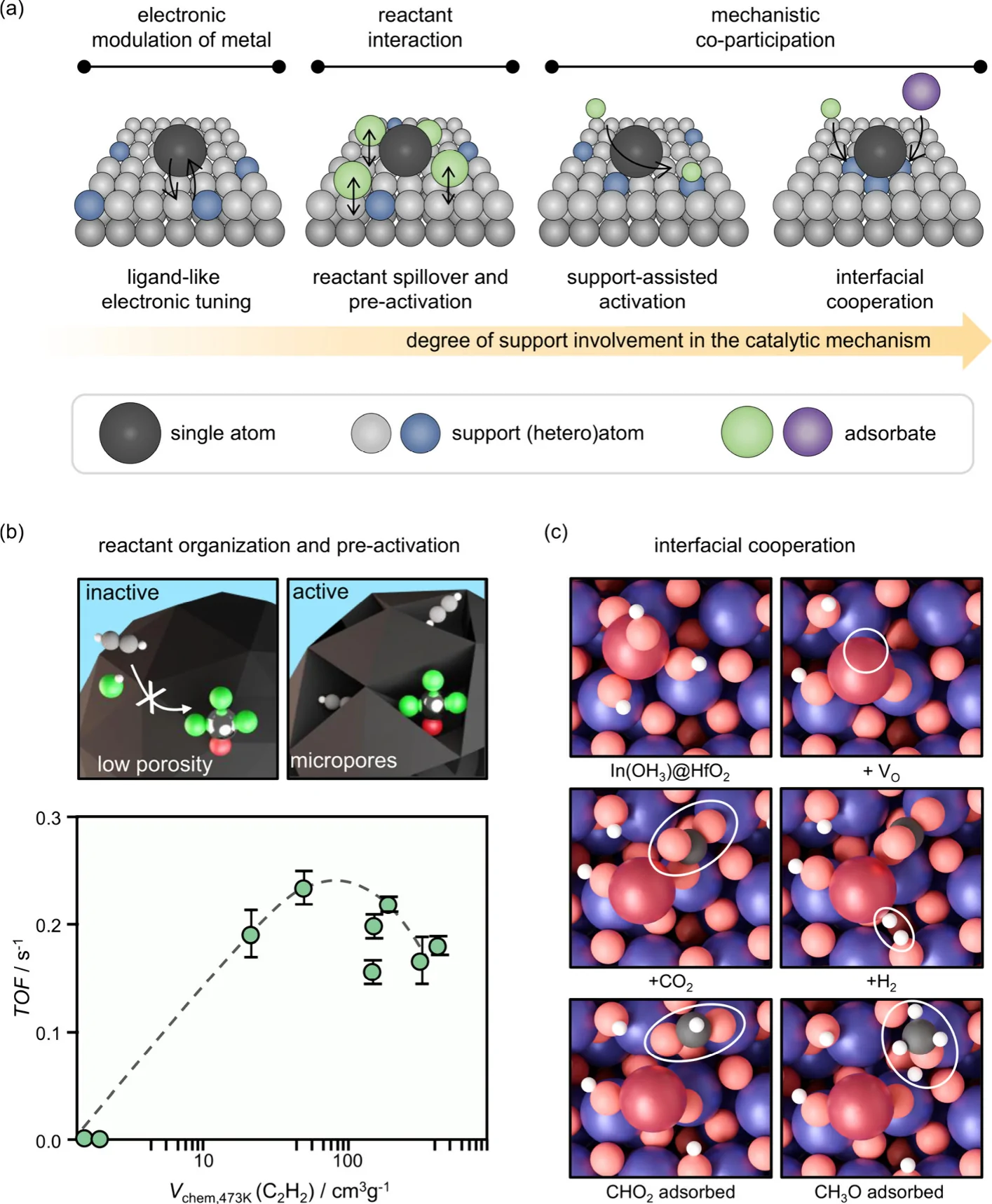
(a) Increasing degrees of support involvement in catalytic processes. (b) Correlation between acetylene hydrochlorination activity and acetylene adsorption capacity in Pt_1_/AC SAC. (c) Cooperative mechanism of CO_2_ hydrogenation at the interface of indium single atoms stabilized on monoclinic hafnium oxide. Part (b) adapted rom ref 265, licensed under CC BY and part (c) adapted from ref 176.

For many catalytic reactions, efficient turnover depends on ensuring that the relevant reactant reaches the active site with sufficient local concentration and in a favorable configuration for the reaction while avoiding excessive site blocking or diffusion limitations. In these cases, the catalytic performance can be improved by selecting supports that adsorb, retain, and orient substrates in the vicinity of metal sites, thereby influencing local concentrations and residence times.^262–264^ Such behavior is conceptually analogous to secondary sphere interactions in molecular catalysis and enzymatic systems, where surrounding residues stabilize key states along the reaction pathway. In acetylene hydrochlorination, for example, all successful SAC formulations rely on activated carbon (AC) supports, where confinement of acetylene within micropores creates a local reservoir that regulates its coverage and availability near the active site, with appreciable catalytic activity only achieved once a threshold volume of acetylene-accessible micropores is established (Figure 21b).^265,266^

Similarly, when reactive intermediates generated at the metal center must participate in subsequent elementary steps elsewhere on the surface, supports capable of sustaining spillover processes become advantageous. Hydrogen spillover from isolated metal atoms into adjacent oxide or carbon domains, for example, redistributes hydrogen across the interface and can enable hydrogenation reactions over extended surface regions.^267^ Similar migration processes involving oxygen or protonic species have been found in a variety of thermal and electrochemical catalytic systems.^268–270^

Many catalytic transformations require complementary chemical functionalities that cannot be efficiently supplied by an isolated metal center alone. In such cases, these functions are distributed across the metal–support interface, where they operate cooperatively. Neighboring acid–base sites, heteroatoms, or redox-active species can act together with the metal center to enable bifunctional activation pathways.^271,272^ NC supports are known to facilitate heterolytic hydrogen activation across metal–nitrogen motifs,^273^ while reducible oxides such as ceria can supply lattice oxygen or hydroxyl species, with oxygen vacancies serving as adsorption and activation sites for reactants and reactive intermediates in redox-coupled steps.^274,275^ In these cases, the catalytic cycle proceeds through cooperative elementary steps that involve both the metal center and the surrounding matrix.

Some catalytic transformations arise from coupled electronic and geometric interactions that extend across the metal–support interface. Engineering these systems therefore requires treating the interface, rather than either component in isolation, as the primary design variable. For example, indium single atoms anchored on monoclinic zirconia or hafnia combine the hydrogen-splitting ability of indium with the wide band gap and dielectric properties of the oxide supports, enabling stabilization of frustrated Lewis pairs composed of hydride and proton species at the metal–oxide interface. These interfacial motifs facilitate heterolytic hydrogen activation and have been shown to promote carbon dioxide hydrogenation to methanol with high efficiency.^176^ Such dynamically formed sites can become integral components of the catalytic cycle, linking the reactivity of active metal centers to the evolving chemistry of the surrounding lattice (Figure 21c).

These examples highlight that catalytic function in SACs often emerges from cooperative interactions at the metal–support interface, where the surrounding matrix actively shapes the reaction coordinate. At this level, reactivity is governed by structural parameters of both the support and the metal center: the support defines the chemical and transport environment through its functionalization, porosity, and defect landscape, while the metal precursor and content determine the nature and distribution of active sites (Figure 20). Together, these factors define the interfacial coordination environments accessible under the working chemical potential. Accordingly, support design should extend beyond stabilizing isolated metal atoms and instead be guided by the specific mechanistic role that the support is intended to perform.

In this context, first-principles methods like DFT provide a framework for discriminating between competing interfacial configurations and evaluating their associated reaction pathways directly at the interface.^176,266^ This description can be further extended using MLIPs,^276,277^ which enable sampling of interfacial restructuring, defect formation, and adsorbate-induced transformations over the length and time scales relevant to catalysis. Crucially, these approaches must be integrated with operando characterization, as the increasing experimental ability to resolve interfacial structures under working conditions provides the necessary reference to identify which configurations are truly active. This combined framework points toward a more realistic and predictive description of metal–support cooperativity.

#### 5.1.3 Cooperative Reactivity between Metal Centers

Cooperative effects in SACs are not limited to metal–support interactions but can also emerge between neighboring metal centers.^56,260,278,279^ The most direct strategy to engineer such interactions is by introducing a second atomically dispersed metal center during catalyst synthesis. When this neighboring center is a different element, the resulting materials are classified as dual-atom catalysts (DAC),^280^ spanning a wide range of structural configurations defined by metal identity, coordination motifs, intersite distances, and connectivity. Cooperative interactions, however, are not restricted to heterometallic systems. Neighboring atoms of the same element may likewise interact when stabilized in sufficiently close proximity, as exemplified by the recently introduced geminal atom catalysts (GAC), in which adjacent identical metal atoms are deliberately anchored at neighboring sites.^174^ More generally, cooperative effects may also arise in conventional SACs when neighboring isolated sites become sufficiently close to interact through the support.

The discussion below is organized according to the different modes of cooperative reactivity that can be established among neighboring local environments. Each mode reflects a distinct way in which neighboring sites influence catalytic function and therefore gives rise to different design opportunities (Figure 22a).^103^

**Figure 22:**
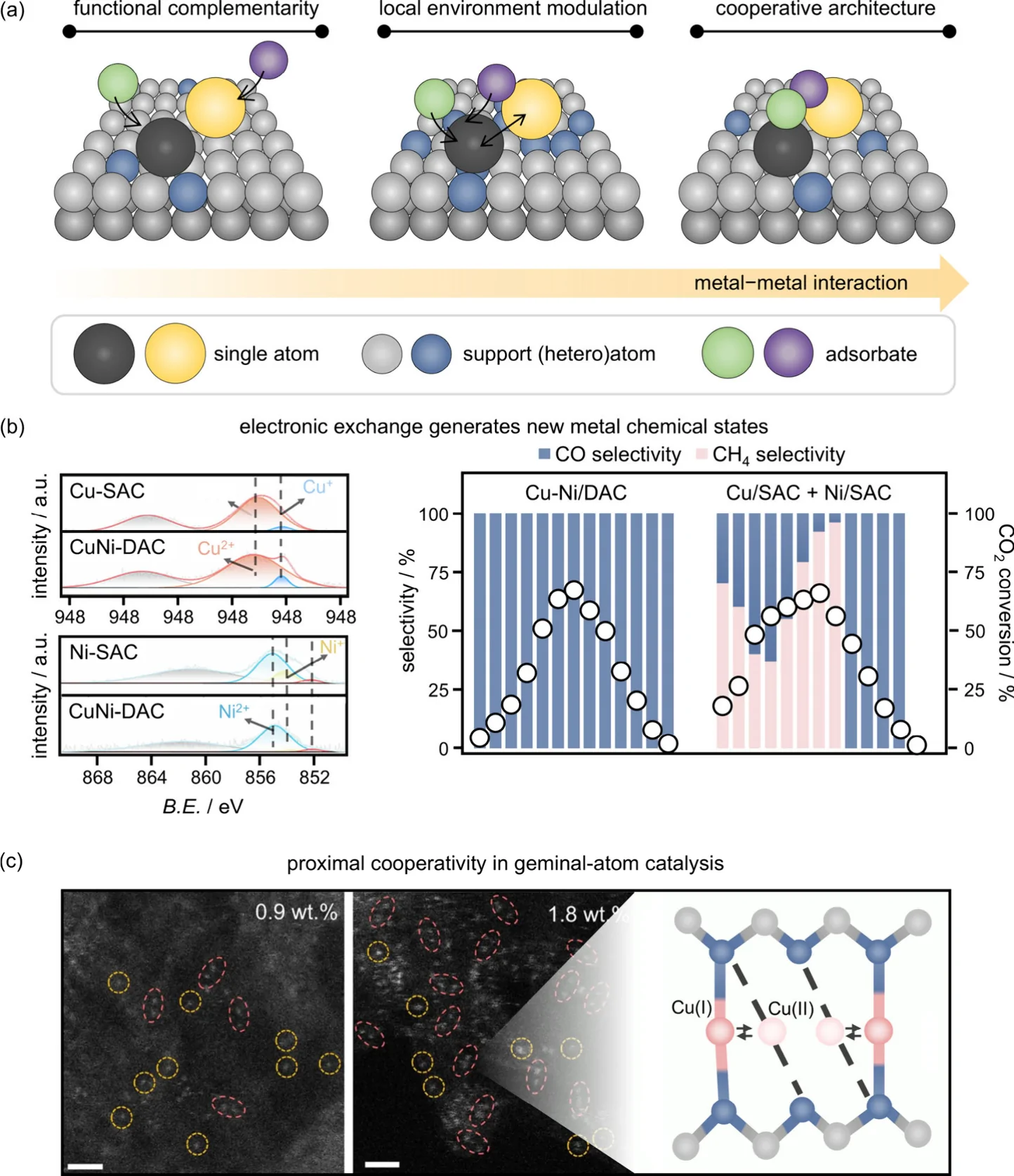
(a) Distinct modes of cooperativity between neighboring metal centers. (b) Cu 2*p* and Ni 2*p* XPS spectra of Cu_1_/NC and Ni_1_/NC SACs and the bimetallic Cu_1_–Ni_1_/NC DAC, along with the corresponding performance in the reverse water gas shift reaction. (c) TEM images of Cu_1_/PCN samples at varying Cu contents, highlighting isolated Cu atoms (yellow circles) and geminal Cu sites (red circles), together with a schematic depiction of their dynamic coordination under reaction conditions. Scale bar, 0.5 nm. Part (b) adapted from ref 291, licensed under CC BY-NC and part (c) adapted from ref 174.

The most intuitive cooperative strategy is to distribute complementary elementary steps across neighboring local environments by introducing a secondary metal center with catalytic functionality unavailable at the primary active site. For example, adsorption and pyrolysis of a bimetallic organometallic precursor on TiO_2_ under argon generates an Ir_1_–Mo_1_/TiO_2_ DAC comprising isolated iridium and molybdenum centers, where hydrogen is activated at the iridium site whereas the remaining catalytic functions are fulfilled by the adjacent molybdenum center.^172^

An alternative strategy is to exploit a neighboring metal center to tune the local environment of an existing active site rather than introducing complementary catalytic functionality. Instead of participating directly in the reaction, the secondary metal reshapes the intrinsic reactivity of the primary site by modifying its coordination environment, electronic structure, or speciation. This mode of interaction is conceptually analogous to promotion in conventional heterogeneous catalysis, except that the spatial and electronic relationship between promoter and active center can, in favorable cases, be resolved at the atomic scale. Depending on the degree of association, the secondary component may act remotely or become an integral part of the local environment of the primary site. At the most indirect level, neighboring metal centers influence one another during catalyst synthesis by altering the local environments ultimately adopted by otherwise isolated sites, even if the resulting sites remain spatially and electronically isolated. For example, in Ru_1_–Pt_1_/NC DAC, platinum and ruthenium remain isolated but adopt coordination environments distinct from those in monometallic catalysts or physical mixtures, reflecting interactions between precursors during formation. This leads to favorable configurations that enhance hydrogen formation rates in the hydrogen evolution reaction, attributed to the generation of more active ruthenium sites.^281^ In such systems, distinguishing cooperation from promotion is not always straightforward. The presence of two atomically dispersed elements does not establish that both participate directly in the catalytic cycle: one center may instead modify the coordination, electronic structure, speciation, or population of the catalytically active environment. Establishing the role of each component therefore requires mechanistic evidence beyond the structural identification of neighboring sites.

The local environment of an active site can also be tuned through electronic communication with a neighboring metal center, allowing the adsorption energetics of the primary site to be modulated without direct participation of the secondary site in the elementary step. Such interactions arise through support-mediated charge redistribution, electron delocalization, or spin polarization and do not require direct metal–metal bonding.^282,283^ Their strength depends on the efficiency of electronic coupling, which is governed by factors such as intersite distance and orbital overlap.^284–288^ For example, Co@N_4_ sites have been proposed to govern oxygen activation, while adjacent Ni@N_4_ sites, although not directly involved in bond activation, primarily tune the electronic structure of cobalt, weakening *OH adsorption while enhancing *H binding, thereby accelerating both oxygen reduction reaction and hydrogen evolution reaction kinetics.^289^

Electronic coupling can be further enhanced by decreasing the intersite distance or by introducing bridging coordination motifs that facilitate charge redistribution between neighboring local environments. Even identical metal centers may therefore cooperate when sufficiently close: in an Fe_1_/NC SAC, reducing the average Fe–Fe separation below ∼ 1.2 nm modified the electronic structure of neighboring iron sites and enhances the intrinsic oxygen reduction activity.^290^ In many systems, this coupling is mediated by bridging coordination motifs (e.g., M–N–M or M–O–M), where the linking atoms actively redistribute electron density and modify the local coordination environment, rather than simply stabilizing the metal centers. For example, in Cu_1_–Ni_1_/NC DAC, a nitrogen-bridged Cu–Ni motif induces charge polarization, rendering nickel electron-deficient and copper electron-rich, as evidenced by the shifts in the Cu 2*p* and Ni 2*p* XPS spectra (Figure 22b). This electronic asymmetry enables cooperative catalysis, where nickel promotes carbon dioxide activation while copper facilitates carbon monoxide desorption, suppressing overhydrogenation.^291^

A distinct design opportunity arises when sufficiently short intersite distances can be established within a precisely defined architecture. Under these conditions, stronger forms of cooperation can emerge between identical metal centers, giving rise to GAC, where adjacent single atoms are stabilized at defined distances (∼4 Å) within structured supports such as polymeric carbon nitride (PCN). Increasing metal content promotes the formation of neighboring geminal sites while maintaining atomic dispersion, enabling dynamic metal–metal interactions under reaction conditions that unlock cooperative pathways inaccessible to isolated sites (Figure 22c). In particular, simultaneous adsorption at neighboring sites enables bridge-coupling mechanisms in C–X bond formation, where reactivity emerges from their coordinated action within a defined geometry rather than intrinsic differences between sites.^174^ Achieving such configurations requires support architectures capable of precisely controlling the relative positioning of neighboring metal centers, together with careful optimization of metal content to maximize the formation of paired sites while avoiding aggregation.

Overall, these examples demonstrate that catalytic function in SACs can emerge from collective behavior across multiple metal centers. At this level, reactivity is governed by structural parameters that control both the spatial and electronic coupling between sites: the support defines the arrangement of anchoring sites and the electronic communication between them, while synthesis-dependent factors such as metal precursor chemistry and metal content determine the site density and distribution. Together, these parameters set the intersite distances and electronic interactions that define the cooperative configurations accessible under working conditions (Figure 20).

SAC, DAC, and GAC should be understood as structural descriptions of the atomically dispersed metal component rather than of the catalytic function. Particularly in the case of DAC, a single designation encompasses a wide range of architectures differing in metal identity, intersite distance, connectivity, and electronic communication, and therefore accommodating fundamentally different modes of cooperation. Likewise, the designation of SAC refers only to the presence of isolated metal atoms and does not preclude cooperative interactions between neighboring local environments. Accordingly, the functional catalytic unit may not correspond to an individual local environment but to the interactions established between neighboring local environments, a distinction not captured by the conventional nomenclature. Cooperative function should therefore be considered independently of catalyst architecture. Catalyst design should begin by identifying the cooperative function required for a given elementary step, with the metal components and catalyst architecture subsequently selected as the structural frameworks best suited to establish the corresponding cooperative local environment.

Achieving control over these cooperative configurations therefore relies on the precise regulation of metal speciation during synthesis and under reaction conditions. This requires resolving the distribution of isolated and interacting sites, as well as their evolution under the working chemical potential, which cannot be captured by a single technique: HAADF-STEM and EXAFS provide information on site distribution and coordination, while DFT is required to resolve electronic coupling and discriminate between competing cooperative mechanisms.^281^ In this context, cooperative reactivity must be understood not as a static property of a given structure but as the outcome of a distribution of accessible configurations, whose population is continuously selected by the reaction environment. This dynamic selection of local environments is the focus of this section.

### 5.2 Structural Evolution of the Local Environment

Under reaction conditions, the local environment of an SAC is governed by a chemical potential distinct from that during synthesis. Variations in reaction composition, temperature, pressure, or applied bias reshape the thermodynamic landscape of the coordination framework, driving its reorganization toward the most stable configuration under operating conditions.^46,47^ Unlike the reversible coordination changes associated with catalytic turnover discussed in Section 5.1.1, these transformations persist beyond individual catalytic events and progressively alter the identity and population of the working sites, thereby redefining the catalytically relevant local environment (Figure 23).^292^

**Figure 23:**
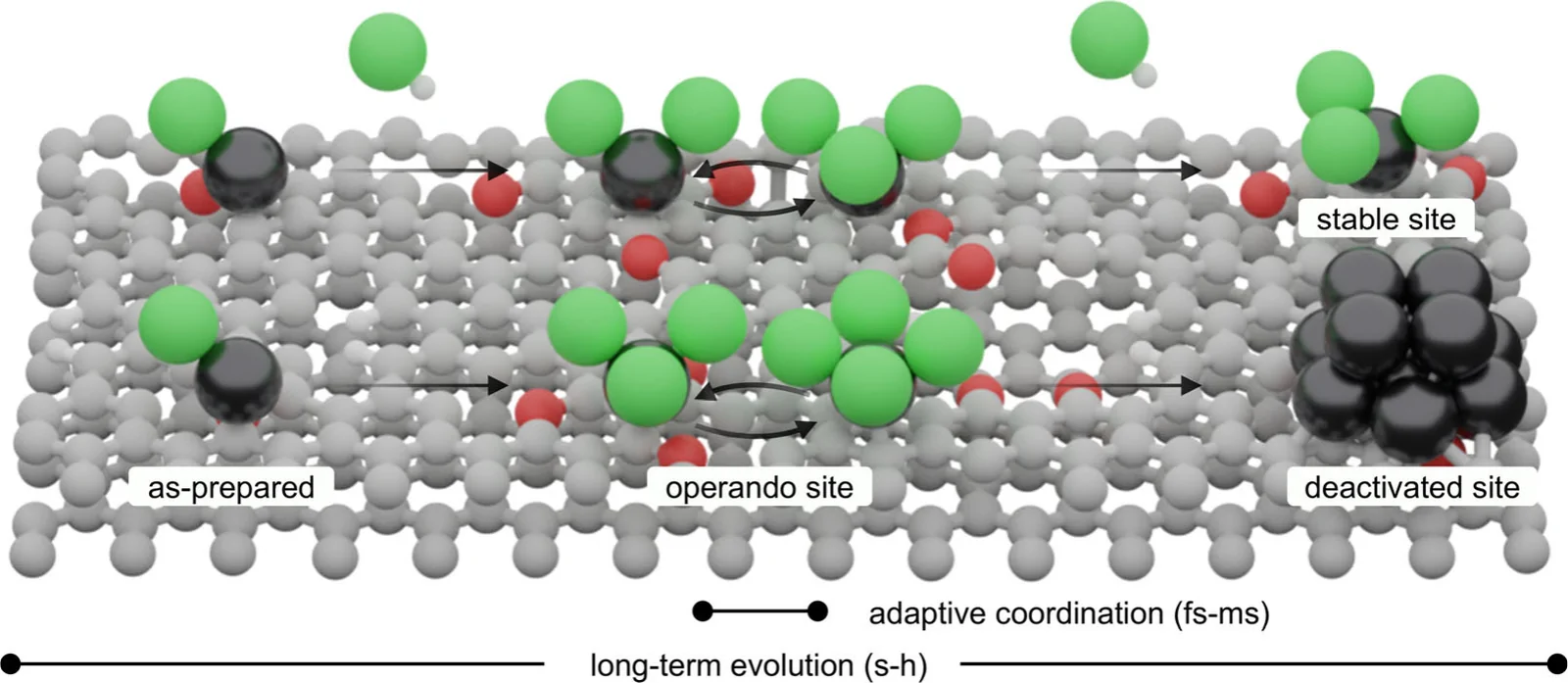
Temporal evolution of structural states in SACs, from fast reversible coordination changes to slower restructuring and eventual deactivation.

#### 5.2.1 Transformations of the First Coordination Sphere

The first level of structural evolution concerns the primary coordination sphere of the metal center. The identity and number of coordinating species, whether originating from the anchoring motif, precursor residues, or adsorbed reactants and intermediates, are redefined under the chemical potential imposed by the reaction conditions. This evolution encompasses a range of interconnected processes involving exchange with reactants and intermediates, redox-driven changes in coordination and electronic structure, and variations in anchoring strength that may, in some cases, lead to changes in nuclearity (Figure 24a).^46,47,53,293^

**Figure 24:**
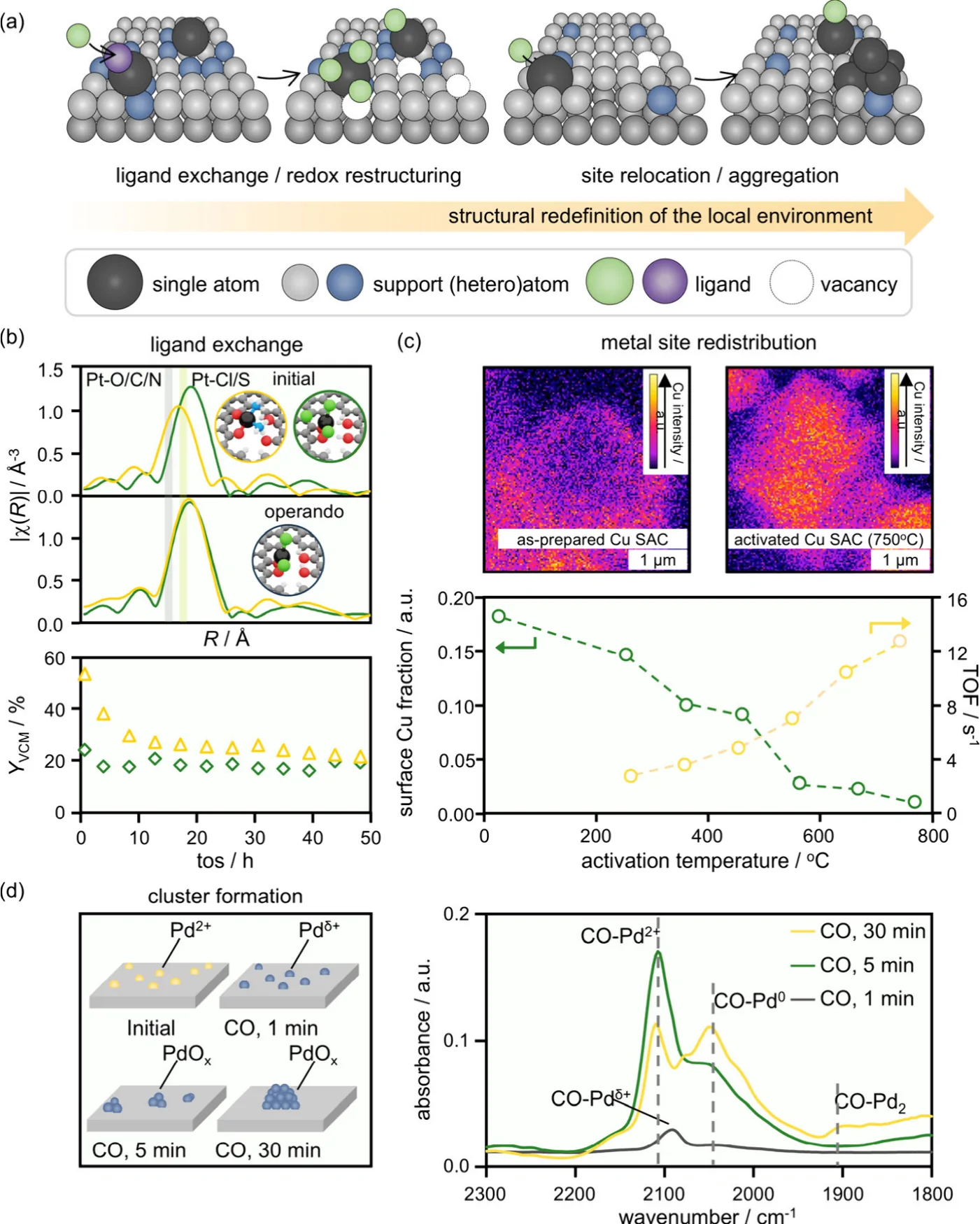
(a) Representative pathways for first-sphere evolution of active sites. (b) Operando XAS tracking of Pt_1_/AC evolution during acetylene hydrochlorination and its catalytic performance. (c) Redistribution of Cu species on silicoaluminophosphate-derived high-silica zeolite, showing changes in surface Cu fraction and the corresponding catalytic activity in NO_*x*_ reduction with NH_3._ (d) CO-induced evolution of Pd_1_/CeO_2_ at room temperature, showing structural changes and time-resolved in situ diffuse reflectance infrared Fourier transform spectroscopy under increasing CO exposure. Parts (b) and (c) adapted from ref 92 and ref 308, respectively. Part (d) adapted with permission from ref 316 (Copyright 2023, Springer Nature).

Ligand exchange often provides the initial pathway for restructuring.^26,294^ Reactants, intermediates, or solvent molecules can displace ligands introduced during synthesis, leading to coordination states that reflect the reaction environment rather than the precursor chemistry. In Pt_1_/AC for acetylene hydrochlorination, for example, distinct precursor-derived geometries converge under reaction conditions to similar Pt@Cl_2/3_O_*x*_ motifs imposed by the hydrogen chloride-rich feed, resulting in convergent catalytic performance despite their different initial structures (Figure 24b).^92^ Similar ligand exchange processes have been reported across SAC systems, including carbon monoxide ligation in oxidation catalysis^239^ and OH coordination under electrochemical conditions,^295–300^ where the resulting coordination states often enhance catalytic performance. This indicates that steady-state ligation is governed by the surrounding chemical potential. As a result, catalyst design must account not only for the initial site definition but also for the coordination states that are selected and stabilized under the operating conditions.

Not all coordination states populated under the reaction conditions contribute to catalytic turnover. Competitive adsorption of strongly binding species can stabilize spectator configurations that suppress activity while maintaining an atomic dispersion. For example, in the aerobic oxidation of thiophenol to disulfide on gold single atoms anchored on carbon nanotubes (Au_1_/CNT), the formation of stable thiolate-bound species prevents oxygen activation, effectively trapping the catalyst in an inactive state despite preserving atomic dispersion.^301^ Similar effects arise from strongly binding sulfur-,^302^ nitrogen-,^303^ or carbon-containing adsorbates,^293^ which can shift adsorption equilibria toward spectator states. Anticipating such competitive coordination is therefore essential for predicting the catalyst performance under realistic feeds.

Redox conditions introduce an additional mode of coordination control by modifying the chemical identity of the metal–support interface itself. Changes in the chemical potential of hydrogen, oxygen, or electrons can drive the formation or removal of lattice oxygen, generate vacancies, and reorganize interfacial bonding motifs, thereby reshaping the first coordination sphere through bond cleavage and formation rather than simple substitution. The oxide-supported systems discussed in Section 4.1 illustrate how these first-sphere transformations are coupled to the initial distribution of adatom, surface-embedded, subsurface, and lattice-incorporated environments. Under reaction conditions, vacancy formation, hydroxylation, reduction, or adsorbate binding can alter the coordination of isolated atoms and determine whether a synthesis-defined environment remains stable or converts into a different local structure. In ZnZrO_*x*_, for example, surface-localized Zn^2+^ sites and nearby oxygen vacancies form productive ensembles for carbon dioxide hydrogenation, whereas deeper incorporation or ZnO segregation reduces the population of accessible methanol-forming environments.^183^ In In-and Co-modified oxide systems, support composition and promoter location similarly determine whether isolated metal–oxygen environments are preserved, transformed into oxygen-deficient active ensembles, or lost through restructuring into less selective phases.

These effects are particularly pronounced on reducible supports, where the lattice actively participates in the redox processes.^304^ For example, in Pt_1_/TiO_2_, the metal dynamically transitions between distinct coordination states under redox conditions, from lattice-incorporated, highly coordinated Pt@O_*x*_ species under oxidizing environments to progressively lower-coordinated, adsorbed, and hydroxylated platinum species under reducing conditions, which become increasingly mobile on the surface.^245^ In some systems, this redox-driven restructuring can go beyond modifying existing coordination environments and instead generate transient active sites under reaction conditions.

In systems exhibiting SMSI, redox conditions can drive continuous evolution of the metal–support interface through reversible encapsulation and re-exposure of single atoms,^232^ migration of support species onto the metal surface (support decoration),^305^ or reaction-induced variations in interfacial bonding and charge transfer.^306^ For example, surface-adsorbed Rh single atoms become encapsulated by the ZrO_2_ support during calcination in air and are re-exposed upon H_2_ reduction,^232^ whereas operando studies of Ru_1_/CeO_2_ revealed that methanation dynamically modifies both Ru–O–Ce interfacial bonding and metal–support charge transfer, resulting in reaction-dependent strengthening or weakening of SMSI depending on the catalyst nuclearity.^306^

When anchoring interactions are sufficiently labile, particularly in the presence of strongly interacting adsorbates, isolated atoms can relocate under reaction conditions and sample different local environments.^181^ In some cases, this mobility can be beneficial, enabling the redistribution of metal centers toward more stable or catalytically relevant sites and the redispersion of nanoparticles into single atoms.^307^ For example, copper atoms on silicoaluminophosphate-derived high silica zeolites migrate toward more confined cages during operation, accompanied by a progressive decrease in the surface copper fraction and a corresponding increase in catalytic activity in NO_*x*_ reduction, illustrating the beneficial role of atom migration in optimizing the distribution of active sites (Figure 24c).^308^

However, increased mobility can also lead to destabilizing pathways. Progressive changes within the first coordination sphere can weaken metal–support interactions, allowing isolated atoms to leave their anchoring sites and, under sufficiently reducing or reactive conditions, undergo changes in nuclearity through aggregation into clusters or nanoparticles.^294,309–315^ Strongly binding adsorbates such as carbon monoxide can further facilitate these processes. In Pd_1_/CeO_2_, carbon monoxide progressively shifts the palladium speciation from predominantly Pd^2+^ toward mixed Pd^2+^/ Pd^0^ states, accompanying the transformation of isolated atoms into highly dispersed PdO_*x*_ subnanoclusters (Figure 24d).^316^ Similarly, in Rh_1_/Fe_3_O_4_, O_2_-derived superoxo species promote the formation of oxidic rhodium clusters that shift the reaction from a Mars–van Krevelen to a Langmuir–Hinshelwood mechanism.^317^ Controlling anchoring strength is therefore essential to balance adaptive mobility while preserving atomic dispersion.

Importantly, many first-sphere transformations can sometimes be reversed upon changes in the reaction environment.^100,318–321^ In Pt_1_/CeO_2_, for instance, high-temperature oxidation generates mobile PtO_2_ species that can migrate and be retrapped at anchoring sites, re-establishing atomic dispersion.^322^ Such reversible redispersion processes illustrate that first-sphere restructuring can proceed in both directions and is governed by the prevailing chemical potential.

Overall, first-sphere evolution can be understood as a coordination-state selection process governed by the relative stability of local environments under the prevailing chemical potential, reflecting not only intrinsic coordination energetics but also the synthetic history and operating conditions (Figure 20). Because the coordination states present under steady-state operation may differ substantially from those inferred ex situ, operando spectroscopies are required to track ligand exchange, adsorbate binding, and redox dynamics. These measurements can be interpreted through DFT simulations that evaluate competitive adsorption and redox-dependent coordination preferences, enabling the identification of the most plausible local environments.^92^ Where relevant, MD simulations can further capture their dynamic interconversion and temporal evolution.^323^

#### 5.2.2 Transformations Beyond the First Coordination Sphere

Structural evolution under reaction conditions extends beyond the first coordination sphere, as the surrounding matrix can reorganize and redefine the environment in which isolated metal atoms operate, even when the identity and nuclearity of the site remain unchanged. Despite their distinct structural origins and underlying mechanisms, these transformations can be viewed through a common framework based on how the supporting matrix accommodates the chemical potential imposed by the reaction conditions. The nature and extent of this accommodation are dictated by the intrinsic response of the support, whether it is through redox buffering, structural flexibility, or chemical reorganization. Depending on whether it remains confined to the immediate coordination sphere or propagates into the surrounding support, the catalytically relevant local environment is reshaped over progressively larger spatial scales (Figure 25a).

**Figure 25:**
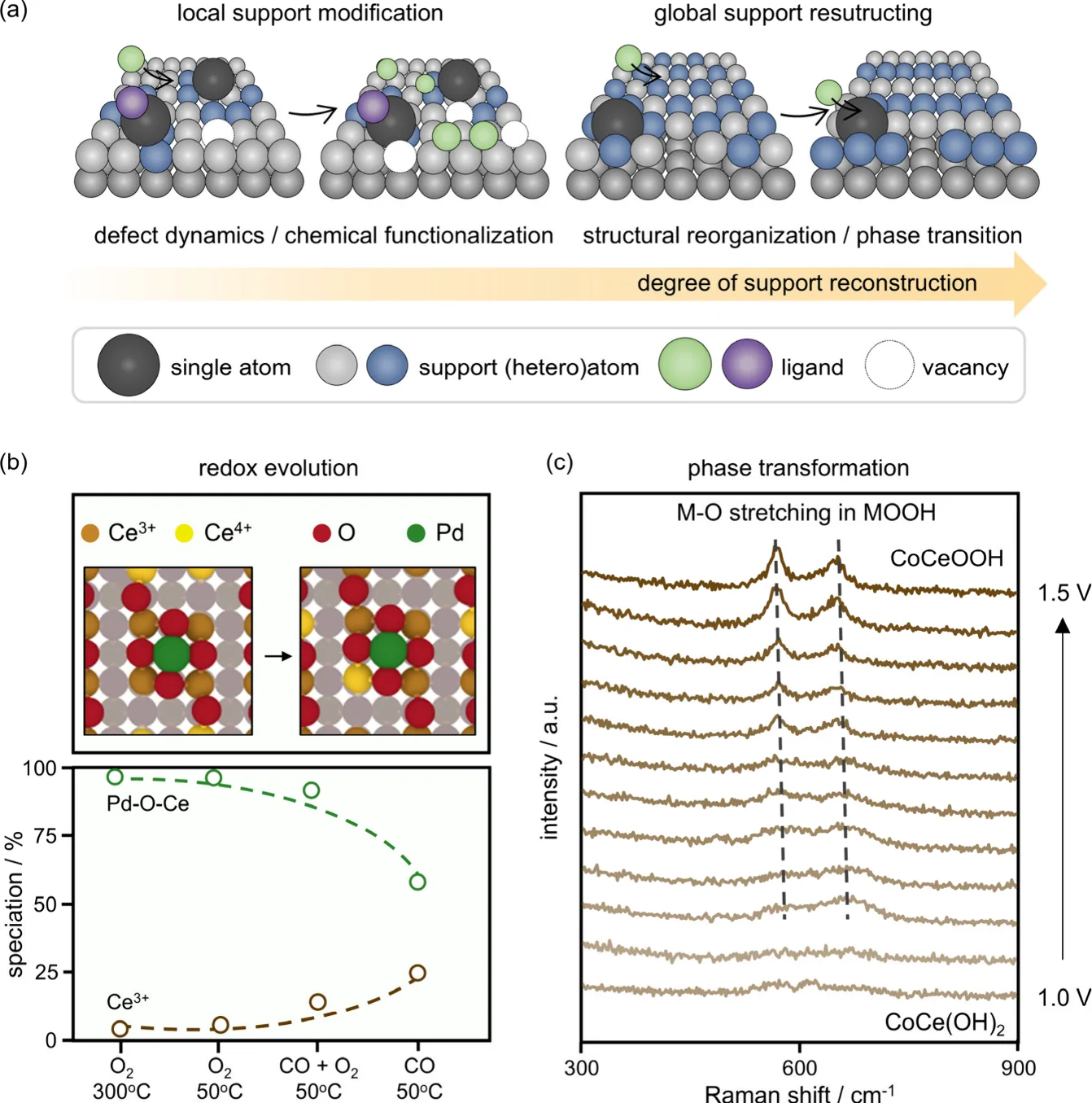
(a) Distinct extents of support reconstruction beyond the first coordination sphere. (b) Evolution of Pd and Ce speciation in Pd_1_/CeO_2_ during CO oxidation, with corresponding changes in relative fractions from operando spectroscopy. (c) Potential-dependent in situ Raman spectra of Ir/CoCe­(OH)_2_ showing the transformation of hydroxide precursors into oxyhydroxide structures during the oxygen evolution reaction. Parts (b) adapted from ref 190, licensed under CC BY-NC and part (c) adapted from ref 327, licensed under CC BY-NC.

In redox-active supports (such as CeO_2_, TiO_2_, FeO_*x*_, CoO_*x*_), this adaptive response is mediated primarily by the continuous redistribution of lattice defects. Fluctuations in oxidation state drive the formation, annihilation, or interconversion of lattice defect motifs, promoting local lattice reconstruction and dynamically reshaping the redox environment surrounding the active site.^324,325^ In Pd_1_/CeO_2_, for instance, increasingly reducing conditions progressively enrich the surrounding ceria lattice in Ce^3+^ species, enhancing oxygen mobility at the metal–support interface. This facilitates reverse oxygen spillover from the support to the isolated palladium atom, suppressing its reduction and thereby preserving the oxidized, atomically dispersed active site (Figure 25b).^190^

In contrast, in supports with limited capacity to accommodate changes through lattice rearrangement, structural adaptation proceeds primarily through the progressive chemical modification of the supporting matrix. The underlying mechanisms depend on the chemical nature of the supports. In carbon-based materials, for example, defect sites, heteroatom functionalities, and unsaturated carbon domains constitute highly reactive centers that accommodate the imposed chemical potential by initiating surface reactions, including protonation, hydroxylation, carbonate formation, and polymerization of intermediates.^326^ These processes progressively reshape the local chemical environment without fundamentally changing the support lattice. In acetylene hydrochlorination, for example, platinum atoms supported on AC may remain atomically dispersed, while adjacent carbon regions polymerize reactive intermediates, generating localized coke deposits that progressively obstruct access to the active site and reduce catalytic activity.^266^ Under more severe conditions, continued chemical modification can ultimately transition into framework degradation. Progressive carbon corrosion increases the density of structural defects, weakens metal anchoring, and eventually promotes metal leaching, surface migration, and redeposition into small metal (oxy)­hydroxide clusters.^118^

At the highest level of structural evolution, the support itself undergoes structural reconstruction or phase transformation, altering the crystallographic framework that hosts the active sites. Such transformations are primarily observed in supports with accessible alternative crystallographic states and become particularly prevalent under electrochemical operation, where the applied potential provides an additional thermodynamic driving force capable of stabilizing reconstructed phases inaccessible under ex situ conditions.^327,328^ A representative example is the conversion of hydroxide precursors into oxyhydroxide structures during oxygen evolution, where the reconstructed phase defines the active environment of embedded metal species (Figure 25c).^327^ In these systems, the as-prepared hydroxide should therefore be regarded as a precatalyst, as the catalytically relevant local environment is only established after reconstruction into the oxyhydroxide phase.

Taken together, these mechanisms highlight that catalyst design beyond the first coordination sphere increasingly focuses on controlling the extended environment in which isolated metal atoms are embedded rather than solely the immediate anchoring motif. The evolution of the matrix, encoded in support topology, redox flexibility, defect chemistry, and surface reactivity, determines how the catalytic environment adapts under working conditions (Figure 20). Engineering these properties constrains matrix reorganization within regimes that preserve spatial isolation while maintaining electronic and structural compatibility with sustained catalytic turnover.

Because these processes involve the evolution of the surrounding matrix rather than changes in the first coordination sphere, operando techniques with temporal and spatial resolution are needed to track defect formation, surface restructuring, and changes in functionality under the reaction conditions. These measurements can be complemented by DFT simulations to probe how matrix-level transformations influence the interfacial energetics and stability. Extending this description to more complex or disordered environments remains challenging, and emerging approaches based on MLIPs offer a possible route to access the time-dependent evolution of the support and its coupling to the metal site over extended length and time scales.^277^ Building on this perspective, the following section addresses how these design variables can be translated into physically meaningful descriptors, enabling their systematic representation and use in predictive frameworks.

## 6 Performance Emerging from Local Environments

Descriptors provide reduced representations that link the structure to performance (activity, selectivity, or stability) and play a central role in catalyst discovery. In single-atom catalysis, however, defining meaningful descriptors is fundamentally a problem of representation: the complexity of local environments must be condensed into experimentally or theoretically accessible structural, energetic, or electronic quantities while preserving the physicochemical features that govern the catalytic performance. This task is complicated by the structural diversity and dynamic behavior of SAC environments under reaction conditions, where multiple coordination states and local configurations may coexist.

This section examines how local catalytic environments can be mapped into descriptor spaces suitable for catalyst analysis and prediction (Section 6.1), how dynamic and heterogeneous environments challenge their definition and interpretation (Section 6.2), and how descriptor spaces can be constructed to explicitly account for this complexity (Section 6.3). Together, these perspectives illustrate how environment-based descriptors can support the development of predictive structure–performance relationships in single-atom catalysis.

### 6.1 Mapping the Local Environment into Descriptor Space

The local catalytic environment can be described at different levels of resolution depending on how it is probed and how it is formalized. Experimental characterization provides ensemble-averaged insight into the structure and chemical state, while theoretical modeling offers site-specific descriptions of bonding, energetics, and electronic structure. Accordingly, structural, chemical, energetic, and electronic descriptors can be understood as complementary projections of the local environment onto measurable or computable quantities. Each captures a specific aspect of how the environment constrains the metal center and, in turn, how it interacts with reactants and intermediates (Table 4, Figure 26).

**Figure 26:**
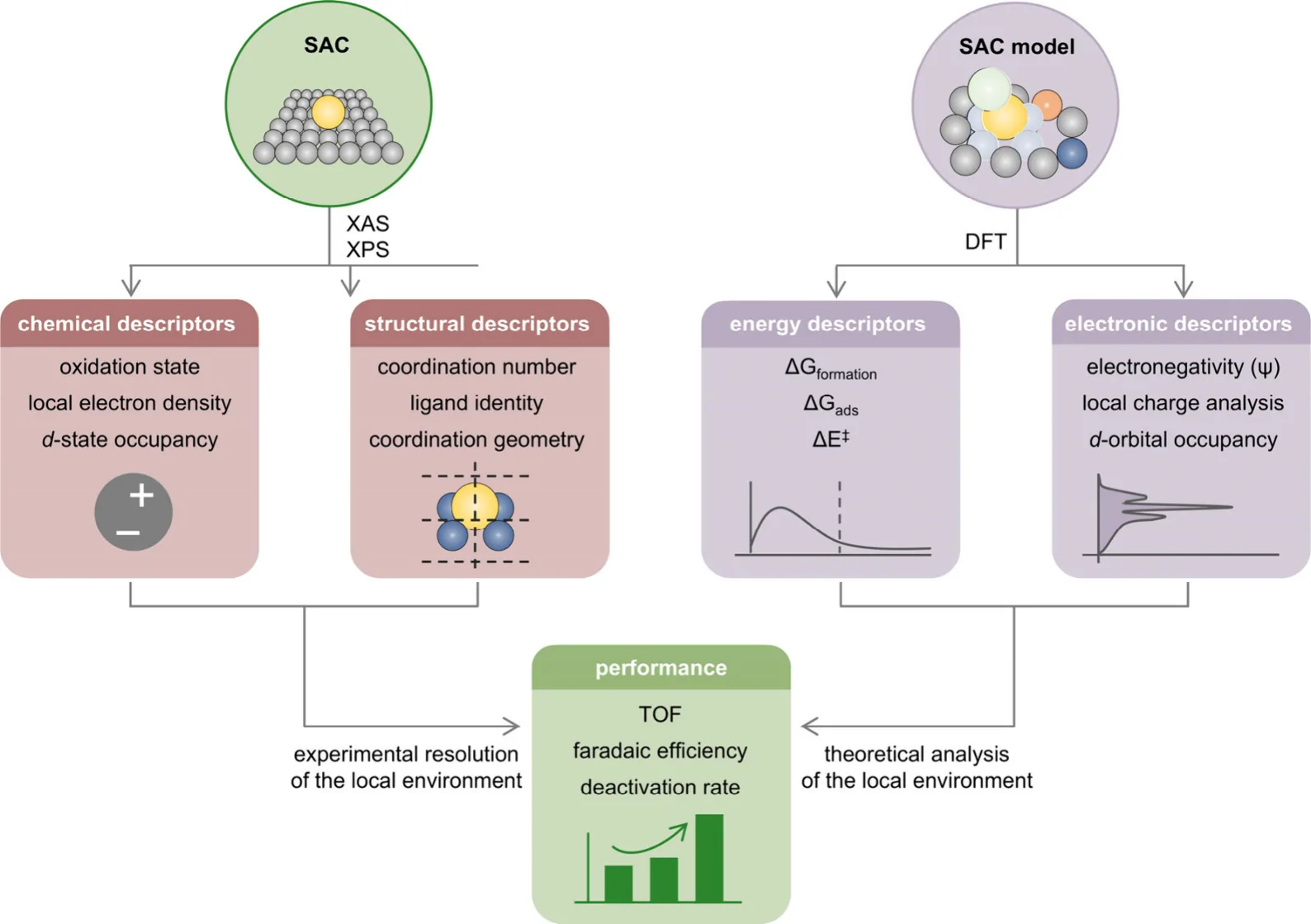
Descriptor construction from local catalytic environments through experimental characterization and theoretical modeling.

**Table 4:** Comparative Assessment of Structural, Electronic, and Energetic Descriptors for SAC

concept	structural descriptors	energetic descriptors	electronic descriptors
physical interpretability	very high, direct correspondence with atomic motifs	moderate, directly linked to catalytic function, but not to its microscopic origin	high, but indirect, reflecting physically meaningful properties rather than explicit features
sensitivity	responsive only to controlled structural perturbations	responsive to the effects of structural and electronic perturbations	responsive to subtle electronic perturbations
transferability	limited to catalyst families with controlled structural variability	high across diverse catalysts sharing a common mechanistic regime	generally high across structurally diverse catalyst families
best suited for	well-defined catalyst families; active-site description, structure–performance relationships	reactions with well-established mechanisms; activity/selectivity prediction and mechanistic analysis	diverse catalyst families; universal comparison and electronic structure–activity relationships

At the most direct level, structural descriptors capture how atoms are arranged around the metal center.^73,321^ These include coordination identity (e.g., M@O_*x*_ versus M@C_*x*_ or M@N_*x*_ bonding environments),^48,60,70,329–331^ coordination number,^50,71^ site topology (e.g., edge versus basal motifs),^332^ bond lengths,^77,333,334^ and the chemical configuration of anchoring motifs such as pyrrolic or pyridinic nitrogen in carbon supports.^335^ Closely related, chemical descriptors reflect how this coordination environment translates into an electronic state of the metal center, often expressed in terms of oxidation state or local electron density.^336,337^ Structural and chemical descriptors are intrinsically coupled: variations in coordination and ligand identity directly determine charge redistribution within the environment. For example, decreasing the coordination of platinum in Pt_1_/Fe_2_O_3_ lowers the oxidation state of platinum and enhances hydrogenation activity without loss of chemoselectivity (Figure 27a).^50^

**Figure 27:**
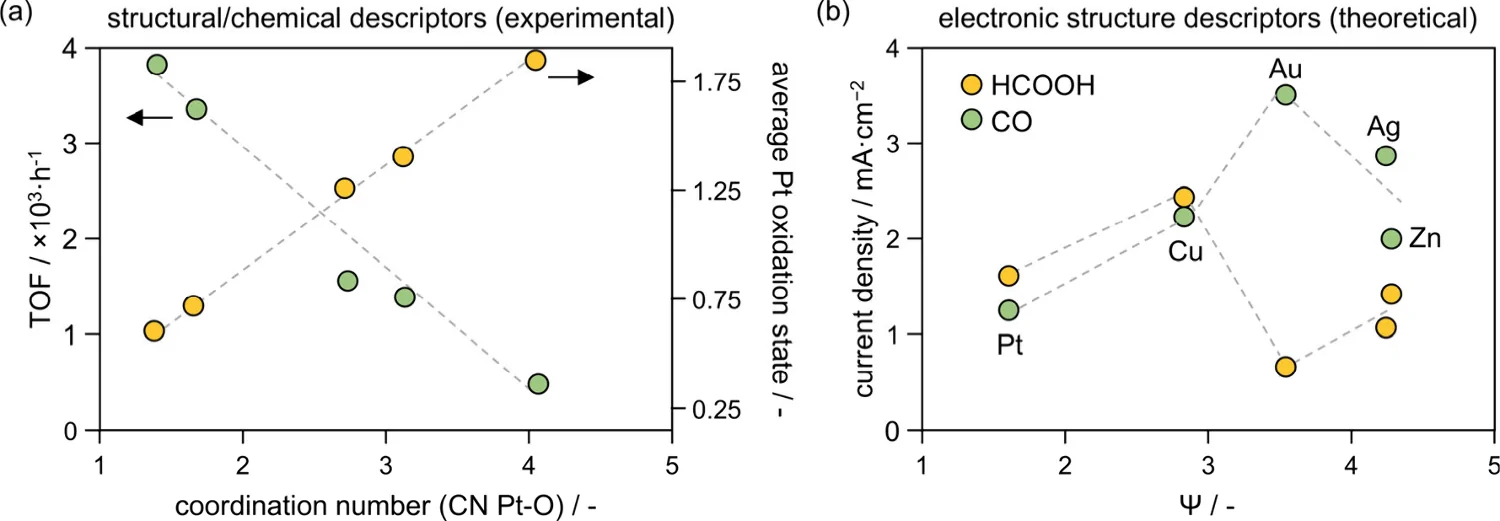
(a) Correlation between coordination number from EXAFS, oxidation state from XANES, and catalytic performance in the chemoselective reduction of 3-nitrostyrene over a Pt_1_/Fe_2_O_3_ SAC. (b) Partial current densities for CO_2_ electroreduction plotted against ψ, composite electronic descriptor derived from valence electron number and electronegativity. Part (a) adapted from ref 50, licensed under CC BY and part (b) adapted from ref 76.

These experimentally accessible descriptors are typically obtained by complementary techniques. XAS provides coordination numbers and bond distances via EXAFS and insights into the electronic structure via XANES. XPS probes the local electronic environment through core-level shifts, while microscopy techniques such as TEM resolve spatial distributions, defect structures, and site topologies.^28^ Structural and chemical descriptors have the highest degree of physical interpretability among all descriptor families because they directly encode chemically meaningful features of the local environment, making them particularly valuable for establishing intuitive structure–performance relationships. However, this direct correspondence comes at the expense of sensitivity, as chemically distinct catalytic environments may exhibit identical structural descriptors despite substantial differences in electronic structure or metal–support interactions. Consequently, their transferability is generally limited to catalyst families with sufficiently controlled structural variability, where the information encoded by a structural descriptor remains uniquely associated with the local environment in which it is intended to represent.

Local environments can also be represented through their energetic response to chemical transformations. Energetic descriptors quantify the thermodynamic and kinetic properties of catalytic processes, including adsorption energies, reaction free energies and activation barriers (Figure 26).^72,73,77,290,321,333,336,338–340^ These descriptors are obtained from atomistic models derived from experimentally informed structures and directly relate to reaction rates and mechanistic preferences. Variations in adsorption energetics across different environments correlate with activity trends, while differences between competing intermediates determine selectivity.^290,341^ Beyond reactivity, energetic descriptors also describe the stability of the catalytic environment itself.^60,95,337,342^ Formation energies of active site motifs, binding energies,^48,72^ diffusion barriers,^337^ and condition-dependent phase behavior determine whether a given environment can form, persist, or transform under reaction conditions.^77,343,344^

Energetic descriptors directly inform on catalytic function but provide only limited information about the physicochemical characteristics of the underlying local environment. Accordingly, they are particularly effective for mechanistic analyses, while remaining highly sensitive to subtle variations in the local environment encoded in atomistic models. This sensitivity also makes descriptor performance relationships considerably less dependent on strict structural control than structural descriptors, allowing correlations to remain valid across a broader range of SAC architectures. Their transferability, however, ultimately relies on mechanistic consistency and may break down when changes in the rate-determining step or other mechanistic features arise, as can occur in systems with substantial differences in support participation, second-shell chemistry, or, more generally, structural modifications that promote alternative reaction pathways.^345^

Building on these theoretical representations, electronic descriptors provide a more compact and often more transferable description of how local environments govern reactivity.^73,321,346,347^ Quantities such as charge density,^48,77,331,333,334,339^ projected density of states, *d*-band center,^60,72,338,339^ *d*-orbital occupancy,^348^ spin or magnetic moments^72,77,290,333,334^ describe how electronic states are distributed and how they interact with adsorbates. Many electronic descriptors are semiempirical quantities that condense multiple aspects of the local environment into a single parameter.^72^ For example, the valence electronegativity (ψ),^349^ defined as a function of valence electron number and electronegativity, correlates with the measured partial current densities for carbon monoxide and formic acid formation across transition metals (Figure 27b).^76^ More broadly, electronic descriptors capture how coordination constraints translate into bond strength, charge transfer, and reaction energetics, including subtle first- and second-shell effects beyond purely geometric descriptions.^350^

Electronic descriptors retain a high degree of physical meaning, because they quantify the electronic factors governing chemical bonding. Their relationship with catalytic behavior, however, is generally less direct than that of structural descriptors, as changes in electronic quantities often integrate multiple structural contributions, whose individual effects cannot be readily disentangled. At the same time, this more fundamental representation makes electronic descriptors among the most transferable descriptor families, enabling meaningful comparisons across structurally diverse catalysts. Their transferability may nevertheless become limited when catalytic behavior emerges from multiple competing electronic effects that cannot be consistently represented by a single electronic quantity.^159^

Importantly, structural, energetic, and electronic descriptors can be used in a complementary manner to progressively refine the representation of the catalytic environment. Machine learning can further expand this framework by enabling the analysis of high-dimensional descriptor spaces to identify the variables most strongly correlated with catalytic performance and combine them into compact, physically interpretable models.^351,352^

### 6.2 Limitations and Challenges in Descriptor-Based Representations

The application of structural, electronic, and energetic descriptors to SACs raises fundamental challenges related to how catalytic environments are represented, interpreted, and compared. The first concern concerns how structural heterogeneity can be represented within a finite descriptor framework. Unlike extended metal surfaces with well-defined crystallographic motifs, SACs can stabilize multiple anchoring configurations on a given support. Defects, heteroatom distributions, surface terminations, and vacancies generate families of local environments that may coexist within a single material.^341^ The extent of this structural variability depends strongly on the support, as discussed in Sections 4.1 and 4.2. Carbon supports exhibit significant heterogeneity arising from defects, functional groups, and edge sites, leading to ensembles of related coordination environments.^353^ In contrast, oxide and nitride supports are constrained by crystallographic terminations and lattice defects, which restrict accessible motifs but introduce strong coupling between the local structure and electronic state. For example, in reducible oxides such as titania or ceria, metal atoms can anchor at different terminations or vacancy sites, where the coordination and electronic structure are intrinsically linked.^85^ The resulting descriptor challenge lies in establishing the appropriate representation of structural heterogeneity within a finite descriptor framework for which no consensus currently exists between average structures, representative local environments, and structural distributions.

A second, closely related challenge arises from the dynamic and cooperative nature of the local environments. As discussed in Section 5, local environments in SACs can evolve under reaction conditions through redox processes, ligand exchange, defect migration, or restructuring of the surrounding matrix, while catalytic behavior often emerges from the coupled response of multiple environmental components. Defining adequate descriptors therefore requires determining whether catalytic behavior should be represented by a sequence of evolving states, an effective time-averaged representation, or a single reaction-relevant configuration while preserving the contribution of cooperative interactions.

The heterogeneous and dynamic behavior of SACs further limits the transferability of descriptors. Quantities that correlate with performance within one class of materials do not necessarily retain predictive power across different supports or metals as the same descriptor may encode different physical meanings depending on the underlying interactions. This is particularly evident under electrochemical conditions, where changes in electrolyte composition, interfacial electric fields, or solvation effects continuously reshape the local catalytic environment, making descriptor values inherently dependent on the operating conditions at which they are defined.

Although these challenges are increasingly recognized, they remain largely unresolved, and no descriptor or descriptor framework yet provides a universally applicable representation of realistic SAC environments. Most existing descriptors are still formulated from static, single-site representations of the active center, while structural heterogeneity, dynamic evolution, and cooperative interactions are typically acknowledged but are only rarely formalized within the descriptor itself. As a result, descriptor representations often collapse multiple catalytically distinct environments into the same descriptor value, obscuring the physicochemical differences that govern their reactivity. This can lead to (i) establishing spurious structure–performance relationships by assigning identical descriptor values to catalytically distinct local environments; (ii) compromising catalyst ranking by making chemically distinct environments appear equivalent in descriptor-based screening; and (iii) directing catalyst design toward reproducing descriptor values rather than engineering the catalytic environments responsible for the desired catalytic response.^159,345,354^

Beyond descriptor formulation, one of the central practical challenges is reconciling the experimental and theoretical representations of the catalytic environment. Experimental observables typically reflect ensemble-averaged properties over distributions of local environments, whereas theoretical descriptors are defined for specific structural configurations. As a result, direct comparison between the two does not necessarily involve the same physical entity, complicating the validation and interpretation of structure–performance relationships.

Finally, activity, selectivity, and stability are often described using separate descriptors despite originating from the same underlying local environment. Activity is typically linked to adsorption and reaction energetics, selectivity to differences between competing pathways, and stability to formation energies or resistance to structural transformations. Treating these aspects independently can obscure their intrinsic coupling, as all three emerge from how the local environment responds to the reaction conditions.

### 6.3 Descriptor Spaces for Dynamic and Heterogeneous Local Environments

Improving descriptor representativeness begins with reconsidering how existing structural, electronic, and energetic descriptors are constructed, measured, and interpreted under the operating conditions. Experimentally, descriptors should increasingly be extracted under operando conditions to probe the catalytically relevant environment, rather than the as-prepared catalyst. Likewise, theoretical descriptors should be evaluated through systematic exploration of catalytically accessible states, rather than being derived from a single optimized structure chosen solely to reproduce ensemble-averaged spectroscopic observables, which may not correspond to a physically representative catalytic environment.^158^ In both cases, descriptor construction should not be restricted to the immediate coordination sphere but should incorporate secondary local environments, including the spatial arrangement of neighboring isolated sites.^128,330^

Beyond this, more advanced strategies seek to move from descriptors defined for individual configurations toward ensemble-based representations in which the properties of multiple accessible states are combined according to their thermodynamic or kinetic relevance. In systems with significant structural variability, thermodynamic approaches provide a natural way to construct ensemble representations without requiring a full mechanistic resolution. In this framework, multiple configurations of the same site are considered, and their contributions are combined through Boltzmann populations, implicitly assuming a quasi-equilibrium distribution of catalytic states.^158^ For example, in Ni_1_/NC catalysts for carbon dioxide electroreduction, Boltzmann-weighted energy spans have been used to account for different hydrogen-covered configurations, capturing how activity and selectivity emerge from their relative populations.^49^

Building on this, kinetic frameworks provide a more explicit description of how different states contribute to the reactivity. Microkinetic models capture the distribution of adsorbed intermediates and competing reaction pathways on a given active site through coverage-dependent kinetics.^355^ However, these models are typically constructed for a single predefined structural model and, therefore, do not explicitly account for the coexistence of multiple distinct active site configurations.

A complementary strategy focuses on resolving the ensemble experimentally. Advances in spectroscopic techniques aim to disentangle the contributions of distinct catalytic environments, providing more detailed insights into the distribution of active sites. For example, site-resolved NMR can deconvolute spectroscopic signatures associated with different coordination motifs, offering experimental constraints on the ensemble of environments present in a catalyst.^55^ However, translating this structural resolution into quantitative contributions to catalytic activity and selectivity remains a major challenge, as measured performance still reflects the collective response of the ensemble.

As efforts increasingly incorporate operando conditions and environmental effects, a growing class of approaches focuses on describing the conditions under which specific active-site configurations are stabilized.^349^ In this framework, the chemical potentials of relevant species like O, H, or CO are used to construct stability diagrams in which different regions of descriptor space correspond to distinct active-site configurations.^292^ An analogous concept is widely used in electrochemistry through Pourbaix diagrams, where potential and pH determine the stability of different surface or bulk states.^344^ In both cases, the descriptor does not represent an intrinsic property of a predefined structure, but rather the external conditions that govern which structure is realized, reflecting a shift from static, configuration-specific descriptions toward defining catalytic environments as condition-dependent states within a thermodynamic space.

This Perspective extends beyond stability to catalytic activity. In Au_1_/TiO_2_-catalyzed carbon monoxide oxidation, microkinetic modeling shows that reaction energetics and dominant mechanisms depend explicitly on temperature, oxygen partial pressure, and surface oxygen coverage, with the oxidation state of the support modulating both active site stability and reaction barriers.^356^ Similarly, descriptors based on hydrogen coverage and lifetime under reaction conditions have been used to capture the availability of reactive hydrogen, linking its dynamic supply and consumption directly to catalytic selectivity rather than relying solely on static adsorption energies (Figure 28a).^357^ As a result, activity descriptors become inherently state-dependent, and catalytic performance emerges from the interplay between structure and environment under reaction conditions.

**Figure 28:**
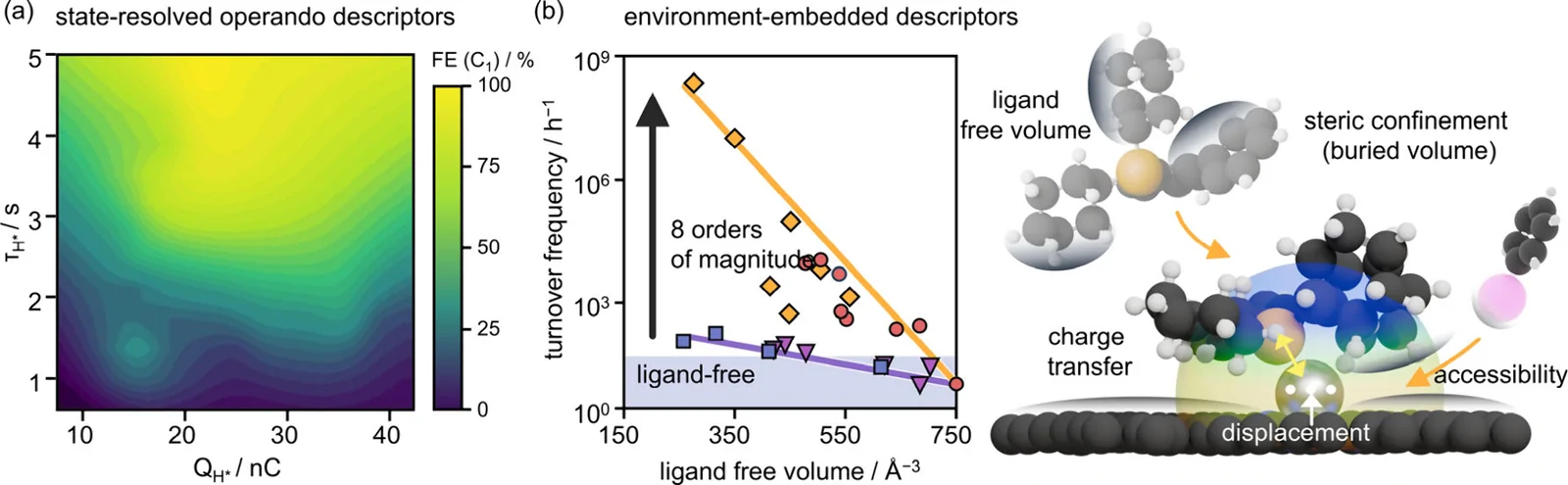
(a) Contour plot of Faradaic efficiency toward C_1_ products in CO_2_ electroreduction as a function of surface hydrogen coverage (Q_H*_) and lifetime (τ_H*_). (b) Ligand effects in Sonogashira coupling over Pd_1_/NC, highlighting the combined roles of steric confinement (buried volume), electronic interactions, and accessibility in determining turnover frequency. Part (a) adapted from ref 357 and part (b) adapted from ref 358, licensed under CC BY.

More generally, activity can also be described by incorporating structural flexibility and spatial constraints into the descriptor formulations. For example, in Pd_1_/NC for cross-coupling reactions, performance is governed by geometric descriptors such as the average ligand volume, deformation energy, and buried volume, which together reflect a support-constrained coordination environment in which activity arises from ligand-induced displacement and exposure of the metal center rather than from modulation of its electronic structure. (Figure 28b).^358^

Explicit dynamic effects are most naturally incorporated through theoretical approaches, where the evolution of the catalytic system can be followed under the reaction conditions. Rather than being fully formalized, descriptors often capture the key dynamic features that are relevant to the mechanism. In this context, descriptors based on the electronic response of the active site, such as changes in the spin moment upon adsorption in Fe_1_/C_2_N, link electronic rearrangement directly to activity.^359^ Similarly, in Cu_1_/TiO_2_ photocatalysts, time-dependent electronic states identified through nonadiabatic molecular dynamics govern carrier lifetimes and reactivity.^360^ Together, these approaches show that descriptors derived from an evolving electronic structure can effectively capture dynamic catalytic behavior.

Finally, progress toward unified activity-selectivity-stability maps remains limited, although multiresponse surfaces represent an important step in this direction. In these approaches, activity, catalyst state, and stability emerge consistently from the same kinetic model rather than from separate correlations. For example, in carbon monoxide oxidation on different SACs supported on alumina, adsorption energies define a map in which different regions correspond to distinct reaction pathways, rate-determining steps, and catalyst states, while stability is incorporated through diffusion-related metrics.^361^ The key advance is therefore the ability to translate a single mechanistic model into a continuous map of catalytic behavior across materials and conditions.

Ultimately, descriptor construction in single-atom catalysis does more than correlate performance with structural or electronic parameters; it defines how the catalytic environment itself is represented, whether as a single site, an ensemble, or a state-dependent configuration. Advancing predictive SAC design will therefore require descriptors that explicitly incorporate structural heterogeneity and dynamic behavior within unified mappings of catalytic function.

## 7 Representative Environment-Resolved Case Studies

The preceding sections established a framework for describing catalytic local environments across their structural and electronic characteristics, formation, characterization, catalytic function, dynamics, evolution, and governing descriptors. Here, these dimensions are brought together through integrated case studies of three representative SAC platforms: Pt_1_/CeO_2_ for thermocatalytic CO oxidation,^85,362–366^ Ni_1_/NC for electrochemical CO_2_ reduction,^49,367–370^ and Pt_1_/C_3_N_4_ for photocatalytic H_2_ evolution.^87,259,371–373^ These systems span distinct catalytic regimes while providing sufficiently mature experimental and theoretical understanding for an environment-resolved analysis. Each also illustrates a different source of local-environment complexity: metal–support interactions, defect chemistry, and restructuring in Pt_1_/CeO_2_; coordination heterogeneity, carbon-support chemistry, and potential-dependent interfacial organization in Ni_1_/NC; and the interplay between local coordination, semiconductor properties, and photoinduced charge transfer in Pt_1_/C_3_N_4_.

Each system is examined across the catalyst lifecycle by using the same environment-resolved framework. The analysis first identifies the accessible primary, secondary, and extended environments (Table 5) and then examines how the support and synthesis- and operation-dependent conditions govern their formation and distribution (Table 6). The complementary experimental and computational approaches available to resolve these environments and their evolution under operating conditions are subsequently assessed (Table 7), followed by their structural and electronic dynamics, catalytic function, and longer-term evolution (Table 8). Finally, the structural, energetic, and electronic descriptors that connect local environment characteristics with catalytic behavior are compared across the three systems (Table 9). Together, these case studies put the framework into practice and provide a common basis for describing and comparing chemically distinct SACs without reducing their behavior to a single structural motif or static active-site representation.

**Table 5:** Accessible Primary, Secondary, and Extended Local Environments in Representative SAC Systems Across Thermocatalytic, Electrocatalytic, and Photocatalytic Regimes

environmental level	Pt_1_/CeO_2_ for thermal CO oxidation	Ni_1_/NC for electro CO_2_ reduction	Pt_1_/C_3_N_4_ for photocatalytic HER oxidation
primary environment	• predominantly O-coordinated Pt environments	• Ni can occupy chemically diverse environments, involving coordination to different N, C, and other heteroatom species	• Pt can occupy several C/N environments in the C_3_N_4_ framework
	• Pt–O coordination number and geometry varying among accessible anchoring configurations	• coordination number and geometry span a broad range, from highly coordinated or square-planar to undercoordinated	• coordination number and geometry vary within this framework-defined space, from saturated to undercoordinated and distorted configurations
	• predominantly cationic Pt (especially Pt^2+^), with other cationic and more reduced states accessible	• predominantly cationic Ni (especially Ni^2+^)	• predominantly cationic Pt (Pt^2+^/Pt^δ+^), with other cationic and more reduced states accessible
secondary environment	• Pt–O–Ce connectivity	• Pt neighboring C/N functionalities and heteroatoms	• N functionality and C–N connectivity
	• proximal O vacancies and Ce^3+^/Ce^4+^-polaron configurations	• proximal vacancies and defects	• proximal N vacancies and framework defects
	• site-specific terrace, step, edge, (sub)surface configurations		
extended environment	• CeO_2_ facet/step topology and longer-range O-vacancy/Ce redox distributions	• carbon domains varying in graphitization, porosity, hybridization, and heteroatom/defect distributions	• C_3_N_4_ regions varying in layering, structural order, connectivity, and longer-range defect distributions
	• lattice flexibility, O mobility, and Ce 4*f* states provide longer-range coupling	• electronic conductivity and electrode–electrolyte interface	• semiconductor electronic structure and conjugation

**Table 6:** Support Constraints and Synthesis- and Operation-Dependent Conditions Governing the Formation and Distribution of Local Environments in Representative SAC Systems

environment influences	Pt_1_/CeO_2_ for thermal CO oxidation	Ni_1_/NC for electro CO_2_ reduction	Pt_1_/C_3_N_4_ for photocatalytic HER oxidation
catalyst-derived	• facet and terrace/step/edge topology	• carbon topology and graphitization/hybridization	• degree of polymerization and layering/exfoliation
	• ceria lattice and available O coordination	• N content/speciation and heteroatom composition	• C/N vacancies
	• reducibility, O mobility, Ce 4*f* electronic structure	• porosity and electronic conductivity	• conjugation, semiconductor electronic structure
condition-dependent	• Pt deposition/incorporation and defect engineering	• N/heteroatom, defect engineering, Ni incorporation	• C_3_N_4_ precursor, defect engineering, Pt incorporation
	• thermal, redox, and hydrothermal conditioning	• postsynthetic treatments	• postsynthetic conditioning and activation
	• hydration/hydroxylation conditions	• electrolyte/solvent composition, pH, ionic strength	• reaction medium and temperature
	• CO/O_2_ atmosphere, temperature, and pressure	• applied potential	• illumination conditions

**Table 7:** Complementary Experimental and Computational Approaches for Resolving Primary, Secondary, and Extended Environments and Their Evolution under Operating Conditions in Representative SAC Systems

characterization level	Pt_1_/CeO_2_ for thermal CO oxidation	Ni_1_/NC for electro CO_2_ reduction	Pt_1_/C_3_N_4_ for photocatalytic HER oxidation
primary environment	• EXAFS: Pt–O coordination number, bond distances	• XAS/EXAFS + wavelet analysis: Ni coordination number, bond distances	• EXAFS: Pt–N/C coordination number and bond distances
	• CO-FTIR/DRIFTS: discrimination Pt environments	• XANES/XPS: Ni electronic state	• XPS/XANES: Pt electronic state
	• XPS/XANES/HERFD: Pt electronic state	DFT: relative stability and electronic structure of candidates	DFT: relative stability and electronic structure of candidates
	• DFT: relative stability and electronic structure of candidates		
secondary environment	• HAADF/AC-STEM: Pt location relative to steps, defects, surface/subsurface	• HAADF/AC-STEM: Ni location relative to host features	• HAADF/AC-STEM: Pt location relative to framework sites/defects identifiable
	• higher-shell EXAFS: Pt–O–Ce/Pt–Ce connectivity	• XAS/EXAFS: higher-shell constraints on Ni–support connectivity	• XPS/EPR: C/N and defect chemistry constraining possible secondary environments
	• EPR/XPS/Raman/NMR: O-vacancy, Ce-redox, and O chemistry	• DFT: heteroatom/defect configurations and their influence	• DFT: coordination pockets, vacancy configurations and their influence
	• DFT: vacancy/polaron/O configurations and influence		
extended environment	• microscopy/diffraction: facet/step topology, crystallographic organization, and reconstruction	• XRD/TEM: carbon morphology and structural order/graphitization	• XRD/TEM: framework morphology, structural order, and layering
	• Raman/XPS/EPR/NMR: distributed O-vacancy, Ce-redox, and O-species populations	• XPS/electronic characterization: broader support chemistry and electronic properties	• XPS/EPR: distributed C/N chemistry and defect populations
	• surface characterization + DFT: reducibility, O mobility, lattice response, Ce 4*f* states	• interfacial characterization + DFT: conductivity and broader electrode–electrolyte organization	• valence-band spectroscopy: band structure and broader electronic redistribution
			• DFT: conjugation, electronic properties
dynamic and operando resolution	• operando XAS: redistribution of Pt environments	• operando XAS/vibrational spectroscopy: potential- and adsorbate-dependent evolution and redistribution of Ni environments	• XPS/DRIFTS: evolution of bonding and adsorbate-associated environments
	• DRIFTS: adsorbate-induced changes	• interfacial probes/modeling: potential-dependent solvent/electrolyte organization	• operando XAS: evolution and redistribution of Pt coordination and electronic environments
	• MD: fluctuations, interconversion, and lifetimes of accessible local environments		

**Table 8:** Structural and Electronic Dynamics, Catalytic Function, and Longer-Term Evolution of Local Environments under Reaction Conditions in Representative SAC Systems

environment behavior	Pt_1_/CeO_2_ for thermal CO oxidation	Ni_1_/NC for electro CO_2_ reduction	Pt_1_/C_3_N_4_ for photocatalytic HER oxidation
structural dynamics	• Pt–O coordination/geometry and Pt–O–Ce connectivity reorganize during reaction	• dynamic changes in CN, bond distances, geometry and out-of-plane position	• dynamic Pt coordination and Pt–support connectivity, including bond cleavage/reformation and adsorbate coordination
	• O/vacancy and adsorbate configurations dynamically restructure the environment	• Ni coordination modified by adsorbates/O/OH, hydrogenation, solvation	• coupled C/N-bonding and defect reorganization
	• reversible evolution between isolated and few-atom configurations can occur	• coordination changes and Ni migration generate multiple working configurations	
electronic dynamics	• dynamic Pt^4+^/Pt^2+^/Pt^δ+^/Pt^0^ character, coupled to Pt↔CeO_2_ charge transfer and Ce^3+^/Ce^4+^ redistribution	• potential/adsorbates modify Ni charge density, ligand↔metal charge transfer and electronic symmetry	• dynamic cationic ↔ highly reduced/Pt^0^-like character through Pt↔C_3_N_4_ charge transfer
	• structurally similar environments can access different electronic states	• Ni sites may or may not be reduced	• charge redistribution couples to Pt coordination and framework/defect states
catalytic function	• Pt-centered sites: CO adsorption/activation	• Ni–ligand environment: CO_2_ activation, intermediate stabilization and CO binding/release	• Pt center: photogenerated-electron capture/localization and H_2_O/H activation
	• Pt–O–Ce/CeO_2_ surroundings: reactive-O activation/supply	• support/interfacial and proton-containing surroundings: proton transfer, HER competition and adsorbate configuration	• C_3_N_4_ environment: charge generation, separation and transport
	• dynamic coordination, charge and vacancy configuration: adsorbate binding and access to reactive O		• Pt coordination/defect environment: H_2_O/H adsorption and activation
long-term evolution	• loss/reduction of highly coordinated Pt environments, access to lower-coordinate/reduced states	• persistent coordination-shell; ligand/hydrogenation and geometric changes can accumulate with operation	• largely preserved Pt–C/N coordination and atomic dispersion
	• Pt aggregation can occur; oxidative redispersion can drive toward isolated Pt	• weaker stabilization drives Ni migration and aggregation	• only slight reduction/local restructuring, without substantial aggregation

**Table 9:** Structural, Energetic, and Electronic Descriptors Linking Local Environments to Catalytic Behavior in Representative SAC Systems

environment descriptors	Pt_1_/CeO_2_ for thermal CO oxidation	Ni_1_/NC for electro CO_2_ reduction	Pt_1_/C_3_N_4_ for photocatalytic HER oxidation
structural descriptors	• Pt–O/Pt–Ce CN and distances	• Ni–N/C CN and coordination composition	• Pt–N/Pt–C CN and distances
	• Pt–O–Ce geometry	• Ni out-of-plane displacement	• Pt coordination geometry/location
	• Pt location	• defect/vacancy density	• N-vacancy density
	• O-vacancy density		
energetic descriptors	• Pt binding energy	• Ni binding energy	• Pt binding energy
	• O-vacancy formation energy	• Ni migration/restructuring barriers	• defect formation energy
	• CO adsorption energies	• potential-dependent free energies	• H_2_O/H* adsorption energies
	• Pt migration barrier		
electronic descriptors	• XANES white-line/edge features	• XANES white-line/edge features	• Pt core-level BE
	• Pt core-level BE	• Ni core-level BE	• Pt 5*d* occupancy
	• Ce^3+^ fraction	• Bader charge of Ni	• *d*-band center/DOS
	• Bader/transferred charge		

## 8 Outlook and Future Directions

Moving from individual case studies toward environment-resolved SAC design requires a roadmap that connects definition, characterization, comparison, and prediction (Figure 29). The central challenge is no longer only to establish that isolated atoms are present but also to determine which local environments they occupy, how those environments evolve under relevant conditions, and which fraction of the resulting site population contributes to catalysis. This shift is essential because nominally similar SACs can display different catalytic behavior when their primary coordination sphere, secondary environment, catalyst-derived context, or condition-dependent working state differ in ways that are not captured by a single structural assignment.

**Figure 29:**
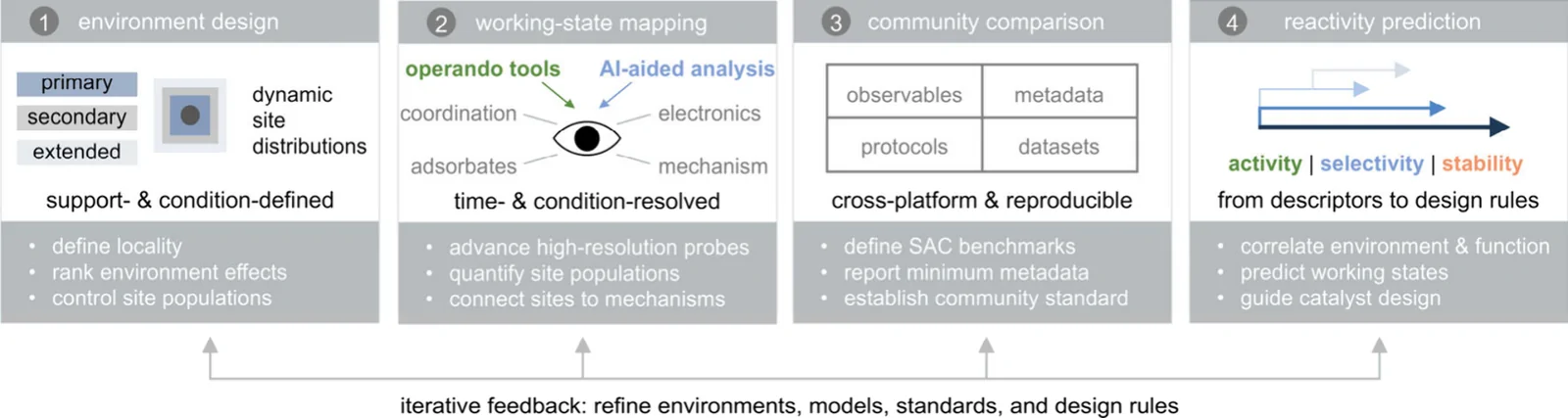
Roadmap for mapping local environment distributions to performance in SACs via operando characterization, theory, and data-driven descriptors.

The first step is the environment design. Future studies should define locality in terms of how primary, secondary, and extended influences affect isolated centers. This requires identifying which environmental features are most relevant for a given catalyst and reaction, including direct coordination, proximal heteroatoms or defects, confinement, support electronics, adsorbates, hydroxyl groups, electrolyte species, or interfacial organization. The aim is not to describe every surrounding feature but to rank those that measurably influence site structure, accessibility, adsorption, activation barriers, reaction pathways, or stability. Synthetic strategies should therefore be evaluated not only by whether they generate isolated atoms but also by how reproducibly they control the distribution of local environments formed during synthesis, activation, and operation.

This need becomes even more important as the field moves toward geminal, dual-atom, and more complex atomically dispersed architectures. In these systems, catalytic function may depend not only on the environment of each individual atom but also on the distance, symmetry, electronic communication, and cooperative dynamics between neighboring centers. The local environment framework must therefore evolve from describing isolated sites to describing coupled environments, while still distinguishing genuine cooperative motifs from statistical proximity or unresolved mixtures of sites.

The second step is the working-state mapping. Since SACs often adapt under operating conditions, characterization should move beyond assigning a single dominant ex situ structure toward identifying the populations of environments present during catalysis. Operando spectroscopy, microscopy, transient methods, theoretical modeling, and AI-aided analysis will be most powerful when they are used together to connect coordination, electronic structure, adsorbates, defect populations, and mechanistic states across relevant time and condition windows. A key goal is to distinguish productive reversible adaptation, in which isolated atoms sample related configurations while retaining catalytic function, from irreversible restructuring, aggregation, leaching, poisoning, or support degradation that lead to deactivation.

The third step is community comparison. Environment-resolved SAC design will require benchmark catalyst families, minimum metadata, shared observables, and comparable data sets. Benchmark systems should not be treated as universal standards, but as reference platforms for testing whether descriptors and design principles transfer across different supports, reactions, and operating regimes. M–N–C materials, oxide-supported single atoms, zeolite- or framework-confined sites, and electrochemical single-site catalysts provide complementary examples because they span different anchoring motifs, degrees of structural order, support dynamics, and exposure to gas-phase, liquid-phase, or electrochemical environments.

For such comparisons to be meaningful, studies must report enough information to reconstruct how the local environment was formed and tested. This includes precursor identity, ligand or counterion chemistry, support history, synthesis and activation protocols, metal content, site density, nuclearity, spatial distribution, reaction conditions, stability, and evidence for working-state evolution. Minimum metadata should also include the observables used to assign the environment, such as coordination number, bond distance, oxidation state, adsorbate signatures, defect populations, metal nuclearity, or support/interfacial restructuring. Without this information, apparent structure–performance relationships may reflect differences in unreported environment distributions rather than transferable catalytic principles.

The final step is performance prediction. The aim is not only to correlate isolated descriptors with activity, selectivity, or stability, but to identify environment–function relationships that can be translated into design rules. This requires connecting descriptor spaces to working-state populations and catalytic mechanisms, including the selectivity patterns and stability limits that emerge under realistic conditions. Predictive models will therefore need to account for both the most abundant environments and minority states that may dominate catalytic turnover, while distinguishing active sites from spectators or resting states.

Iterative feedback among environment design, working-state mapping, community comparison, and performance prediction will be essential. New mechanistic insight should refine which local features are considered relevant; improved characterization should refine the descriptors used in models; benchmark data sets should reveal which design principles are transferable; and performance trends should guide the synthesis of catalysts with better-controlled site populations.

This environment-centered view also has important implications for the translation of SACs into practical catalyst formulations. While promotion is well established across heterogeneous catalysis, comparatively few studies have resolved how promoters are spatially associated with atomically dispersed centers and how such associations modify their local environments.^230^ This challenge becomes increasingly relevant during scale-up, where binders, conductive components, stabilizers, cocatalysts, or other additives may be introduced for reasons extending beyond intrinsic catalytic activity.^374^ Such components may remain part of the extended catalyst environment or, when sufficiently associated with the active center, become integrated into its local environment. Determining when this occurs, how reproducibly these environments are formed, and whether they persist under operating conditions will be important for translating environment-resolved design from model SACs to practical catalysts. The field will advance most rapidly when “single atom” is treated not as a complete structural assignment, but as the starting point for defining, comparing, and ultimately designing the catalytic environment around isolated metal centers.
